# Cycloastragenol Derivatives Improve Tyrosine Metabolism, Regulate TLR4/NF‐κB/TERT Signaling Pathways, and Inhibit MPTP Induced Neuroinflammation and PD Symptoms

**DOI:** 10.1002/cns.70787

**Published:** 2026-02-11

**Authors:** Shengnan Xiao, Lianmei Liu, Xuemei Qin, Lei Xu, Zhenyu Li, Zhi Chai

**Affiliations:** ^1^ Institute of Taihang Materia Medica Shanxi University of Chinese Medicine Jinzhong China; ^2^ Modern Research Center for Traditional Chinese Medicine of Shanxi University Taiyuan China

**Keywords:** cycloastragenol derivative, neuroinflammatory, Parkinson's disease, TLR4/NF‐κB/TERT, tyrosine metabolism

## Abstract

**Background:**

Parkinson's disease (PD) is a neurodegenerative disease closely related to neuroinflammation and with obvious age characteristics. Existing therapeutic drugs have problems such as insufficient efficacy and side effects. Cycloastragenol (CAG) is a known natural telomerase activator, and previous studies have found that it has a good improvement effect on PD. As a lead compound, there is significant room for improvement in pharmacological activity. Therefore, we further explore safe and efficient small molecules for PD drug exploration through structural optimization.

**Methods:**

We introduced carboxylic acid small molecules into the CAG structure and evaluated the pharmacological effects of the derivatives using a PD in vitro model and a neuroinflammatory model. The structure–activity relationship analysis was used to screen the derivatives with the best activity for subsequent in vivo animal experiments. Utilize metabolomics and subsequent validation experiments to elucidate the potential mechanisms by which the derivatives exert their effects.

**Result:**

We screened and obtained compound R2 from 29 derivatives, which can significantly enhance anti‐inflammatory activity and cell protection. In the MPTP induced PD mouse model, R2 can improve motor dysfunction, restore the number of TH positive neurons in the substantia nigra, and reduce inflammation levels in brain tissue and serum. Metabolomics analysis showed that R2 intervenes in PD progression by regulating the tyrosine metabolism pathway, and further validated the mechanism of compound R2 around the TLR4/NF‐κB/TERT signaling pathway. This study provides a new strategy for the development of anti PD drugs based on CAG, while expanding the potential application of carboxylic acid modification in natural product structure optimization.

**Conclusions:**

This study focuses on the potential mechanism of CAG derivative R2 in treating PD through “inflammation‐aging” research. By inhibiting inflammation and restoring telomerase activity, it breaks the vicious cycle and provides new ideas for the treatment of PD, laying a foundation for the development of drugs using CAG for PD.

## Graphical abstract

Cycloastragenol derivatives regulate the TLR4/NF‐κB/TERT signaling pathway and tyrosine metabolism, breaking the vicious cycle of “inflammation‐aging” and improving symptoms of Parkinson's disease. This study provides direction for the application of natural products in the development of PD drugs.

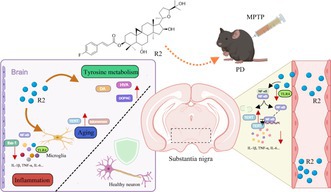

**Abbreviations**
BV‐2: microglial cell line
CCK‐8: cell counting kit‐8
IL‐1β: interleukin‐1β
IL‐6: interleukin‐6
LDH: lactatedehydrogenase
L‐DOPA: levodopa
LPS: lipopolysaccharide
MPP^+^
: 1‐methyl‐4‐phenylpyridinium
MPTP: 1‐methyl‐4‐phenyl‐1,2,3,6‐tetrahydropyridine
NF‐κB: nuclear factor kappa‐B
SH‐SY5Y: human neuroblastoma cell line
TERT: telomerase reverse transcriptase
TLR4: toll‐like receptor 4
TNF‐α: tumor necrosis factor‐α

## 1 Introduction

Parkinson's disease (PD) is the second most common neurodegenerative disease in the world. The domestic epidemiological survey shows that the incidence rate of PD among people over 60 years old reaches 2% and is increasing year by year [1]. Due to the complex pathogenesis of PD, including oxidative stress, neuroinflammation, mitochondrial dysfunction, as well as genetic and environmental factors [2]. Therefore, there is currently no cure for PD, and first‐line clinical drugs such as levodopa are used to alleviate movement disorders, making it difficult to fundamentally address the progressive loss of neurons. Meanwhile, long‐term use can lead to reduced efficacy, immune suppression, and other adverse reactions, affecting the improvement of quality of life [3, 4]. Therefore, developing safer and more effective therapeutic drugs is an urgent problem to be solved. Neuroinflammation is a common feature among the pathogenic factors of neurodegenerative diseases. Inhibiting the excessive development of neuroinflammation can reverse neuronal damage in neurodegenerative diseases and has become a hot topic in new drug development [5, 6]. Natural products have become an important source for drug development due to their strong activity and low toxicity [7, 8]. Therefore, it is very important to develop novel neuroinflammatory inhibitors for PD drug development in natural products.

*Astragalus membranaceus*, as a classic nourishing medicine in traditional Chinese medicine, is widely used in the treatment of neurodegenerative diseases such as PD in traditional Chinese medicine [9, 10, 11]. Modern pharmacological research has shown that its main functional component, Astragaloside IV, has been proven to have inhibitory effects on neuroinflammation and alleviate behavioral indicators of Parkinson's disease [12]. Cycloastragenol (CAG) is the glycoside structure of Astragaloside IV, which has various pharmacological activities, including anti‐inflammatory, antiaging, and immune regulation [13, 14]. In preliminary exploratory studies, we found that CAG can regulate the TLR4/NF‐κB signaling pathway, reduce the expression of pro‐inflammatory cytokines in the brain, and regulate the amino acid metabolism pathway to jointly inhibit the neuroinflammatory level of microglia and exert the regulatory effect of PD [15]. Other studies have shown that CAG can reduce the assembly of NLRP3 inflammasomes and exert neuroinflammatory inhibitory effects in PD in vivo models [16]. In addition, at a dose of 20 mg/kg, CAG has a significant neuroprotective effect on ischemic brain injury in MACO mice by indirectly upregulating SIRT1, suppressing neuroinflammation [17]. Numerous studies have fully demonstrated that CAG mainly focuses on inhibiting neuroinflammation and improving the pathological state of neurodegenerative diseases [18]. However, in numerous in vitro and in vivo studies, we found that there is significant room for improvement in the activity of CAG in inhibiting neuroinflammation. Therefore, we hope to modify its structure and discover derivatives with better activity for drug exploration in PD.

Structural modification is an effective strategy to enhance the activity of lead compounds. Through structural analysis, it was found that CAG has alcohol hydroxyl structures at positions C‐3, C‐6, and C‐16, making it a potential active site. In recent years, the structural modifications of CAG have mainly focused on acetylation, glycosylation, ring opening reactions, and oxidation reactions [19, 20]. It is not difficult to see that the C‐3 and C‐6 hydroxyl groups are the main binding sites. In 2023, Professor Zhang Weidong's team obtained the CAG derivative HHQ16 through structural modification, which effectively reverses hypertrophy and heart failure induced by myocardial infarction with a new target [21]. This sensational achievement has attracted the attention of many scholars to CAG, indicating that the C‐3 hydroxyl group is an effective structural modification site. Meanwhile, as a tetracyclic triterpenoid compound, CAG shares structural similarities with ginsenosides. In the design and synthesis of similar structures, a large number of research results have been obtained by introducing different functional groups into the C‐3 hydroxyl group, which further demonstrates the feasibility of using the C‐3 hydroxyl group as a structural modification site [22, 23].

Introducing a carboxylic acid to form an ester bond at the C‐3 hydroxyl group is a prodrug strategy or a controllable physicochemical property modification strategy [24]. It can temporarily and reversibly alter the physicochemical properties of the parent compound, thereby overcoming bottlenecks in its pharmacokinetics or delivery [25]. The introduction of carboxylic acid groups increases structural diversity, ranging from acetic acid and propionic acid to fatty acids, amino acid esters, or targeting molecules, which provides enormous room for optimization [26]. In addition, the introduction of carboxylic acid groups can significantly enhance the penetration potential. Esterification of the C‐3 hydroxyl group increases the logP value of the compound, which theoretically can significantly improve its ability to cross the BBB passively, thus facilitating the development of central nervous system drugs [27].

In the structural optimization of similar compounds, carboxylic acid structures have also become the preferred choice and achieved good results. Derivatives such as panaxadiol and protopanaxadiol, combined with amino acids, benzoic acid, and fatty acids, exhibit enhanced activity in anti‐inflammatory, antitumor, and hypoglycemic effects [28, 29]. We introduce different types of carboxylic acid structures into CAG in the hope of obtaining molecules with enhanced activity. Therefore, in this study, we used CAG as the lead compound and introduced fatty acids, amino acids, salicylic acid, and cinnamic acid at the C‐3 position, and screen potent derivatives using PD in vitro cell models and neuroinflammation cell models. Combined with PD in vivo models, the in vivo efficacy of the compounds is verified, and the mechanism of action of the compounds is preliminarily explored, providing a scientific basis for the drug exploration of CAG for neuroinflammation or neurodegenerative diseases.

## 2 Materials and Methods

### 2.1 Chemicals and Reagents

Cycloastragenol (HPLC > 98%), MPTP, and MPP^+^ were purchased from Sigma‐Aldrich (Saint Louis, Missouri, USA). Chemical reagents were purchased from Macklin (Shanghai, China) or Aladdin (Shanghai, China). Elisa Kit was purchased from MeiMian (IL‐1β, IL‐6, TNF‐α) (Jiangsu, China) and Coibo Bio (Tyrosine, Phenylalanine, DA, DOPAC, and HVA) (Shanghai, China). The antibodies used in the Western blot assay were purchased from Servicebio (Wuhan, China), Wanleibio (Shenyang, China), and Abcam (Cambridge, UK). RapRIV was purchased from MedChemExpress (Shanghai, China).

### 2.2 Preparation of Target Compounds

The synthesis method of A1–A4, B1–B4, C1–C11, R1–R4 and S1–S2 was as follows: The CAG was dissolved in dichloromethane. Then 1‐(3‐Dimethylaminopropyl)‐3‐ethylcarbodiimide (EDC, material ratio = 1:2), carboxylic acid (material ratio = 1:2) and 4‐dimethylaminopyridine (DMAP, material ratio = 1:1) were added in sequence, stirred at room temperature for 2–24 h. Compound were obtained by column chromatography (The silica gel is 300–400 mesh). The separation conditions were dichloromethane: methanol = 30–40:1 or petroleum ether: acetone = 5:1.

For example, the preparation of compound R2: CAG (1.0 mmol, 1 equiv) was dissolved in dichloromethane (20 mL). Then, add EDC (2.0 mmol, 2 equiv) and 4F cinnamic acid (2.0 mmol, 2 equiv). After adding DMAP (1.0 mmol, 1 equiv), the reaction was carried out at room temperature for 8 h. Add water to terminate the reaction, extract to obtain a dichloromethane solution, and remove the solvent under reduced pressure to obtain the product to be separated. Compound R2 was isolated by silica gel column chromatography under the condition of petroleum ether: acetone = 5:1.

The synthesis method of A5–A8 was as follows: The A1–A4 (100 mg) were dissolved in dichloromethane, CF_3_COOH (100 μL) was added in sequence, stirred at room temperature for 2–4 h, A5–A8 were obtained by column chromatography with dichloromethane: methanol = 10–30:1.

### 2.3 In Vitro Assay

Cell viability and LDH assay were evaluated using a PD in vitro model (inducing SH‐SY5Y cells with 3 μM MPP^+^). All compounds (detected at a concentration of 10 μM) were cotreated with MPP^+^ for 24 h, CCK‐8 solution was added to each well and incubated at 37°C for 1 h, and the absorbance was measured at 450 nm using a Microplate reader; According to the detection requirements of the LDH assay kit, the cell supernatant of each group was collected and added to the detection reagent. The absorbance value was measured at 450 nm, and the LDH content in each group was calculated.

#### 2.3.1 IL‐1β Inhibition Assay

BV2 cells were uniformly seeded in 96‐well plates and stimulated with LPS (1 μg/mL) to establish a neuroinflammation model. All compounds (including CAG) were administered to the cells at a concentration of 10 μM for 24 h. Subsequently, the supernatants were collected and assayed for IL‐1β content using an IL‐1β kit, with absorbance measured at 450 nm. The IL‐1β levels of each group were analyzed to calculate the inhibition rate, and each compound was tested in triplicate.

In addition, for the experiment to determine the IC_50_ values of compounds CAG and R₂ against IL‐1β, the neuroinflammation model was established using the same method. The cells were treated with serial concentrations of CAG and R_2_ (2.5, 5, 10, 20, and 40 μM) for 24 h. The supernatants were harvested, absorbance was detected at 450 nm, and the IL‐1β inhibition rate and IC_50_ values were calculated accordingly.

#### 2.3.2 Morphological Observation

BV2 cells were uniformly cultured in a 6‐well plate, and LPS (1 μg/mL) induced BV2 cells to construct a neuroinflammatory model. Compound R2 (10, 20 μM) and CAG (20 μM) intervened in the model cells for 24 h, and the cell morphology was observed using a phase contrast microscope.

#### 2.3.3 TUNEL Assay

The TUNEL method is also used to detect R2 inhibition of MPP^+^‐ induced apoptosis in SH‐SY5Y cells. SH‐SY5Y cells grew uniformly in a 12 well plate. Treat cells with different concentrations (10 and 20 μM) of R2 and MPP^+^ for 24 h. Wash the cells with PBS buffer and fix them with 4% formaldehyde for 30 min. Add 50 μL of TUNEL detection solution to each well, incubate the cells in the dark at 37°C for 60 min, and observe the fluorescence intensity by taking pictures under a fluorescence microscope.

#### 2.3.4 ROS Detection

2′,7′‐dichlorofluorescein diacetate (DCFH‐DA) is used to detect the expression of reactive oxygen species (ROS). SH‐SY5Y was cultured in a 6‐well plate. Compounds R2 (10 and 20 μM) and MPP^+^ were cultured in 6‐well plates for 24 h. 400 μL of DCFH‐DA (10 μmol/L) was incubated with cells in the dark at 37°C for 30 min. Finally, wash the cells three times with serum‐free DMEM and analyze the expression of ROS between each group using flow cytometry.

### 2.4 Animal

A total of 60 male C57BL/6 mice, aged 6–8 weeks and weighing 20 ± 2 g, were procured from Vital River Company (Beijing, China). All experimental procedures involving the mice were reviewed and approved by the Animal Experiment Ethics Committee of Shanxi University of Chinese Medicine, with the approval number AWE202307367. The experiment is divided into 6 groups (*n* = 10): Control, MPTP, CAG (MPTP + CAG 30 mg/kg), R2‐L (MPTP + R2 10 mg/kg), R2‐H (MPTP + R2 30 mg/kg), and L‐DOPA (MPTP + L‐DOPA 70 mg/kg). MPTP was used to construct a PD mouse model, as shown in Figure 2A, for a total of 7 days. Day 1 (15 mg/kg i.p.), Day 2 (20 mg/kg i.p.), Day 3–7 (30 mg/kg i.p.). Each experimental group was given their own drug treatment for 14 days at the beginning of modeling.

### 2.5 Behavioral Test

Gait assay and pole test were performed following the experimental methods described in previous studies [15]. Hanging test: Hang the mouse's front paws on a horizontal steel wire, and score based on the number of final mouse hind paws hanging on the wire. The rule is as follows: two hind paws (3 points); one hind paw (2 points); the hind paw is zero (1 point); mouse landing (0 point). All experiments were repeated three times, with a 10 min interval between each iteration.

### 2.6 ELISA Analysis

Brain tissue or blood samples from each group were collected first, followed by supernatant extraction. Subsequently, ELISA was conducted separately to detect the inflammatory factors IL‐1β, IL‐6, TNF‐α, Tyrosine, Phenylalanine, DA, DOPAC, and HVA. Absorbance values were measured at a wavelength of 450 nm, and these values were used to analyze the expression levels of the aforementioned inflammatory factors.

### 2.7 Immunohistochemical, Immunofluorescence Staining and H&E Analysis

Three mouse brain tissues were dehydrated using a gradient of sucrose solution and embedded with OCT reagent. Freeze the brain tissue in liquid nitrogen and use a low‐temperature constant temperature slicer to cut coronal sections (10 μm). First, slice the samples and dry them for 24 h, after which store the dried slices at −80°C. Next, rinse the slices with PBS to remove the OCT embedding medium. Subsequently, submerge the slices in PBS supplemented with 0.3% Triton X‐100, and incubate the mixture at room temperature for 30 min. Immunohistochemical staining: Add an appropriate amount of first antibody (TH, Iba‐1, CD68 and CD11B) onto the slice, incubate overnight, and wash with PBS to evenly distribute low valent secondary antibody. Finally, counterstain with hematoxylin staining solution, observe and analyze the results. Immunofluorescence staining: Apply primary antibodies (TH, Iba‐1, and TERT) uniformly to the sections and allow overnight incubation. Next, incubate the sections with secondary fluorescent antibodies. Conclude by adding DAPI in a dropwise manner and incubating for 5–10 min. Visualize and capture images using a confocal laser scanning microscope, then quantify fluorescence intensity with Image‐J software. H&E analysis: Liver tissues from each group of mice were collected, embedded in paraffin, sliced, and subjected to pathological analysis based on H&E staining.

### 2.8 Western Blotting

Brain tissue and cell samples from various groups were harvested, homogenized in RIPA lysis buffer, and total proteins were extracted. Following separation via SDS‐PAGE, proteins were transferred onto membranes. Polyvinylidene fluoride membranes were blocked with 5% skim milk, then individually incubated overnight at 4°C with primary antibodies against TH, IL‐1β, IL‐6, TNF‐α, NF‐κB, p‐NF‐κB, TLR4, TERT, p53, p16, p21, and β‐actin (1:1000 dilution). After washing with TBST, membranes were incubated with antirabbit secondary antibody (1:5000 dilution). Finally, immunoreactive signals were detected and captured using a gel imaging system.

### 2.9 Telomerase Activity Assay

After grinding mouse brain tissue, add the corresponding dose of TRAP real‐time quantitative telomerase activity detection kit (Genmed, USA) provided lysis buffer, extract proteins, detect the protein concentration of each group, and dilute the sample protein concentration uniformly. Add 1 μg of protein to each reaction and use qRT‐PCR to detect Ct values in each group according to the kit requirements, and the relative expression level was analyzed using the 2^− ΔΔCT^ method.

### 2.10 LC–MS Analysis of Intestinal Contents

Intestinal contents were collected from the Control, Model, and R2‐H groups (*n* = 6). Sample processing and LC–MS analysis were entrusted to Shanghai Biotree Biotech Co. Ltd., encompassing differential metabolite identification, differential substance analysis (including cluster analysis and KEGG pathway analysis), as well as univariate and multivariate statistical analyses. Ultimately, differential substances between Con versus Mod and Mod versus R2 groups were identified, and their associated metabolic pathways were analyzed.

### 2.11 Statistical Analysis

Group comparisons were statistically analyzed using one‐way ANOVA with subsequent Tukey's post hoc testing. All data are presented as the mean ± standard deviation (SD) from three independent replicate experiments. Statistical significance was defined as a *p*‐value < 0.05.

## 3 Results

### 3.1 A Total of 29 Carboxylic Acid Derivatives of CAG Were Obtained

According to Scheme 1, 29 compounds were synthesized for this study. CAG and appropriate carboxylic acid groups were dissolved in dry dichloromethane, and EDC and DMAP were added to obtain compounds A1–A4, B1–B4, C1–C11, R1–R4 and S1–S2. A5–A8 is obtained by deprotection of A1–A4 with trifluoroacetic acid. The ^1^H‐NMR, ^13^C‐NMR, and HRMS for all compounds were provided in the Figures S1–S87.

**SCHEME 1:**
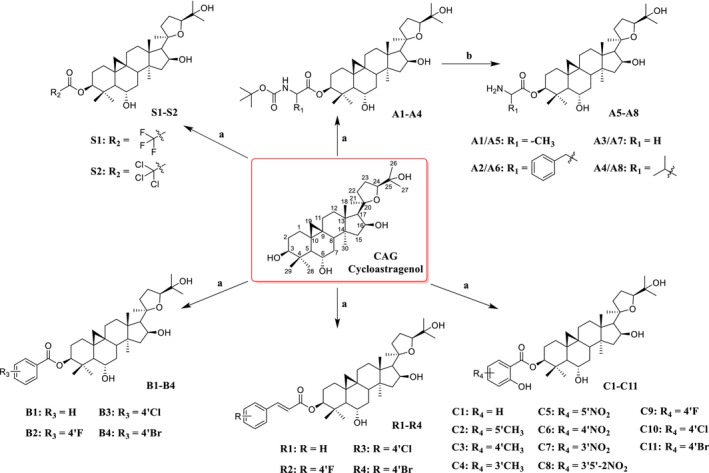
The synthesis routes of all compounds. Reaction conditions: (a) Carboxylic acid groups, EDC/DMAP, DCM, rt., 2–24 h; (b) CF_3_COOH, DCM, 2–4 h.

The spectral data shows that the hydroxyl group (C‐3) of CAG has been replaced. In the ^13^C‐NMR spectrum, the signals changed from δ C 78.0–79.0 (C‐3) to δ C 81.0–82.0 (C‐3). In the ^1^H‐NMR spectra, the signals changed from δ H 3.2 (H‐3) to δ H 4.5–4.6 (H‐3). By combining the spectra of carboxylic acid compounds, we can accurately determine the structure of derivatives based on CAG.

### 3.2 In Vitro Activity Evaluation and SAR Analysis

Two cell models [MPP^+^ induced SH‐SY5Y cells (in vitro PD model) and LPS induced BV2 cells (in vitro neuroinflammation model)] were used to investigate the cell viability, cytotoxicity, and IL‐1β inhibition of compound treated cells. Studies have shown that the biological activity of compounds (A5–A8) obtained by deprotection of amino acid derivatives is significantly stronger than that of their corresponding parent compounds (A1–A4). This result suggests that the BOC protecting group, due to its certain polarity and large steric hindrance, may hinder the effective binding of the molecule to the active site; in contrast, the free amino groups exposed after deprotection can act as hydrogen bond donors or acceptors, enhancing the interactions with the target and thereby improving the activity.

In the comparison between benzoic acid derivatives (B1–B4) and cinnamic acid derivatives (R1–R4), the introduction of the α, β‐unsaturated carbonyl structure significantly enhanced the anti‐inflammatory activity and cell viability of the latter, indicating that this structural motif plays a crucial role in regulating biological activity. Further structural exploration of benzoic acid analogs revealed that salicylic acid derivatives (C1–C11) exhibited a similar level of activity to benzoic acid derivatives, while some derivatives showed increased toxicity and exacerbated inflammatory responses (Table 1). This suggests that the position and properties of substituents such as hydroxyl groups on the benzene ring may exert a vital impact on safety and efficacy.

**TABLE 1:** In vitro activity evaluation of CAG derivatives.

Compounds^a^	Cell viability (%)	IL‐1β inhibition rate% (%)	LDH inhibition (U/mL)
A1	52.77 ± 2.48	14.27 ± 2.16	133.05 ± 2.03
A2	53.66 ± 1.39	12.37 ± 0.81	176.31 ± 1.34
A3	50.39 ± 2.78	7.33 ± 0.59	148.33 ± 3.29
A4	58.94 ± 2.55	8.64 ± 2.03	163.27 ± 2.45
A5	66.94 ± 1.69	11.07 ± 0.37	136.21 ± 1.39
A6	64.37 ± 2.81	10.08 ± 1.21	125.48 ± 2.59
A7	73.46 ± 2.07	17.33 ± 0.27	118.78 ± 2.19
A8	70.38 ± 1.01	15.22 ± 2.17	117.31 ± 1.82
B1	40.39 ± 1.85	10.28 ± 1.44	215.34 ± 2.88
B2	52.37 ± 3.29	6.58 ± 2.13	156.78 ± 1.44
B3	54.88 ± 1.82	12.44 ± 0.59	136.59 ± 2.76
B4	49.29 ± 2.14	14.66 ± 1.73	179.16 ± 3.46
C1	70.59 ± 1.77	18.55 ± 1.09	105.39 ± 3.62
C2	58.44 ± 3.12	22.39 ± 2.11	154.88 ± 2.18
C3	62.58 ± 2.54	6.55 ± 1.32	114.41 ± 1.58
C4	60.33 ± 2.61	12.35 ± 0.66	118.79 ± 2.54
C5	34.69 ± 2.59	< 0	243.18 ± 4.57
C6	28.31 ± 2.74	< 0	251.34 ± 1.53
C7	30.41 ± 1.28	< 0	217.33 ± 3.63
C8	25.64 ± 3.77	< 0	196.48 ± 2.44
C9	51.47 ± 1.59	10.22 ± 0.84	168.49 ± 1.68
C10	58.34 ± 2.57	9.23 ± 0.88	146.28 ± 2.51
C11	56.49 ± 2.63	1.07 ± 0.81	134.77 ± 1.61
R1	70.19 ± 1.48	42.58 ± 1.57	78.36 ± 3.17
R2	86.74 ± 2.22	50.38 ± 1.05	74.28 ± 2.41
R3	80.28 ± 1.64	36.54 ± 0.68	88.34 ± 2.75
R4	75.34 ± 1.04	36.28 ± 0.44	80.47 ± 1.79
S1	62.33 ± 2.47	22.44 ± 0.78	125.37 ± 2.13
S2	64.77 ± 3.17	8.64 ± 0.39	150.22 ± 2.18
CON	100 ± 4.28	—	42.38 ± 2.39
MPP^+^	66.28 ± 2.34	—	152.77 ± 3.68
LPS	—	0	—
CAG	72.68 ± 1.47	23.58 ± 1.08	123.17 ± 0.48

^a^ The test concentration of the compound is 10 μM.

In summary, the SAR analysis demonstrated the following: (1) Removal of the BOC protecting group can enhance binding affinity by reducing steric hindrance and providing hydrogen bonding sites; (2) The introduction of the α, β‐unsaturated carbonyl moiety is a key structural modification to improve anti‐inflammatory activity and cell viability, and its effect may be associated with enhanced electronic conjugation, improved membrane permeability, or involvement in covalent interactions; (3) Although the salicylic acid scaffold retains certain activity, subtle differences in substituents can lead to alterations in toxicity and inflammatory responses, indicating that a balance between efficacy and safety must be comprehensively considered during activity optimization.

**SCHEME 2:**
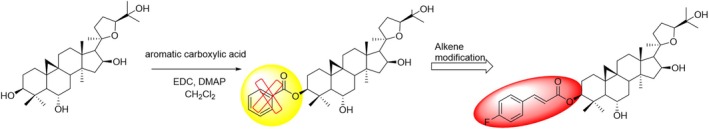
Exploration of Structure Activity Relationship.

These findings highlight that the introduction of the olefin moiety plays a decisive role in activity enhancement, and provide a direction for subsequent systematic structural optimization and derivatization studies targeting this pharmacophore (Scheme 2).

**FIGURE 1:**
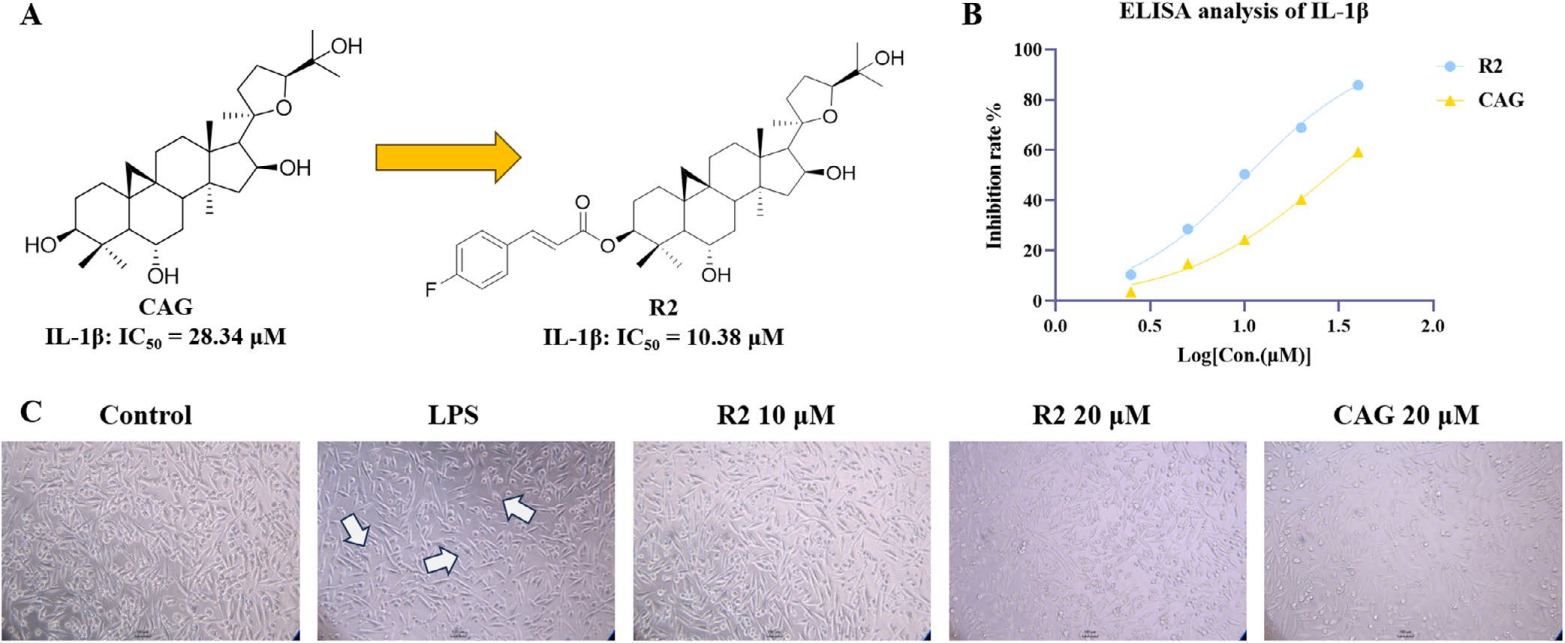
In vitro activity evaluation of compound R2. (A) The structure of compound R2; (B) Inhibition rate of IL‐1β by compound R2 and CAG; (C) Morphological observation of compound R2 inhibiting LPS induced inflammatory response in BV2 cells. (The scale is 100 μm).

Based on the analysis of the results, we found that compound R2 has the best biological activity and can effectively reverse the risks brought by MPP^+^ and LPS to cells. In further activity confirmation, we found that compound R2 had an IC_50_ value of 10.38 ± 0.54 μM for inhibiting IL‐1β, which was significantly stronger than CAG (IC_50_^IL‐1β^ = 28.34 ± 1.27 μM) (Figure 1A,B). In the observation of cell morphology, compound R2 could significantly inhibit the activated morphology such as cell body enlargement caused by LPS (Figure 1C). Therefore, we chose compound R2 for subsequent in vivo activity evaluation and mechanism research. In addition, we induced SH‐SY5Y cells with MPP^+^ and evaluated the effect of compound R2 on cell apoptosis and reactive oxygen species (ROS) expression. The results showed that compound R2 can effectively inhibit the increase in ROS expression and cell apoptosis caused by MPP+, indicating that compound R2 has a good neuroprotective effect (Figure S88).

### 3.3 Compound R2 Improves the Behavioral and Pathological Indications of PD

**FIGURE 2:**
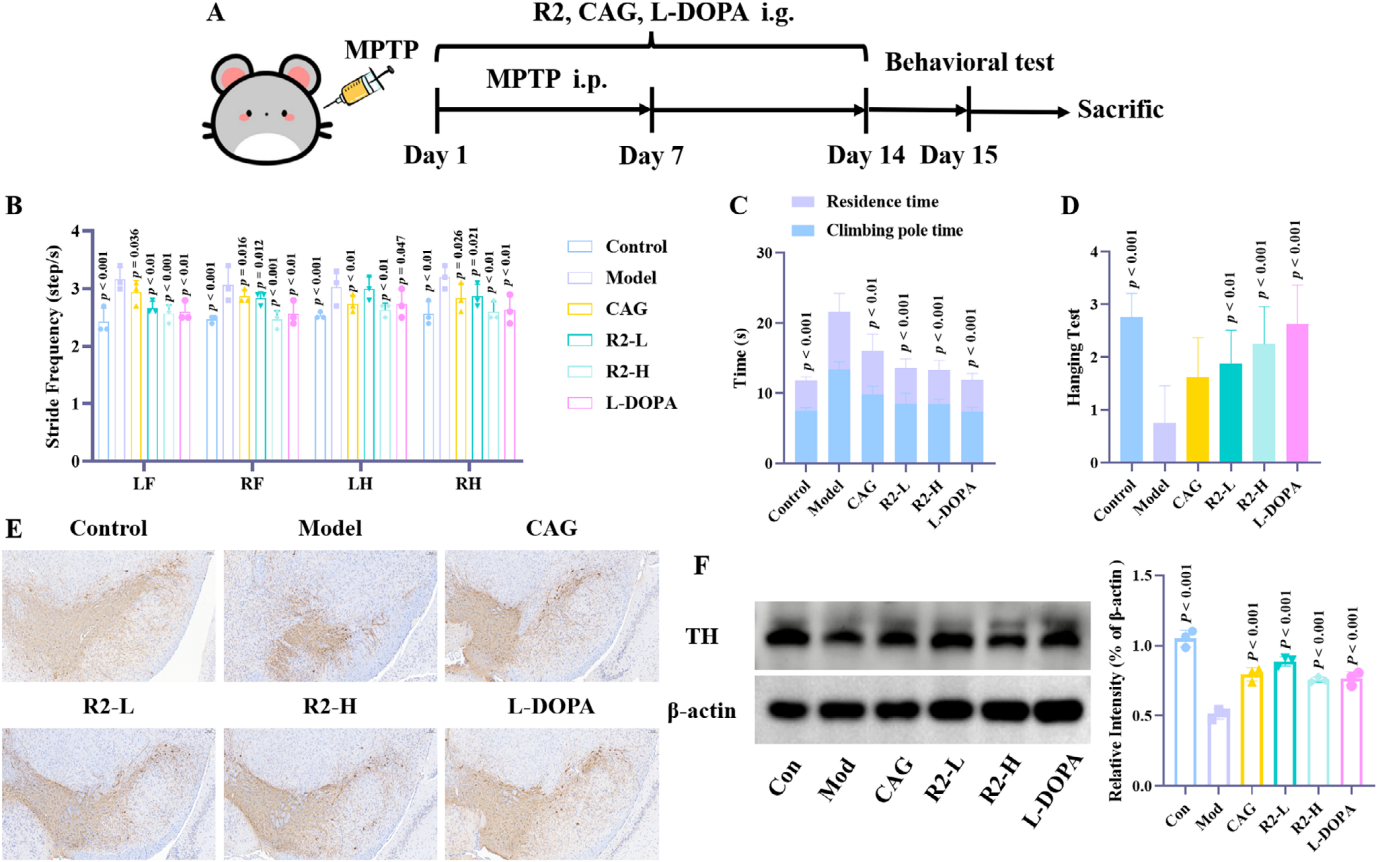
Compound R2 improves the behavioral and pathological indications of PD. (A) Method of MPTP induced PD model; (B) Step frequency testing in gait assays. LF, left front paw; LH, left hind paw; RF, right front paw; RH, right hind paw; (C) The Pole test; (D) Hanging test; (E) Immunohistochemical assay were conducted to observe the number of TH positive cells in the substantia nigra after R2 treatment. (The scale is 100 μm); (F) TH protein expression level detected by Western blot. (*p* vs. Model group).

In this study, a classic MPTP‐induced mouse model of PD was established to evaluate the in vivo behavioral effects of compound R2 (see Figure 2A). Behavioral test results demonstrated that compound R2 could effectively reverse MPTP‐induced motor dysfunction, with specific manifestations as follows:

#### 3.3.1 Gait Assay

Compared with the Control, mice in the MPTP group exhibited significant gait abnormalities with a marked increase in stride frequency, indicating typical motor hastening and coordination impairment. After intervention with compound R2, the stride frequency of mice decreased significantly and recovered to near‐normal levels, suggesting that R2 effectively ameliorated gait disorders induced by the PD model (Figure 2B).

#### 3.3.2 Pole Test

In the pole test, which evaluates motor coordination and endurance, mice in the MPTP group showed a significant reduction in pole‐climbing speed and a marked prolongation of residence time at the top of the pole, reflecting severe motor bradykinesia and hesitation. In contrast, mice in the R2 group displayed a notable increase in pole‐climbing speed along with a significant shortening of residence time at the pole top. Collectively, these results indicated that R2 intervention effectively alleviated MPTP‐induced motor initiation retardation and movement hesitation (Figure 2C).

#### 3.3.3 Hang Test

In the hang test, which reflects limb muscle strength and endurance, the grip strength and climbing ability of mice in the MPTP group were significantly impaired, with a decreased proportion of effective climbing time. Following R2 treatment, the suspension and climbing abilities of mice on the grid were significantly improved, showing more sustained grip strength and more active climbing movements. This suggested that R2 exerted a positive ameliorative effect on muscle weakness and motor fatigue associated with the PD model (Figure 2D).

Behavioral experiments have fully demonstrated that compound R2 can significantly improve behavioral symptoms in mice, and its effect is comparable to that of L‐DOPA. In addition, the decrease in the number of TH positive cells in the substantia nigra is a typical pathological feature of the PD model. Immunohistochemical assay showed that after MPTP administration, the number of TH positive cells in the substantia nigra of mice significantly decreased and was significantly reduced after R2 treatment, indicating that compound R2 can effectively inhibit progressive neuronal damage (Figure 2E). Similarly, we obtained the same results in the Western blot (Figure 2F).

In addition, we performed H&E staining on the liver tissues of mice in each group to evaluate the toxic effects of compound R2 on mice during the 14‐day treatment cycle. The results showed that the hepatocyte morphology of mice was normal across all groups, with no inflammatory damage or similar abnormalities observed. These findings indicate that treatment with compound R2 at this dosage for 14 days exerted no or minimal toxic effects on mice (Figure S89).

### 3.4 Compound R2 Effectively Inhibits Neuroinflammation in In Vitro and In Vivo Models

**FIGURE 3:**
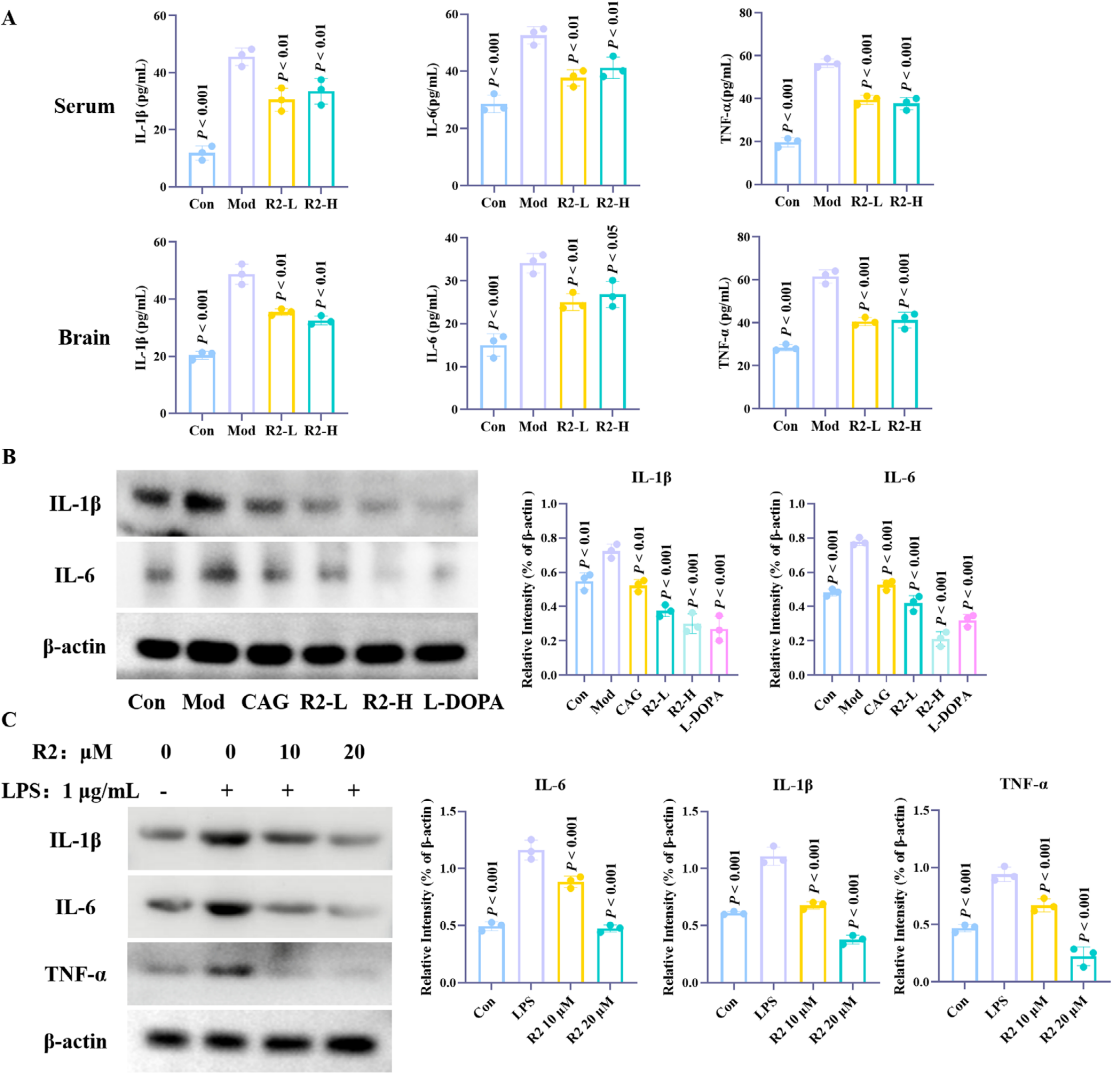
Compound R2 inhibits inflammation levels in in vitro and in vivo models. (A) Expression of inflammatory factors in brain tissue and serum by ELISA; (B) Expression of inflammatory factors in brain tissue by Western blot; (C) Inhibition of inflammation related protein expression by compound R2 in LPS induced BV2 cell model. (*p* vs. Model group).

We further evaluated the level of neuroinflammation after R2 administration. In animal models, ELISA was used to detect the release of inflammatory factors in brain tissue and serum. The results showed that after MPTP induction, varying degrees of inflammatory reactions were observed in both brain tissue and serum of mice, and the expression of inflammatory factors significantly increased. After treatment with compound R2, there was a significant recovery (Figure 3A). Further evidence in Western blot experiments showed that compound R2 can effectively inhibit the protein expression of IL‐1β and IL‐6 (Figure 3B). Similarly, we validated the pharmacological effects of the compound using an in vitro model of neuroinflammation (LPS induced BV2 cells) and obtained the same conclusions as the animal model (Figure 3C). Therefore, we conclude that compound R2 alleviates the symptoms of PD by improving neuroinflammation.

### 3.5 Compound R2 Can Inhibit the Activation of Microglia

**FIGURE 4:**
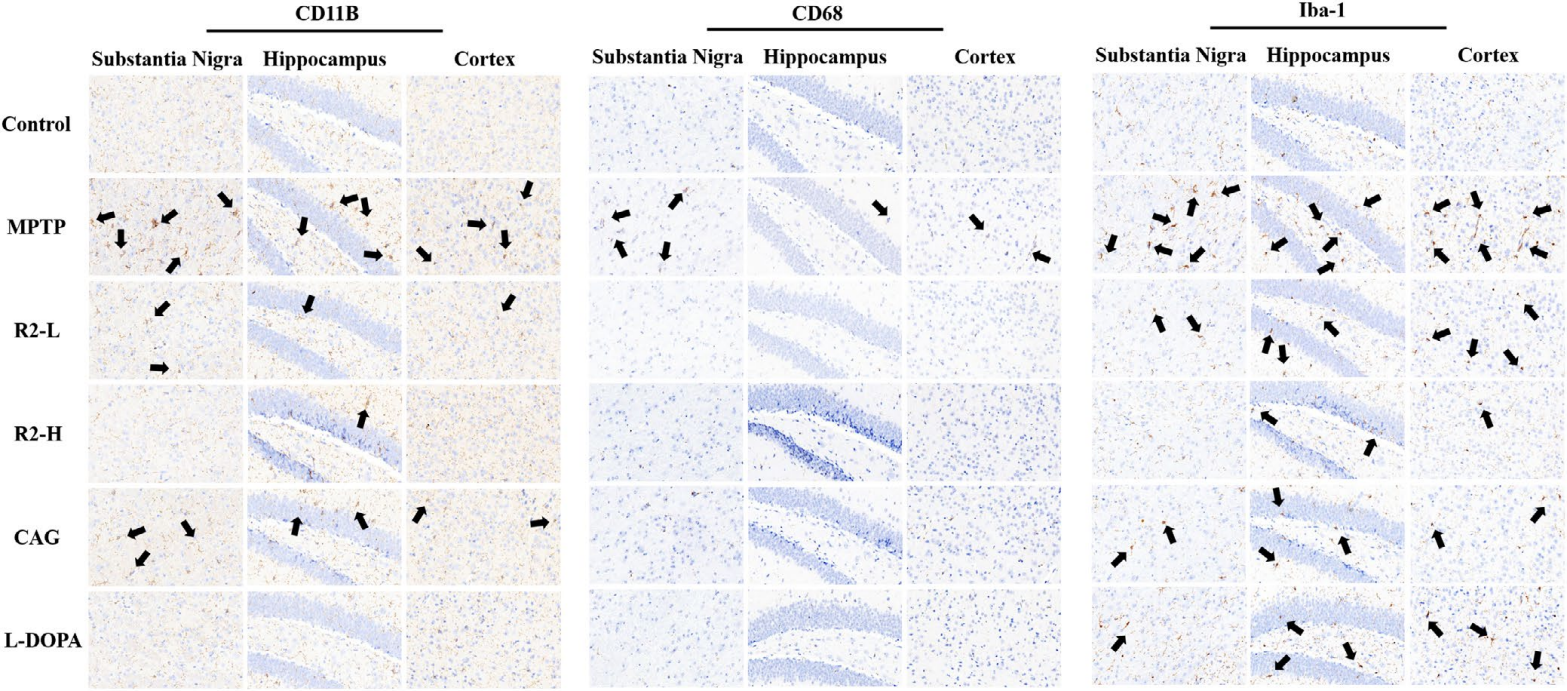
Compound R2 can inhibit the activation of microglia. Analyze the expression of CD118, CD68, and Iba‐1 between different groups in the substantia nigra, hippocampus, and cortex. Using the number of spots expressed by antibodies as a statistical method, analyze the differences between groups. (The scale is 100 μm).

Neuroinflammation mainly involves the immune response of the central nervous system, and the activation of microglia is a key link in neuroinflammation. Furthermore, we detected key indicators of microglial activation and found that in the model group, the expression of CD68 was slightly upregulated, while the expression levels of Iba‐1 and CD11B were significantly increased, indicating that microglia were in an activated state. In addition, this activated state was not limited to the substantia nigra region. We also analyzed the cerebral cortex and hippocampus and obtained the same results. In the treatment group, the R2‐H group showed the most significant therapeutic effect, significantly stronger than other groups and comparable to the L‐DOPA group. Therefore, compound R2 can effectively inhibit MPTP‐induced activation of microglia and reduce inflammation levels (Figure 4).

### 3.6 Compound R2 Improves Neuroinflammation in PD Models by Regulating Tyrosine Metabolism and Is Potentially Associated With the NF‐κB Signaling Pathway

**FIGURE 5:**
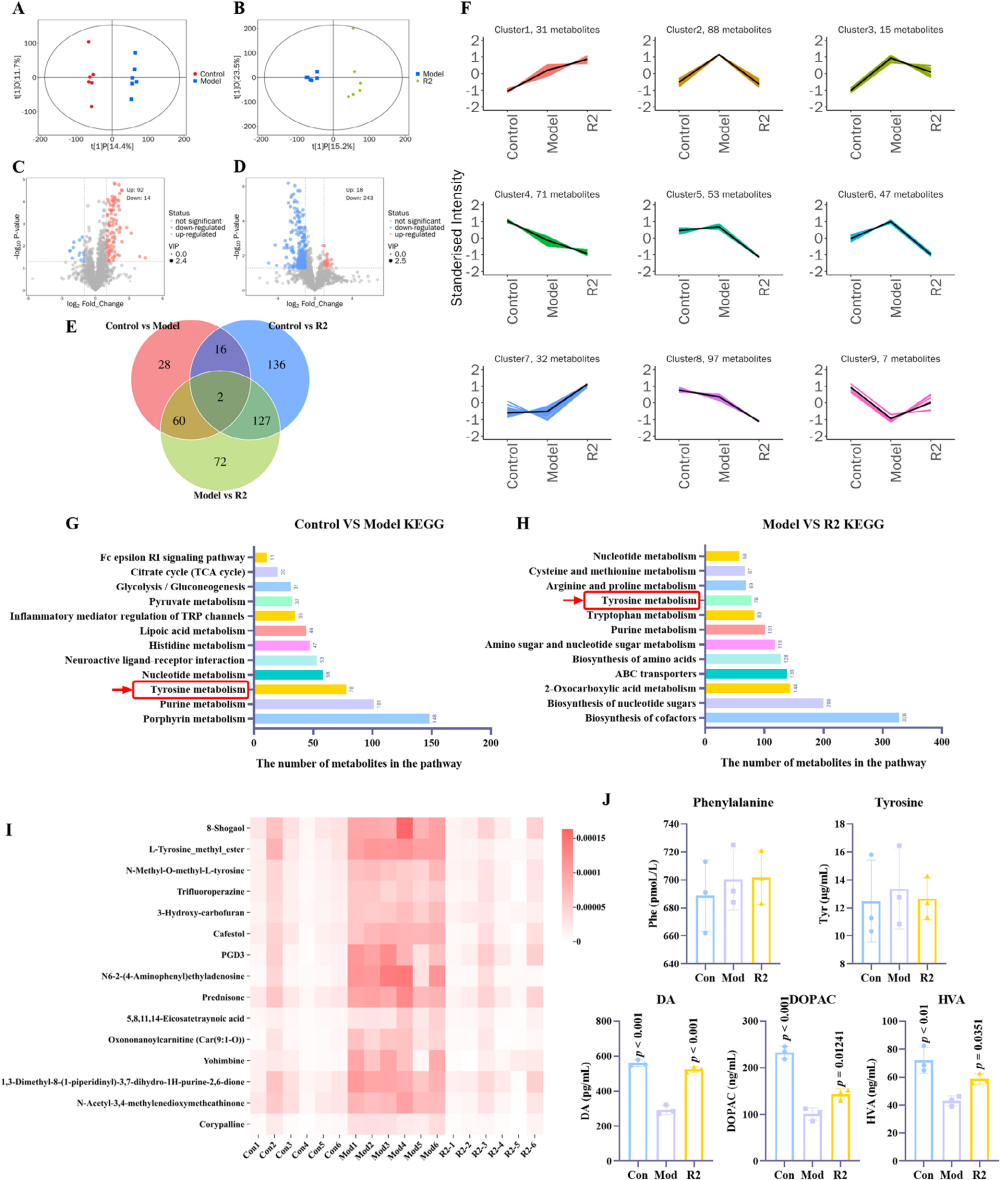
Metabolomics analysis of the metabolic pathways through which R2 exerts pharmacological effects. (A, B) PLS‐DA analysis of Con versus Mod, Mod versus CAG‐H; (C, D) Volcanic diagram of differential metabolites between Con versus Mod (C) and Mod versus CAG‐H (D); (E) Venn analysis of differential metabolites between Con versus Mod versus R2; (F) K‐Means analysis. Standardize the relative content of all differential metabolites identified according to screening criteria in all group comparisons using *z*‐score, and then perform K‐Means clustering analysis; (G, H) KEGG analysis related to differential metabolites; (I) Key differential metabolite heatmap analysis; (J) Detection of key metabolites in the tyrosine metabolism pathway by Elisa kit. (*p* vs. Model group).

The gut‐brain axis is an important direction for exploring the pathogenesis of PD, and various neurotoxic substances may enter the circulation through the disruption of the intestinal barrier, thereby interfering with the central nervous system [30, 31]. In order to comprehensively investigate the effect of compound R2 on MPTP induced mice, we used LC–MS to detect intestinal contents in different groups, analyze differential metabolites and potential metabolic pathways, and then use them for subsequent research. In this study, the intestinal contents of three groups (*n* = 6) of mice were analyzed and differential metabolites were identified. In the pairwise comparison under PLS‐DA analysis, the distinction between different groups is good, indicating significant differences between groups (Figure 5A,B). In the statistical analysis of differential metabolites between different groups, MPTP treatment resulted in upregulation of 92 metabolites and downregulation of 14 metabolites in the model group; after R2 treatment, compared with the model, R2 caused upregulation of 18 metabolites and downregulation of 243 metabolites, indicating that some differential metabolites showed significant regression after R2 treatment (Figure 5C,D). To this end, we used K‐mean clustering analysis to divide differential metabolites into 9 clusters based on different trends, among which, clusters 2, 6 and 7 respectively show that after R2 treatment, some differential metabolites undergo regression and tend towards the control group score (Figure 5F). Finally, the differences in the number of metabolites between different groups were shown through Venn diagrams (Figure 5E).

Furthermore, in KEGG analysis based on differential metabolites, we found that the tyrosine metabolism pathway became a common pathway between the Con versus Mod and Mod versus R2 groups, indicating that the tyrosine metabolism pathway plays an important role in the pharmacological effects of R2 (Figure 5G,H). Amino acid metabolism is closely related to neurodegenerative diseases, among which tyrosine metabolism is particularly important, affecting various aspects such as neurotransmitter regulation, metabolite action, enzyme activity, etc. [32, 33]. Dopamine, as an important product of tyrosine metabolism, affects the activity of microglia and astrocytes through its receptors, inhibiting excessive activation of microglia [34]. TH is the rate‐limiting enzyme for dopamine synthesis, and its decreased activity may lead to neurotransmitter imbalance, indirectly promoting inflammation [35]. In the above assays, it was confirmed that compound R2 can effectively restore the number of TH positive cells and inhibit the activation of microglia. To further verify that compound R2 can regulate the tyrosine metabolic pathway in brain tissue, we detected the contents of key metabolites in brain tissue of each group (Figure 5J). The results showed that there were no significant differences in the contents of phenylalanine and tyrosine (the upstream products of tyrosine metabolism) among different groups, indicating that MPTP induction and R2 intervention exerted little effect on these two amino acids. However, due to the reduction in TH content caused by MPTP, the downstream products of tyrosine metabolism (e.g., dopamine (DA), 3,4‐dihydroxyphenylacetic acid (DOPAC), and homovanillic acid (HVA)) decreased significantly after MPTP induction, whereas their levels were obviously restored following R2 treatment. These experimental data further demonstrate that compound R2 exerts its pharmacological effects by regulating the tyrosine metabolic.

After cross‐analysis of differential metabolites between the Con versus Mod and Mod versus R2 comparison groups, we identified 62 shared differential metabolites. To focus on metabolic disorders highly relevant to PD pathology, we selected “PD” and “tyrosine metabolism” as core keywords based on literature evidence and screened out 15 compounds with the strongest potential associations and clear biological implications for in‐depth analysis. As shown in Figure 5I, these 15 key metabolites exhibited characteristic expression perturbations in the model group, while their levels in the R2 intervention group showed a significant trend of reversal towards the Control. Specifically, these metabolites are mainly classified into the following categories:

(1) Molecules involved in the tyrosine metabolic pathway: Including L‐Tyrosine methyl ester and its derivative N‐methyl‐O‐methyl‐L‐tyrosine. The reversal of these molecules suggests that R2 may exert a regulatory effect on tyrosine metabolic flux or related neurotransmitter synthesis pathways. (2) Lipid mediators and signaling molecules: Changes in prostaglandin PGD3 and 5,8,11,14‐eicosatetraynoic acid—an inhibitor of arachidonic acid metabolism‐indicate that pathways associated with neuroinflammation or oxidative stress may be modulated by R2 intervention. (3) Other potential biomarkers: For example, the reversal of the carnitine derivative Oxononanoylcarnitine (Car (9:1‐O)) and the glucocorticoid Prednisone further reveals the systemic regulatory role of R2 in energy metabolism and stress responses. In summary, literature‐guided screening not only enriched the set of key metabolic targets, but also heatmap analysis intuitively confirmed that R2 intervention can specifically reverse the perturbations of these PD pathology‐related metabolites, providing core metabolomic evidence for elucidating its mechanism of action.

The results showed that after administration of compound R2, there was varying degrees of regression in different metabolites, and it tended towards the control group level. Furthermore, tyrosine metabolism can intervene in norepinephrine levels, thereby regulating the NF‐κB pathway and reducing the production of inflammatory cytokines [36]. Among the 15 key differential metabolites, multiple metabolites are also potentially associated with the NF‐κB signaling pathway. For example, N‐methyl‐O‐methyl‐L‐tyrosine, as a methylated metabolite of tyrosine, has been shown to further exacerbate inflammation levels in LPS induced BV2 models, and can be blocked by the NF‐κB signaling pathway inhibitor Bay11‐7082. Its potential mechanism of action is the aggregation of methylation products, activation of TLR4/ROS, promotion of NF‐κB nuclear translocation, and amplification of inflammatory levels. Meanwhile, in previous studies, we found that CAG can effectively regulate the NF‐κB pathway and alleviate neuroinflammatory levels [15]. Therefore, the analysis results based on metabolomics provide direction for our subsequent research to explore whether the pharmacological effects of compound R2 are related to the NF‐κB pathway?

### 3.7 Compound R2 Regulates the TLR4/NF‐κB Signaling Pathway to Improve Neuroinflammation

**FIGURE 6:**
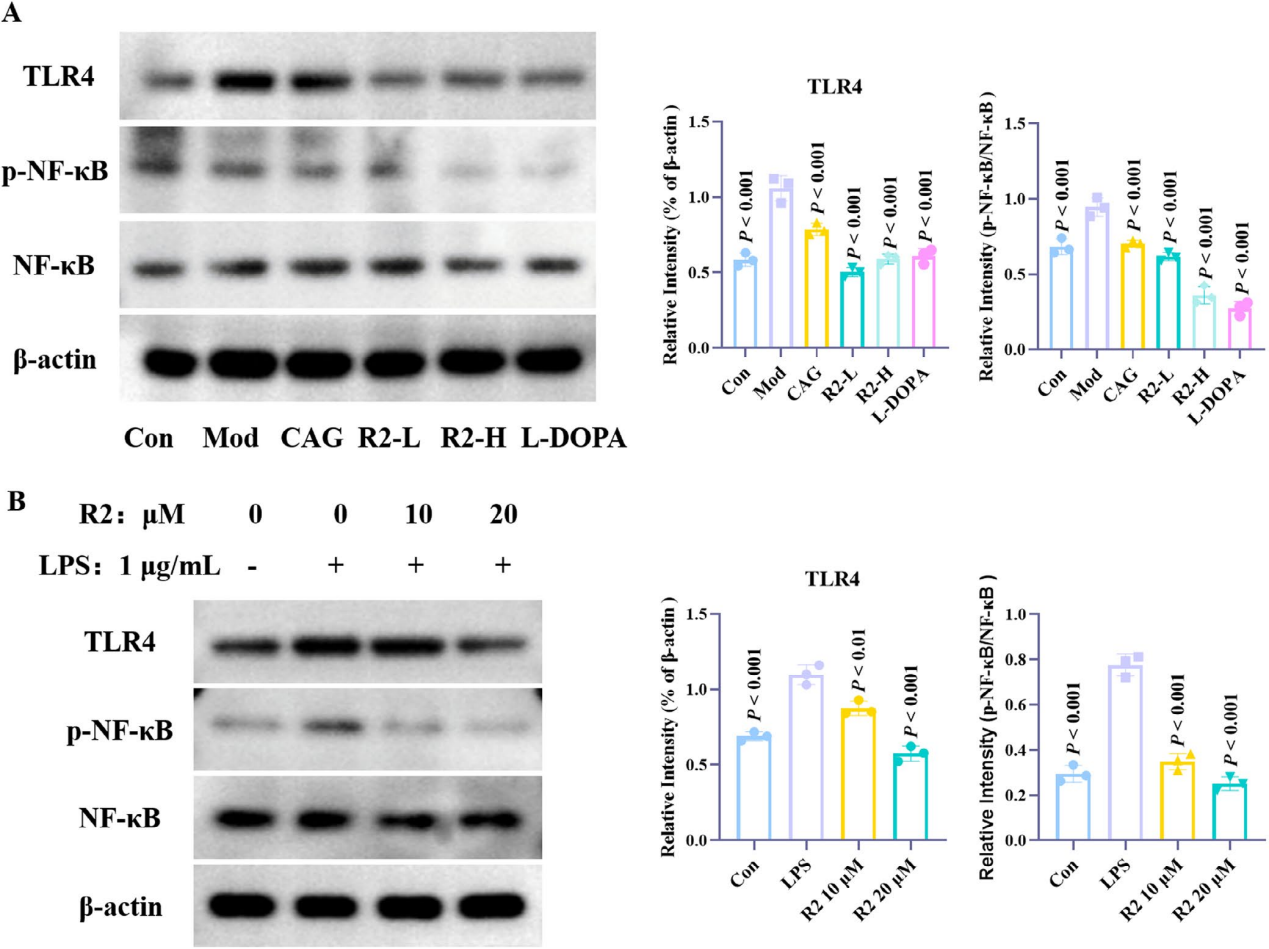
Compound R2 regulates the TLR4/NF‐κB signaling pathway to improve neuroinflammation. (A) MPTP induced PD animal model; (B) LPS induced BV2 neuroinflammatory cell model.

In previous studies, we found that CAG can regulate the TLR4/NF‐κB signaling pathway to inhibit the progression of neuroinflammation. In this study, R2 intervened in the tyrosine metabolism pathway to exert pharmacological effects, and tyrosine metabolism is closely related to the NF‐κB pathway, making NF‐κB a potential signaling pathway. Therefore, we investigated the effect of compound R2 on the TLR4/NF‐κB signaling pathway. The results showed that compound R2 can regulate the expression of TLR4, effectively reduce the phosphorylation level of NF‐κB, and inhibit the classical inflammatory activation pathway of NF‐κB (Figure 6A). In addition, in the cell model, we obtained the same results as the tissue samples (Figure 6B). Therefore, we conclude that compound R2 improves neuroinflammatory levels by regulating the TLR4/NF‐κB signaling pathway.

### 3.8 Compound R2 Improves Inflammaging of Microglia Cells

PD is a disease with obvious age‐related characteristics, and aging is the core risk factor for its occurrence [37]. It is driven by multiple molecular mechanisms to promote disease progression, and inflammageing is currently a hot research direction in neurodegenerative diseases [38, 39]. CAG is a natural source telomerase activator that has been reported to effectively enhance telomerase activity [40]. Meanwhile, we have previously demonstrated that CAG is an effective neuroinflammatory inhibitor. Therefore, exploring new natural products around CAG has become a potential way to break through inflammageing. In this study, compound R2 showed good neuroinflammatory inhibitory effects. In metabolomics analysis, key amino acid metabolites such as N‐methyl‐O‐methyl‐L‐tyrosine can interfere with telomerase activity and regulate inflammageing through oxidative stress, epigenetic regulation, and NF‐κB signaling. Therefore, we further investigated the effect of R2 on telomerase activity.

**FIGURE 7:**
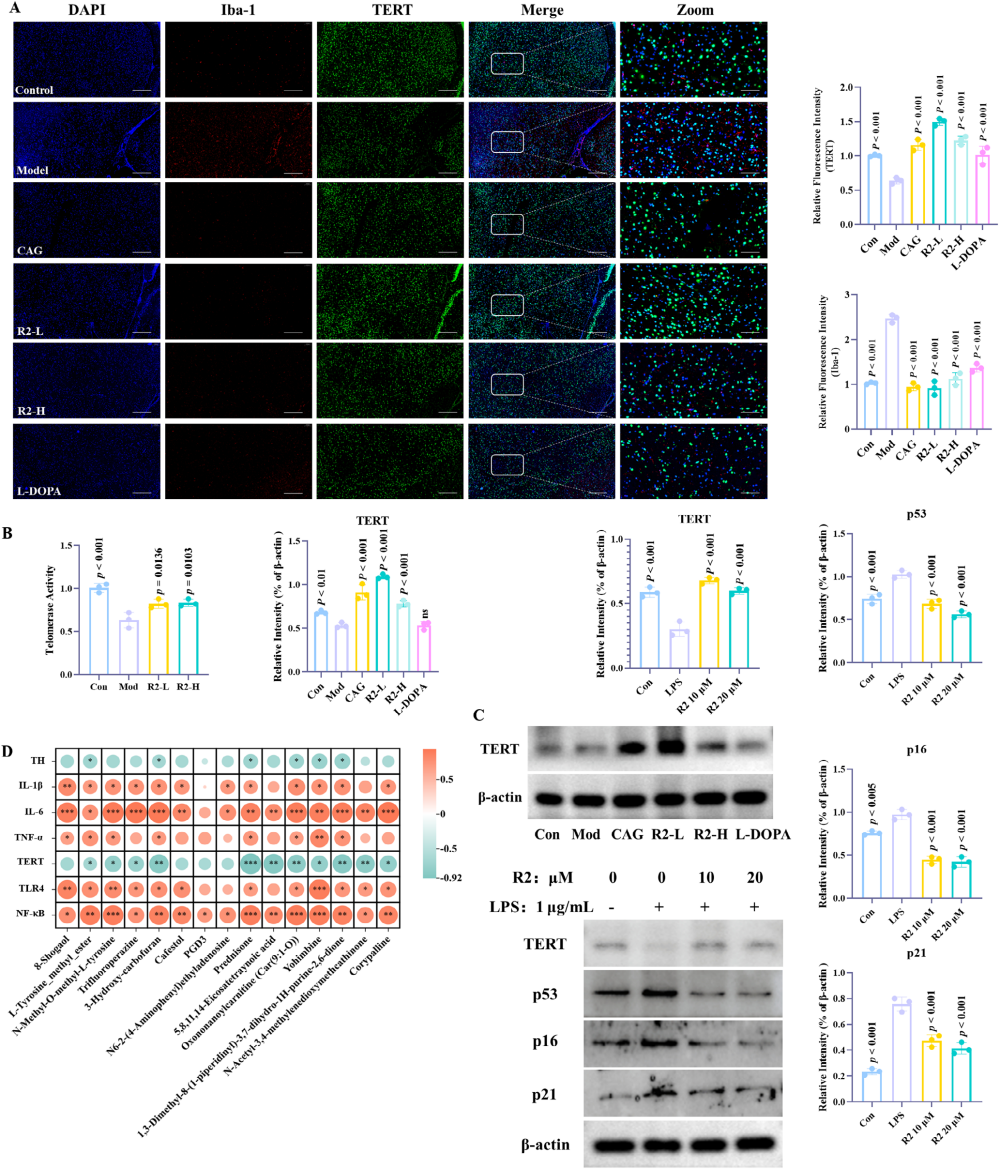
Compound R2 enhances telomerase activity and improves aging. (A) Immunofluorescence analysis of Iba‐1 and TERT expression in microglia in the substantia nigra of the brain. (The scale of DAPI, TERT, Iba‐1 and Merge is 100 μm; The scale of ZOOM is 50 μm). (B) Compound R2 enhances telomerase activity in PD model. (C) Compound R2 regulates TERT and aging related protein expression in PD and cell models. (*p* vs. Model group); (D) Correlation analysis between key differential metabolites and inflammatory indicators.

The TERT gene is the core component of telomerase and encodes the catalytic subunit of telomerase [41]. The expression of TERT is consistent with the expression of telomerase activity and closely related to the degree of telomerase activation [42]. Therefore, we analyzed the expression of TERT and Iba‐1 through immunofluorescence. The results showed that after MPTP induction, the expression of TERT in microglia was significantly reduced, while the expression of its activation marker Iba‐1 was significantly increased. MPTP can promote the process of inflammageing and increase the levels of inflammation and aging. After treatment with compound R2, the characteristics of inflammageing were significantly improved, the expression of TERT was promoted, and the activation of microglia was inhibited (Figure 7A).

Similarly, we detected the expression of TERT in brain tissue and cell models using Western blot and obtained the same results (Figure 7C). In addition, we used the TRAP method to study telomerase activity in PD models, and the results showed that compound R2 can effectively enhance the telomerase activity decline induced by MPTP. (Figure 7B). In addition, in order to better demonstrate the cellular aging status induced by LPS in BV2 cells, we detected key indicators related to cellular aging, including p53, p21, and p16. In the model group, there were varying degrees of improvement, and after treatment with compound R2, there was a significant improvement. So, in the neuroinflammatory model, LPS can exacerbate the aging state of cells, inhibit telomerase activity, and compound R2 can effectively reverse the inflammatory process.

To further verify the association between the inflammatory pathway (TLR4/NF‐κB) and TERT, as well as to explore in depth the regulatory mechanism of compound R2 on inflammation and aging, we employed the NF‐κB agonist (RapRIV) to evaluate the correlation between compound R2 and the signaling pathway. The results showed that compound R2 could effectively inhibit the inflammation and the decrease in TERT expression induced by LPS; the intervention of RapRIV reversed the pharmacological effects of R2 (Figure S90). These findings indicate that compound R2 can effectively inhibit the NF‐κB signaling pathway, thereby verifying its regulatory role in the TLR4/NF‐κB/TERT axis.

Finally, we comprehensively analyzed the above experimental data and observed the correlation between 15 key differential metabolites and inflammatory indicators through Pearson correlation analysis (Figure 7D). The results showed that 15 differential metabolites were not associated with inflammatory indicators to varying degrees and had statistical significance, especially metabolites directly or indirectly related to tyrosine, such as N‐Methyl‐O‐methyl‐L‐tyrosine. Ultimately, we concluded that compound R2 can exert a neuroinflammatory regulatory effect by modulating key metabolites in the tyrosine metabolism pathway, thereby improving the symptoms associated with PD.

## 4 Discussion

Over the years, due to the diverse pathogenesis of PD, combined with the influences of genetics, environment and other factors, research on PD has emerged in an endless stream. A variety of new drugs have been continuously developed for clinical treatment based on the multiple pathogenesis mechanisms of PD, but they have never been able to fundamentally curb the progressive loss of neurons [43]. Therefore, identifying the common issues among the etiologies has become a focus, and neuroinflammation is a common problem across multiple etiologies. Meanwhile, as a disease with obvious age‐related characteristics, aging has become a key factor in the onset of PD. Inflammation and aging interact with and interfere with each other, continuously leading to the death of neuronal cells [44]. The concept of “inflammaging” has also been proposed and increasingly applied in the research of various diseases [45]. Thus, breaking the vicious cycle between inflammation and aging is a potential research direction for drug intervention in PD.

CAG is an effective ingredient in *Astragalus membranaceus*, which is well known by researchers in antiaging small molecule activity screening. It is currently a reported natural activator of telomerase and has been applied as a marketed product in dietary supplements or skincare products [46]. In recent years, research on CAG has mainly focused on its antiaging, anti‐inflammatory, and neuroprotective effects. In our previous studies, we found that CAG can regulate the TLR4/NF‐κB signaling pathway to inhibit neuroinflammation in PD models [15]. However, a large number of in vitro and in vivo experiments have shown that there is significant room for improvement in the anti‐inflammatory activity of CAG. Therefore, we aim to enhance its pharmacological efficacy through structural optimization. Small‐molecule carboxylic acids are commonly used structural motifs in organic synthesis, as they can effectively improve the pharmacological effects and physicochemical properties (e.g., water solubility) of compounds [47]. Initially, we introduced amino acids and benzoic acid into the CAG structure, but the results of activity evaluation were not satisfactory. Subsequently, we made further attempts based on the previous work: for example, introducing hydroxyl groups into the benzene ring of benzoic acid to form salicylic acid, and modifying the structure via “Alkene modification” to form cinnamic acid (α, β‐unsaturated carbonyl group) [48]. Eventually, among the cinnamic acid series of compounds, we obtained compound R2, which significantly enhanced anti‐inflammatory activity. This indicates that the α, β‐unsaturated carbonyl structure plays a crucial role in improving the activity of CAG.

To better investigate the mechanism by which compound R2 exerts its pharmacological effects in PD models, we employed metabolomics to analyze changes in metabolites before and after treatment with compound R2. Through KEGG analysis, we identified “tyrosine metabolism” as a common pathway between the Con versus Mod and Mod versus R2 comparison groups. Tyrosine metabolism plays a crucial role in PD and neuroinflammation. The synthesis of dopamine (DA) is one of the core pathways of tyrosine metabolism, and changes in DA directly affect the symptoms of PD. The reduction in the number of TH‐positive cells caused by MPTP can limit DA synthesis, thereby inducing neuronal apoptosis [49]. Neuroinflammation, particularly the sustained activation of microglia, is a common feature of PD and many other neurological diseases. Tyrosine metabolites act as “signaling molecules” and “amplifiers” in this process. DA or its oxidative metabolites (such as quinones and 3,4‐dihydroxyphenylacetaldehyde, DOPAL) can be recognized by Toll‐like receptors (TLRs) on the surface of microglia, triggering their activation, initiating inflammatory signaling pathways such as NF‐κB, releasing inflammatory factors, and amplifying the inflammatory response [50]. Therefore, through the production of neurotoxic substances (e.g., DOPAL) and inflammatory signaling molecules (e.g., oxidized DA), tyrosine metabolism directly leads to the death of dopaminergic neurons and strongly activates microglia. In turn, sustained neuroinflammation further inhibits TH activity, disrupts normal tyrosine metabolism, and accelerates neuronal loss by generating ROS and inflammatory factors [51]. These two processes form a mutually reinforcing and progressively amplifying vicious cycle, which together drives the occurrence and progression of PD.

**FIGURE 8:**
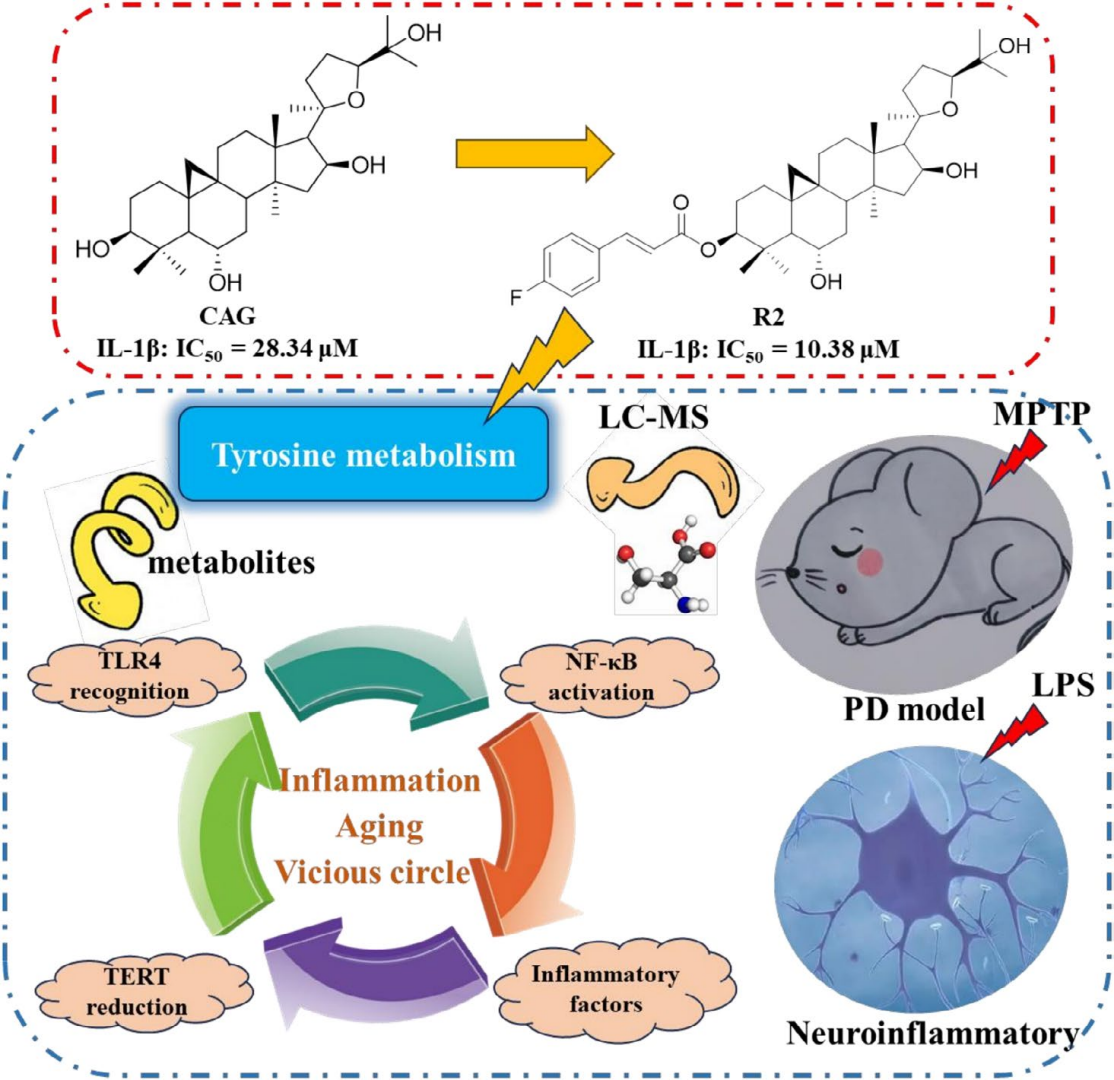
Schematic diagram of the mechanism by which compound R2 exerts its pharmacological effects.

In the analysis of differential metabolites, N‐Methyl‐O‐methyl‐L‐tyrosine (a tyrosine metabolite) emerged as the focus of the study. Compound R2 effectively ameliorated the abnormal accumulation of N‐Methyl‐O‐methyl‐L‐tyrosine. In the neuroinflammation model, there is a potential association between N‐Methyl‐O‐methyl‐L‐tyrosine and TERT activity, which is primarily mediated through oxidative stress, epigenetic regulation, and NF‐κB signaling [52]. The accumulation of this metabolite can induce the release of mitochondrial reactive oxygen species, activate p53, and subsequently enable p53 to bind to the TERT promoter. This binding leads to epigenetic silencing, resulting in decreased TERT transcription levels and reduced activity [53]. Cellular senescence further elevates the inflammatory response, thereby forming a vicious cycle. At the onset of this research project, we aimed to identify the key link between inflammation and senescence. The regulation of N‐Methyl‐O‐methyl‐L‐tyrosine by compound R2 may serve as a breakthrough point to break the cycle of “inflammaging”. Therefore, we investigated the regulatory effect of compound R2 on the TLR4/NF‐κB/TERT pathway. The results showed that compound R2 can effectively inhibit the signal activation of TLR4 and suppress the nuclear translocation of NF‐κB, thereby improving TERT activity (Figure 8).

In summary, this study designed and synthesized 29 carboxylic acid derivatives of CAG. Evaluation using in vitro PD models and neuroinflammation models revealed that compound R2 exhibited the optimal effects in enhancing cell viability and inhibiting neuroinflammation. In the MPTP‐induced PD mouse model, compound R2 effectively improved the behavioral and pathological indicators of PD, such as alleviating MPTP‐induced bradykinesia and restored the number of TH‐positive cells. In the analysis of neuroinflammatory levels, compound R2 significantly reduced the release of inflammatory factors and attenuated neuroinflammation by inhibiting microglial activation. Furthermore, we analyzed the abnormally differential metabolites in intestinal contents after R2 treatment. The KEGG analysis indicated that compound R2 ameliorated MPTP‐induced neuroinflammation through the tyrosine metabolism pathway and suppressed the abnormal accumulation of the metabolite N‐Methyl‐O‐methyl‐L‐tyrosine, which was potentially associated with NF‐κB activation and TERT expression. Subsequent experiments further validated this conclusion. Ultimately, using both in vitro and in vivo models, we confirmed that compound R2 can regulate tyrosine metabolism imbalance, improve the TLR4/NF‐κB/TERT signaling pathway, and break the vicious cycle between inflammation and aging, and alleviates MPTP‐induced PD symptoms, thereby providing a scientific basis for the drug development of CAG and PD therapeutic agents.

## Author Contributions

S.X.: methodology, data analysis, writing – original draft, project administration, funding acquisition; L.L.: methodology, validation, software, data analysis; X.Q: Writing – review and editing; L.X.: supervision, project administration; Z.L.: conceptualization, methodology, funding acquisition; Z.C.: conceptualization, resources, supervision, project administration. All authors have approved the present version of the manuscript and have agreed to be accountable for all aspects of the work regarding questions related to the accuracy or integrity of any part of the work.

## Funding

The work was supported by Fundamental Research Program in Shanxi Province (202303021222198), Scientific Research Fund for the Doctoral Young Scholars, SXTCM (2023BK05), Xinglin Talents Program, SXTCM (2025XK15), Excellent Doctoral Graduates of Shanxi University of Chinese Medicine Research Launch Fund Project (2023BKS14), Key Laboratory of Traditional Chinese Medicine Lifeomics and Innovative Drug Research and Development (zyyyjs2024019), Secondary Discipline Construction Project of Chinese Materia Medica Analysis (2025XK39).

## Ethics Statement

All experimental procedures involving the mice were reviewed and approved by the Animal Experiment Ethics Committee of Shanxi University of Chinese Medicine, with the approval number AWE202307367.

## Consent

The authors have nothing to report.

## Conflicts of Interest

The authors declare no conflicts of interest.

## Supporting information


**Data S1:** cns70787‐sup‐0001‐supinfo.doc.

## Data Availability

All data supporting the findings of this study are available within the article from the corresponding author upon reasonable request.
