# Supplementary material for: Cycloastragenol Derivatives Improve Tyrosine Metabolism, Regulate TLR4/NF‐κB/TERT Signaling Pathways, and Inhibit MPTP Induced Neuroinflammation and PD Symptoms
Source: CNS Neurosci Ther. 2026 Feb 11;32(2):e70787. doi: 10.1002/cns.70787 (PMC12892403; doi:10.1002/cns.70787)
Supplement: Supplementary file 1 — Data S1: cns70787‐sup‐0001‐supinfo.doc. [file CNS-32-e70787-s001.doc]

Cycloastragenol derivatives improve tyrosine metabolism, regulate TLR4/NF-κB/TERT signaling pathways, and inhibit MPTP induced neuroinflammation and PD symptoms.

Shengnan Xiao^1^, Lianmei Liu^1^, Xuemei Qin^1,2^, Lei Xu^1*^, Zhenyu Li^1,2*^, Zhi Chai^1*^.

*^1^ Institute of Taihang Materia Medica, Shanxi University of Chinese Medicine, Jinzhong, 030600, China.*

*^2^* *Modern Research Center for Traditional Chinese Medicine of Shanxi University, NO.92, Wucheng Road, Taiyuan 030006, China;*

*^*^ Corresponding author: E-mail address:* Lei Xu *(*[*18246691730@163.com*](mailto:18246691730@163.com)*);* Zhenyu Li ([*lizhenyu@sxu.edu.cn*](mailto:lizhenyu@sxu.edu.cn))*;* Zhi Chai *(*[*chaizhi008@126.com)*](mailto:chaizhi008@126.com))

**Section 1:** Structural characterization data and original graphs of all 29 compounds

**Section 2:** Supplementary data for pharmacological experiments of compound **R2**

**Section 1**

Nuclear Magnetic Resonance (NMR) spectra were recorded on a Bruker 600 MHz AV-NEO spectrometer (Bruker Corporation, Karlsruhe, Germany) in deuterated chloroform (CDCl₃) solution, with tetramethylsilane (TMS) as the internal standard. High-resolution mass spectrometry (HRMS) data for the target compounds were acquired using a Waters G2-S quadrupole time-of-flight (QTOF) mass spectrometer. Melting points were determined on an uncorrected X-4 digital micro melting point apparatus.

### Compound A1

White powder, melting point: 236-238℃. ^1^H-NMR (CDCl_3_, 600 MHz, ppm): δ 0.40(d, *J* = 4.2 Hz, 1H, H-19b), 0.54(d, *J* = 4.2 Hz, 1H, H-19a), 0.95, 1.05, 1.11, 1.13, 1.21, 1.25, 1.29(s, 3H, -CH_3_×7), 1.44(s, 12H, -CH_3_×4), 2.34(d, *J* = 7.8 Hz, 1H), 2.60(q, *J* = 10.2 Hz, 1H), 3.52(t, *J_1_* = 8.4 Hz, *J_2_* = 11.4 Hz, 1H, H-6), 3.77(t, *J_1_* = 7.2 Hz, *J_2_* = 14.4 Hz, 1H, H-24), 4.30(t, *J_1_* = 7.2 Hz, *J_2_* = 13.8 Hz, 1H), 4.63(dd, *J_1_* = 4.2 Hz, *J_2_* = 10.8 Hz, 1H, H-3), 4.70(q, *J* = 7.8 Hz, 1H, H-16), 5.09(d, *J* = 7.2 Hz, 1H); ^13^C-NMR (CDCl_3_, 150 MHz, ppm): δ 173.17(-C=O), 155.23(-OCO-NH-), 87.17(C-20), 81.42(C-24), 81.48(C-3), 79.84(-C(CH_3_)_3_), 73.46(C-16), 71.95(C-25), 68.91(C-6), 57.60(C-17), 53.80(C-5), 49.68(-CH-NH), 47.34(C-8), 46.65(C-14), 46.08(C-15), 45.06(C-13), 40.69(C-4), 38.18(C-7), 34.54(C-22), 33.01(C-1), 31.79(C-12), 31.66(C-19), 29.41(C-2), 28.49(-C(CH_3_)_3_), 28.30(C-10), 28.00(C-28), 27.81(C-21), 26.90(C-11), 26.61(C-27), 26.01(C-26), 25.86(C-23), 21.61(C-9), 20.93(C-18), 20.19(C-30), 19.07(-CH-CH_3_), 16.54(C-29); ESI-HRMS (m/z): calcd for C_38_H_63_O_8_NNa^+^ [M+Na]^+^: 684.4451, found: 684.4451.


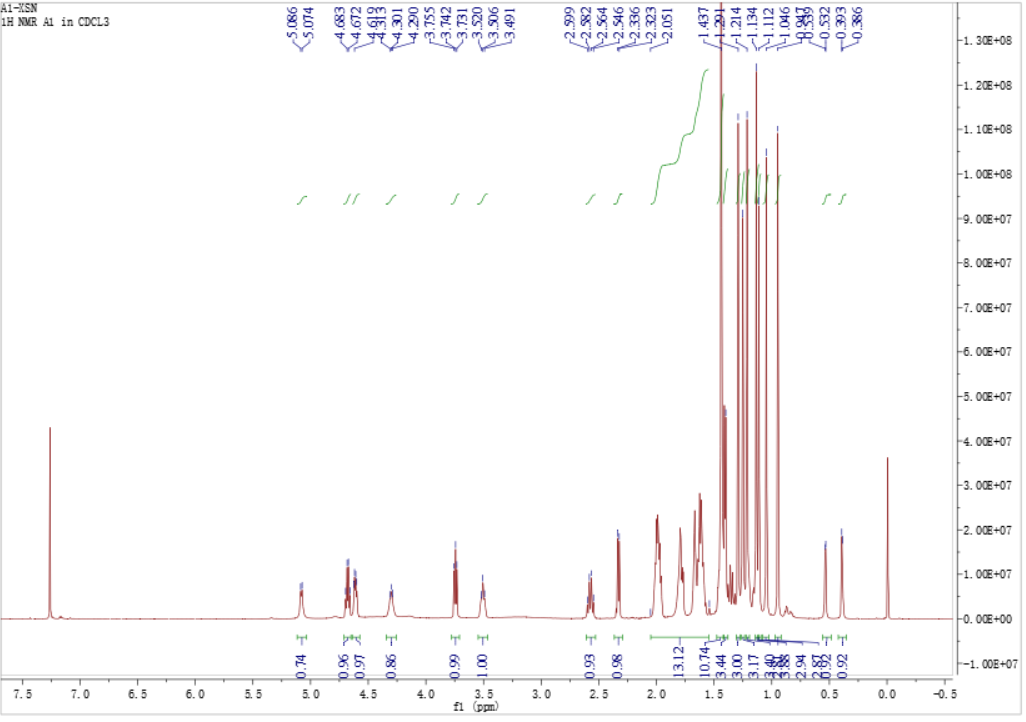


Figure.S1. ^1^H-NMR of compound A1


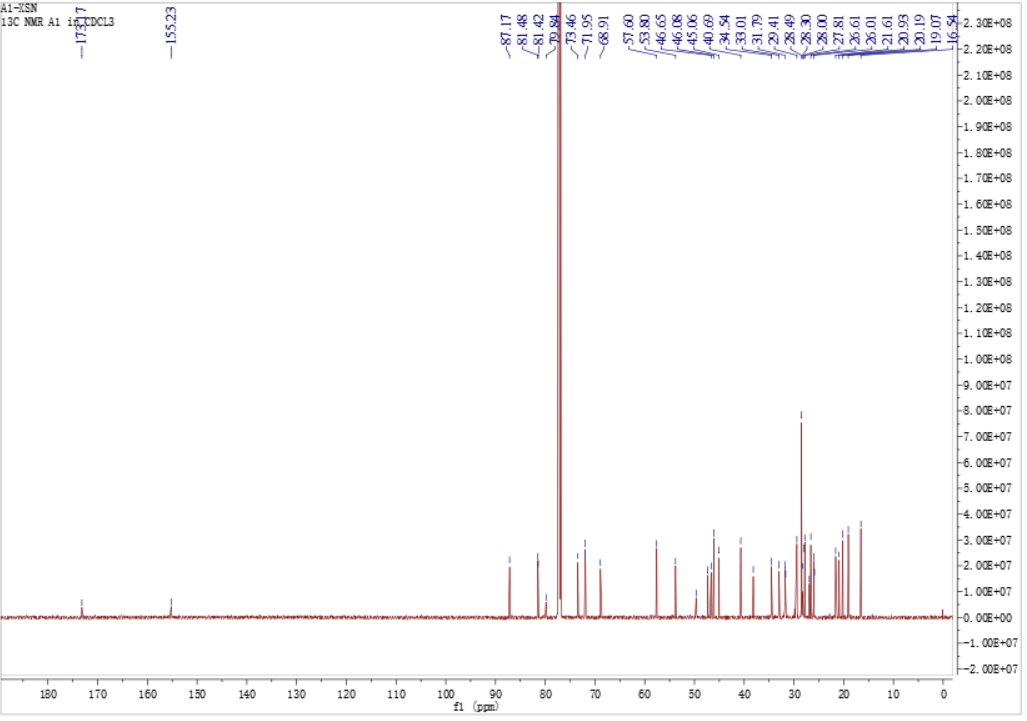


Figure.S2. ^13^C-NMR of compound A1


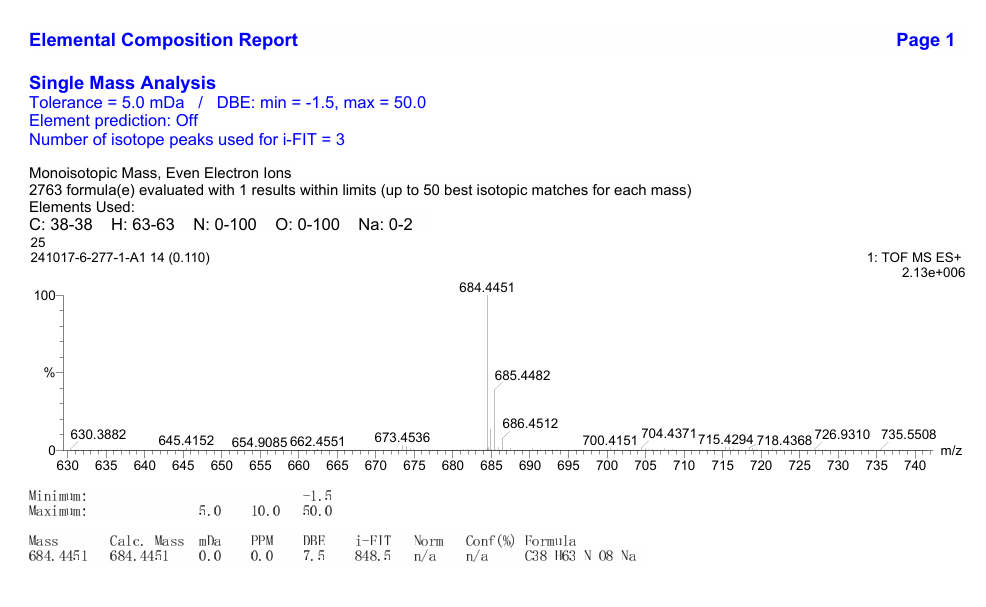


Figure.S3. HRMS of compound A1

### Compound A2

White powder, melting point: 242-244℃. ^1^H-NMR (CDCl_3_, 600 MHz, ppm): δ 0.37(d, *J* = 4.2 Hz, 1H, H-19b), 0.52(d, *J* = 3.6 Hz, 1H, H-19a), 0.95, 1.00, 1.06, 1.13, 1.21, 1.25, 1.29(s, 3H, -CH_3_×7), 1.39(s, 9H, -CH_3_×3), 2.34(d, *J* = 7.8 Hz, 1H), 2.60(q, *J* = 10.2 Hz, 1H), 3.00-3.16(m, 2H), 3.51(t, *J_1_* = 3.0 Hz, *J_2_* = 9.6 Hz, 1H, H-6), 3.76(t, *J_1_* = 6.0 Hz, *J_2_* = 14.4 Hz, 1H, H-24), 4.55-4.59(m, 2H), 4.70(q, *J* = 7.8 Hz, 1H, H-16), 4.90(d, *J* = 8.4 Hz, 1H), 7.17(d, *J* = 8.4 Hz, 2H, AR-H), 7.21-7.23(m, 1H, AR-H), 7.28(t, *J_1_* = 7.2 Hz, *J_2_* = 14.4 Hz, 2H, AR-H); ^13^C-NMR (CDCl_3_, 150 MHz, ppm): δ 173.17(-C=O), 155.22(-CO-NH-), 136.20, 129.51, 128.65, 127.08(AR-C), 87.15(C-20), 81.46(C-24), 81.92(C-3), 79.93(-C(CH_3_)_3_), 73.45(C-16), 71.94(C-25), 68.85(C-6), 57.59(C-17), 54.74(-CH_2_-CH-NH-), 53.76(C-5), 47.31(C-8), 46.64(C-14), 46.07(C-15), 45.03(C-13), 40.54(C-4), 38.54(-C_6_H_5_-CH_2_-CH-), 38.12(C-7), 34.54(C-22), 33.00(C-1), 31.77(C-12), 31.63(C-19), 29.83(C-2), 29.35(C-10), 28.44(-C(CH_3_)_3_), 28.02(C-28), 27.80(C-21), 26.76(C-11), 26.60(C-27), 25.99(C-26), 25.89(C-23), 21.59(C-9), 20.91(C-18), 20.19(C-30), 16.56(C-29); ESI-HRMS (m/z): calcd for C_44_H_67_O_8_NNa^+^ [M+Na]^+^: 760.4764, found: 760.4761.


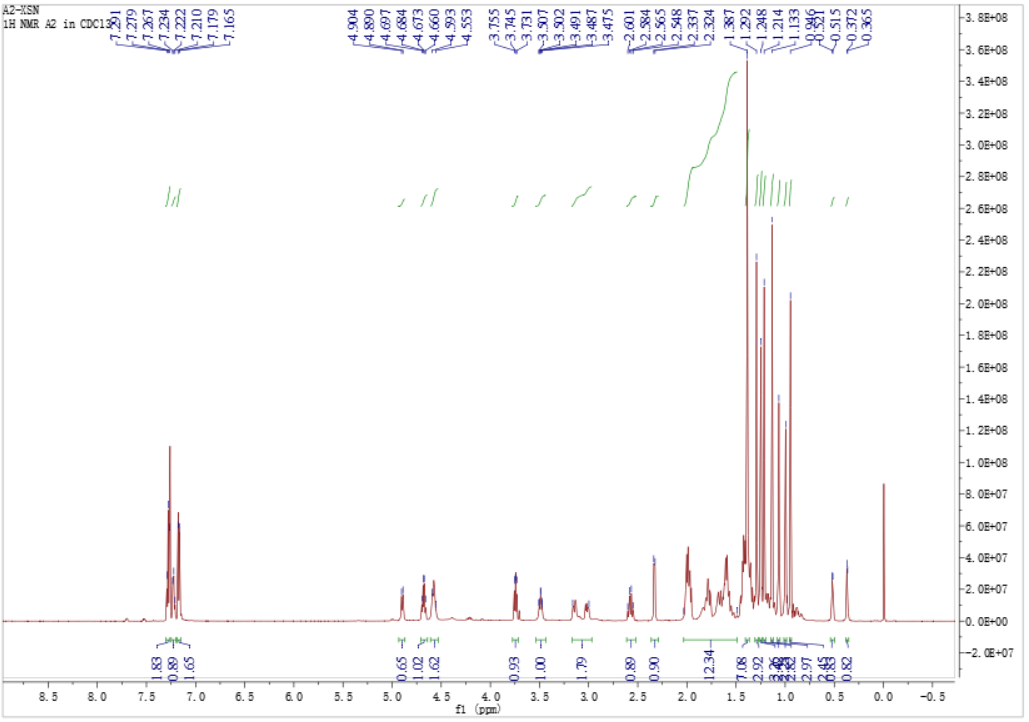


Figure.S4. ^1^H-NMR of compound A2


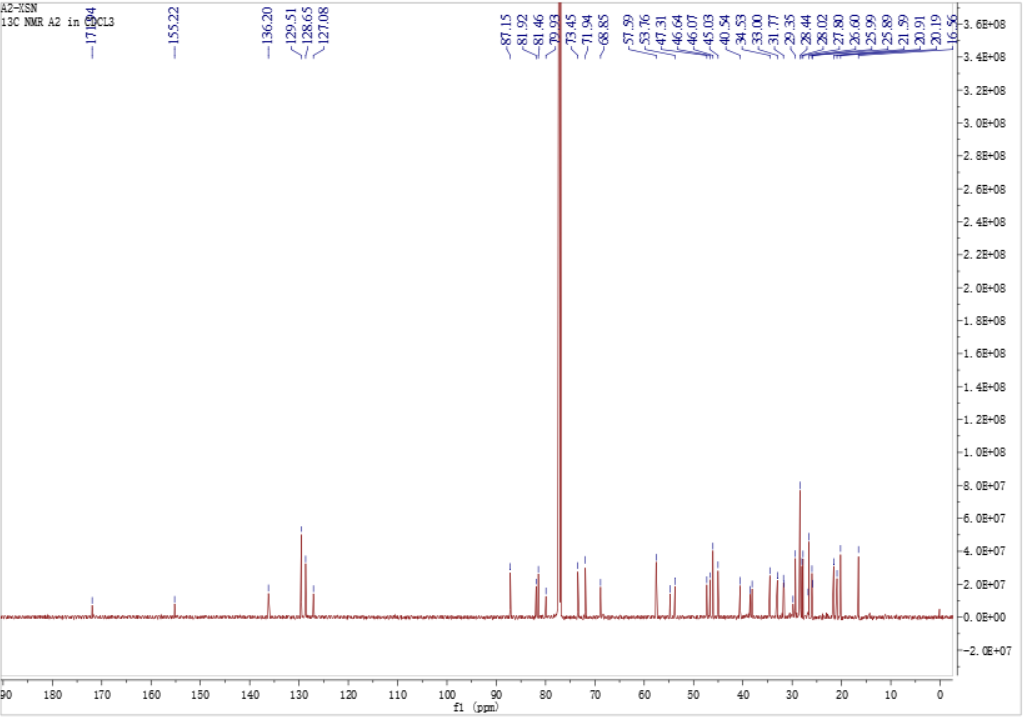


Figure.S5. ^13^C-NMR of compound A2


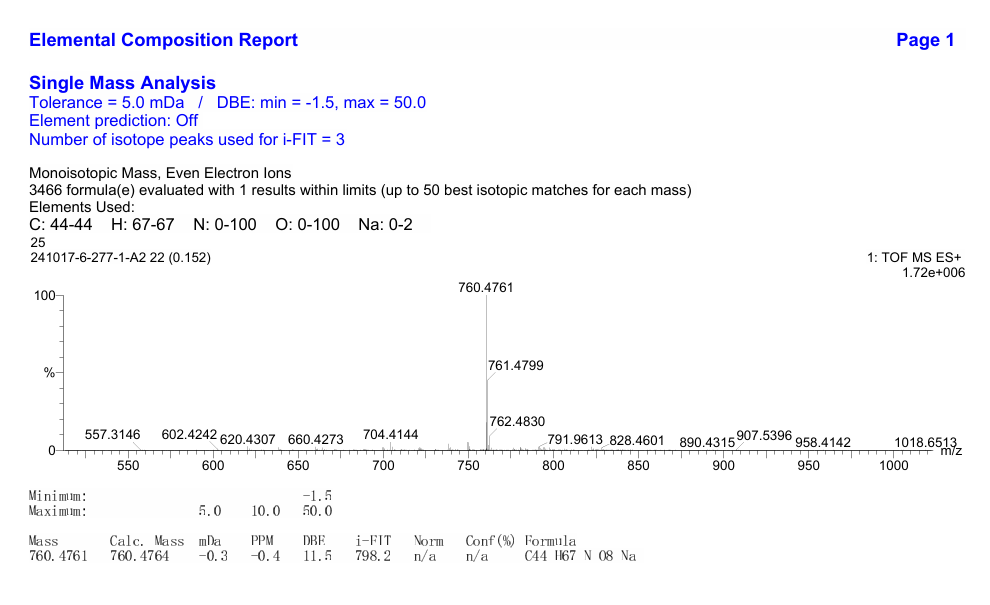


Figure.S6. HRMS of compound A2

### Compound A3

White powder, melting point: 231-233℃. ^1^H-NMR (CDCl_3_, 600 MHz, ppm): δ 0.39(d, *J* = 4.8 Hz, 1H, H-19b), 0.54(d, *J* = 4.2 Hz, 1H, H-19a), 0.95, 1.04, 1.12, 1.14, 1.23, 1.26, 1.30(s, 3H, -CH_3_×7), 1.45(s, 9H, -CH_3_×3), 2.33(d, *J* = 7.8 Hz, 1H), 2.59(q, *J* = 10.2 Hz, 1H), 3.53(t, *J_1_* = 8.4 Hz, *J_2_* = 17.4 Hz, 1H, H-6), 3.76(t, *J_1_* = 7.2 Hz, *J_2_* = 14.4 Hz, 1H, H-24), 3.87-3.95(m, 2H, -NH-CH_2_-CO-), 4.63-4.70(m, 2H, H-16 and H-3), 5.00(s, 1H); ^13^C-NMR (CDCl_3_, 150 MHz, ppm): δ 170.26(-C=O), 155.80(-CO-NH-), 87.20(C-20), 81.51(C-24), 81.65(C-3), 80.05(-C(CH_3_)_3_), 73.47(C-16), 71.96(C-25), 68.93(C-6), 57.59(C-17), 53.85(C-5), 47.22(C-8), 46.59(C-14), 46.09(C-15), 45.11(C-13), 42.78(-NH-CH_2_-CO), 40.62(C-4), 38.12(C-7), 34.54(C-22), 33.05(C-1), 31.80(C-12), 31.58(C-19), 29.84(C-2), 29.35(C-10), 28.47(-C(CH_3_)_3_), 28.32(C-28), 27.85(C-21), 26.88(C-11), 26.62(C-27), 26.00(C-26), 25.77(C-23), 21.61(C-9), 20.95(C-18), 20.18(C-30), 16.47(C-29); ESI-HRMS (m/z): calcd for C_37_H_61_O_8_NNa^+^ [M+Na]^+^: 670.4295, found: 670.4297.


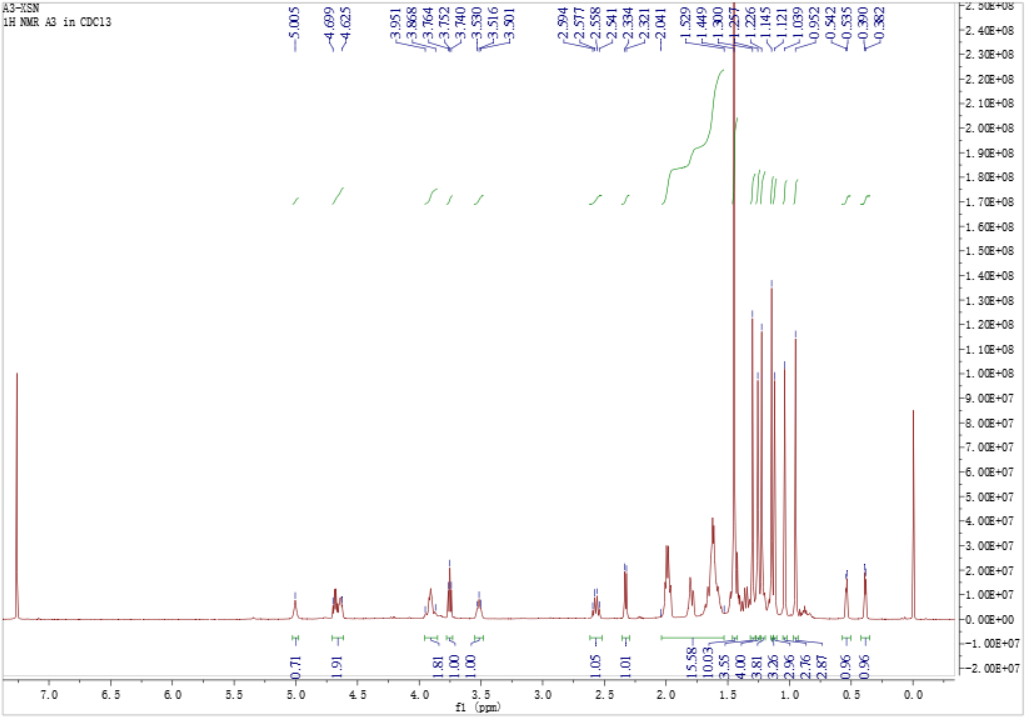


Figure.S7. ^1^H-NMR of compound A3


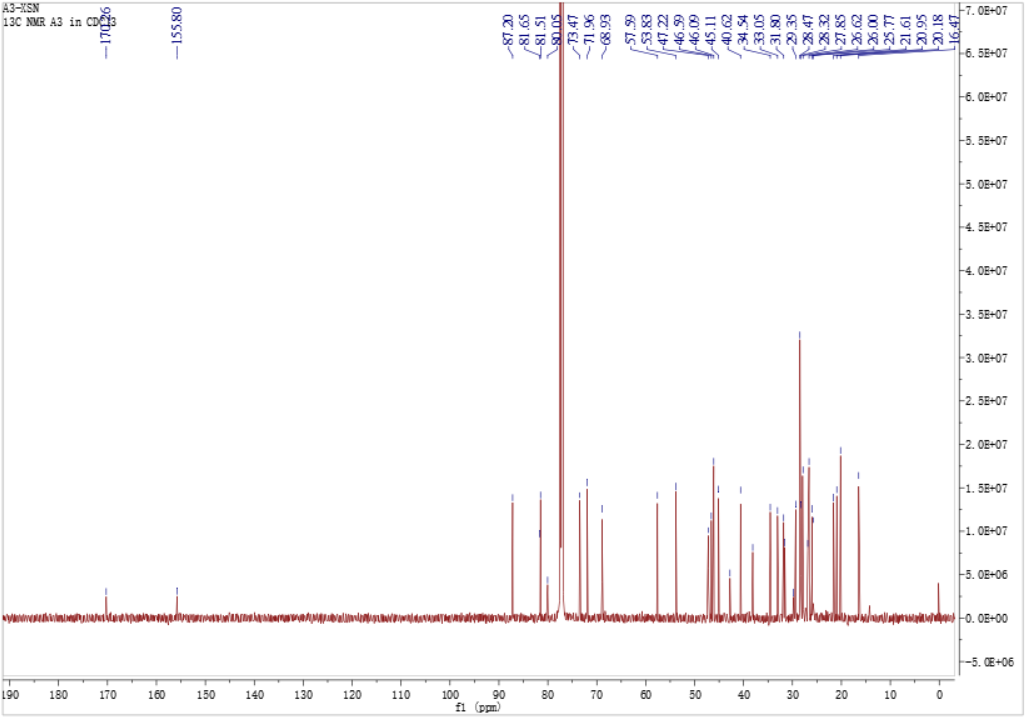


Figure.S8. ^13^C-NMR of compound A3


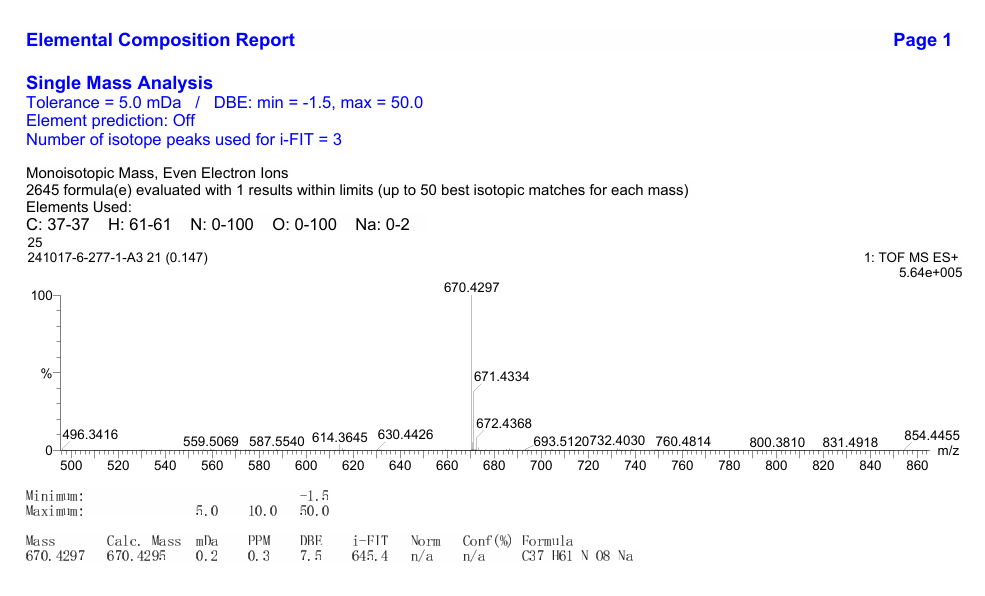


Figure.S9. HRMS of compound A3

### Compound A4

White powder, melting point: 235-237℃.^1^H-NMR (CDCl_3_, 600 MHz, ppm): δ 0.39(d, *J* = 4.8 Hz, 1H, H-19b), 0.53(d, *J* = 3.6 Hz, 1H, H-19a), 0.87(d, *J* = 6.6 Hz, 3H, CH_3_-CH-CH_3_), 0.99(d, *J* = 6.6 Hz, 3H, CH_3_-CH-CH_3_), 0.94, 1.04, 1.20, 1.24, 1.28(s, 3H, -CH_3_×5), 1.12(s, 3H, -CH_3_×2), 1.45(s, 9H, -CH_3_×3), 1.43(s, 9H, -C(CH_3_)_3_), 2.33(d, *J* = 7.8 Hz, 1H), 2.60(q, *J* = 10.2 Hz, 1H), 3.51(t, *J_1_* = 8.4 Hz, *J_2_* = 16.8 Hz, 1H, H-6), 3.74(t, *J_1_* = 8.4 Hz, *J_2_* = 13.8 Hz, 1H, H-24), 4.23(dd, *J_1_* = 4.2 Hz, *J_2_* = 9.6 Hz, 1H, -NH-CH-CO-), 4.61(dd, *J_1_* = 5.4 Hz, *J_2_* = 10.8 Hz, 1H, H-3), 4.69(q, *J* = 7.8 Hz, 1H, H-16), 5.01(d, *J* = 9.6 Hz, 1H); ^13^C-NMR (CDCl_3_, 150 MHz, ppm): δ 172.24(-C=O), 155.92(-CO-NH-), 87.13(C-20), 81.43(C-24), 81.69(C-3), 79.78(-C(CH_3_)_3_), 73.45(C-16), 71.94(C-25), 68.83(C-6), 59.98(-NH-CH-CO-), 57.59(C-17), 53.75(C-5), 47.39(C-8), 46.67(C-14), 46.05(C-15), 44.99(C-13), 40.53(C-4), 38.15(C-7), 34.52(C-22), 32.96(C-1), 31.79(C-12), 31.70(C-19), 31.25(C-2), 29.41(C-10), 28.47(-C(CH_3_)_3_), 28.40(C-28), 28.06(CH_3_-CH-CH_3_), 27.75(C-21), 26.98(C-11), 26.57(C-27), 26.01(C-26), 25.94(C-23), 21.58(C-9), 20.88(C-18), 20.19(C-30), 19.52((CH_3_)_2_CH-), 16.63(C-29); ESI-HRMS (m/z): calcd for C_40_H_67_O_8_NNa^+^ [M+Na]^+^: 712.4764, found: 712.4765.


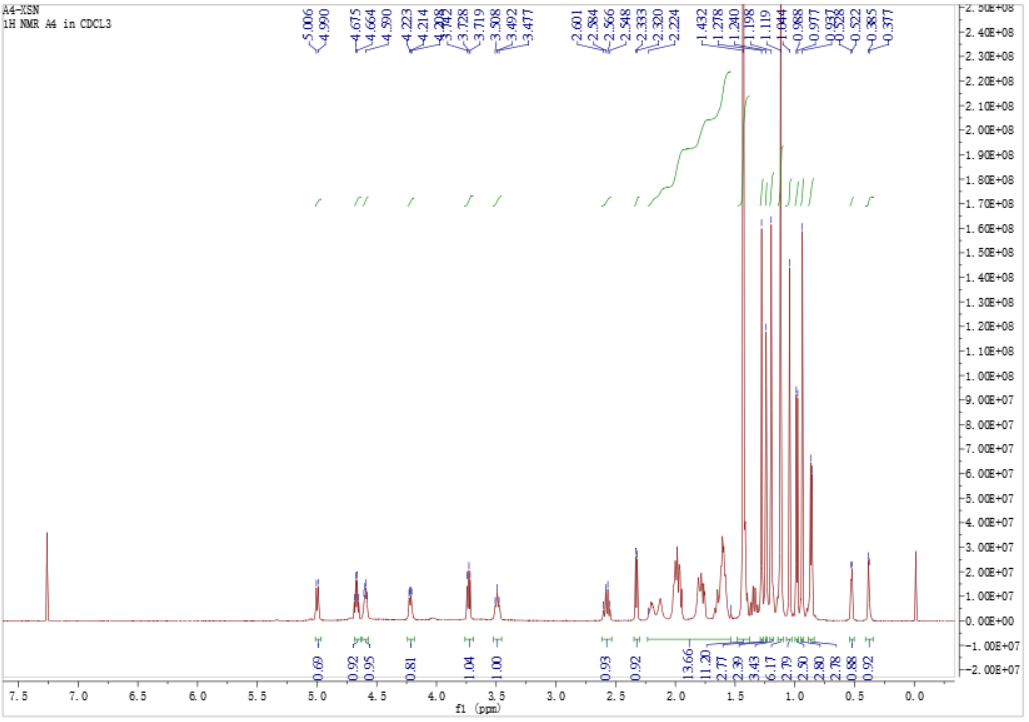


Figure.S10. ^1^H-NMR of compound A4


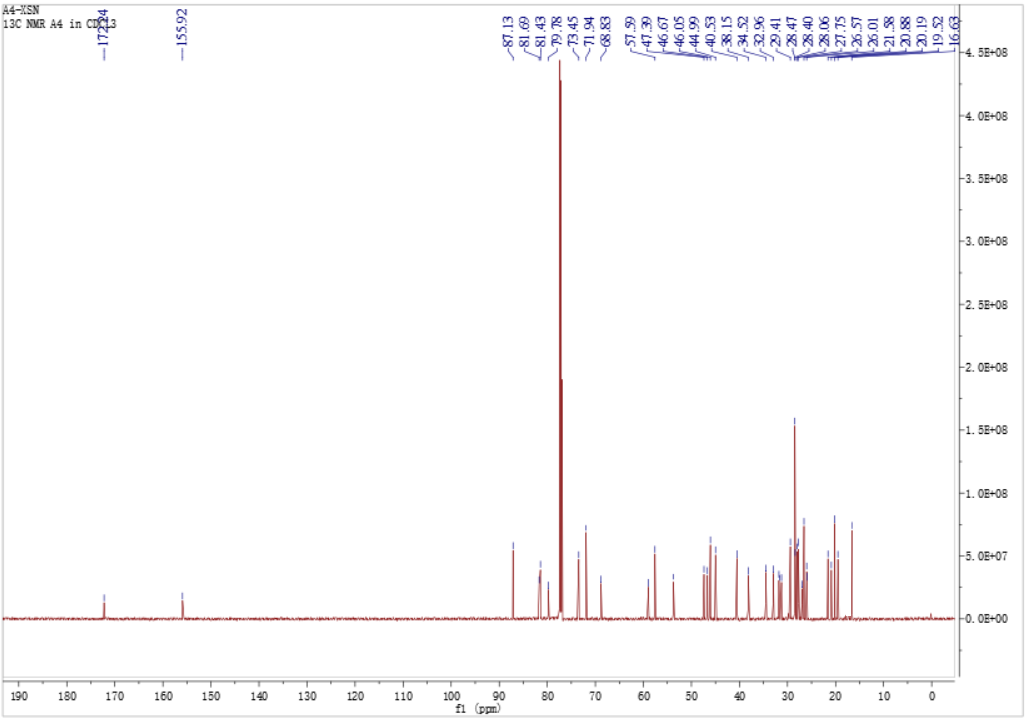


Figure.S11. ^13^C-NMR of compound A4


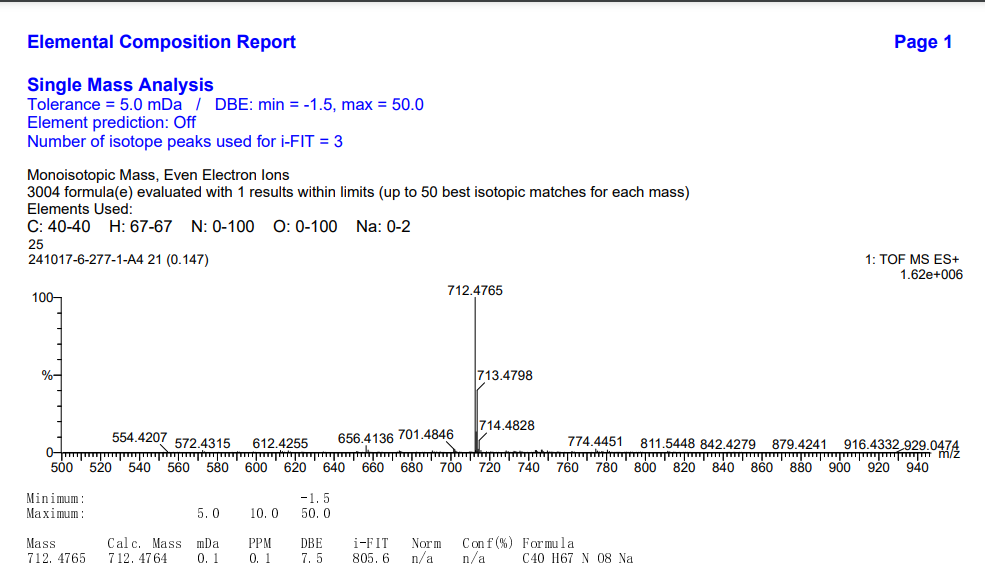


Figure.S12. HRMS of compound A4

### Compound A5

White powder, melting point: 230-232℃. ^1^H-NMR (CDCl_3_, 600 MHz, ppm): δ 0.39(d, *J* = 4.2 Hz, 1H, H-19b), 0.54(d, *J* = 4.2 Hz, 1H, H-19a), 0.95, 1.05, 1.11, 1.14, 1.22, 1.25, 1.29(s, 3H, -CH_3_×7), 1.44(s, 3H, -CH_3_), 2.34(d, *J* = 7.8 Hz, 1H), 2.60(q, *J* = 10.2 Hz, 1H), 3.53(t, *J_1_* = 3.6 Hz, *J_2_* = 10.2 Hz, 1H, H-6), 3.76(t, *J_1_* = 7.8 Hz, *J_2_* = 14.4 Hz, 1H, H-24), 4.30(t, *J_1_* = 6.6 Hz, *J_2_* = 13.8 Hz, 1H), 4.63(dd, *J_1_* = 4.8 Hz, *J_2_* = 10.8 Hz, 1H, H-3), 4.70(q, *J* = 7.8 Hz, 1H, H-16), 5.09(d, *J* = 7.2 Hz, 1H); ^13^C-NMR (CDCl_3_, 150 MHz, ppm): δ 173.17(-C=O), 87.19(C-20), 81.41(C-24), 81.50(C-3), 73.47(C-16), 71.96(C-25), 68.95(C-6), 57.60(C-17), 53.84(C-5), 49.68(-CH-NH_2_), 47.27(C-8), 46.61(C-14), 46.09(C-15), 45.10(C-13), 40.69(C-4), 38.16(C-7), 34.54(C-22), 33.54(C-1), 31.79 (C-12), 31.60(C-19), 29.84(C-2), 29.39(C-10), 28.29(C-28), 27.84(C-21), 26.90(C-11), 26.60(C-27), 26.00(C-26), 25.80(C-23), 21.61(C-9), 20.96(C-18), 20.19(C-30), 19.07(-CH-CH_3_), 16.54(C-29); ESI-HRMS (m/z): calcd for C_33_H_55_O_6_NNa^+^ [M+Na]^+^: 584.3927, found: 584.3920.


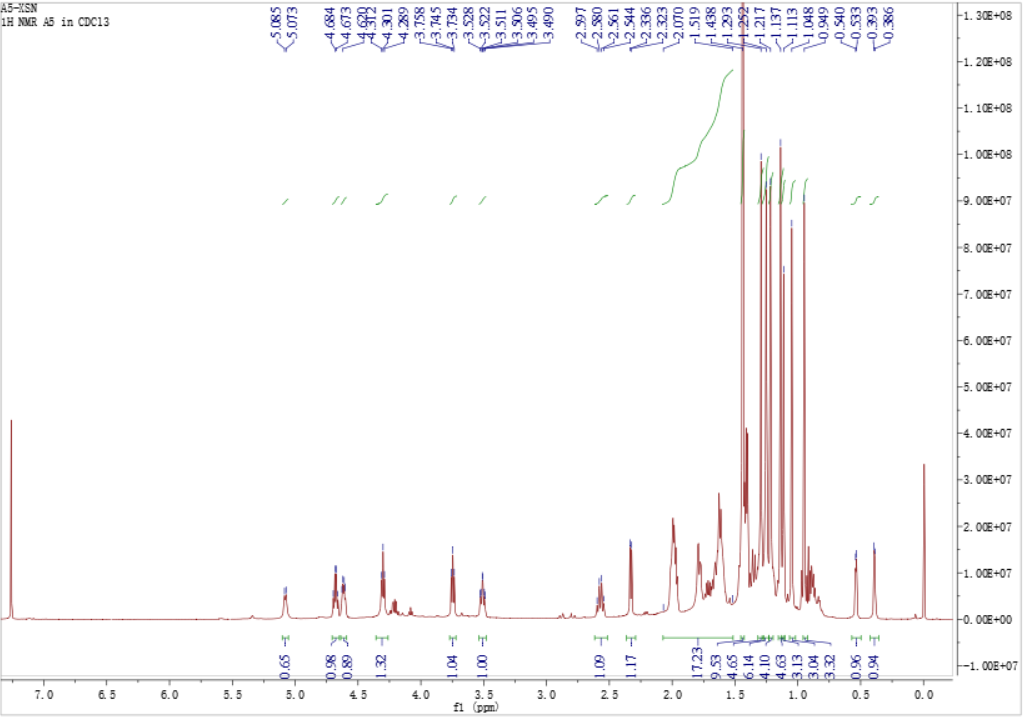


Figure.S13. ^1^H-NMR of compound A5


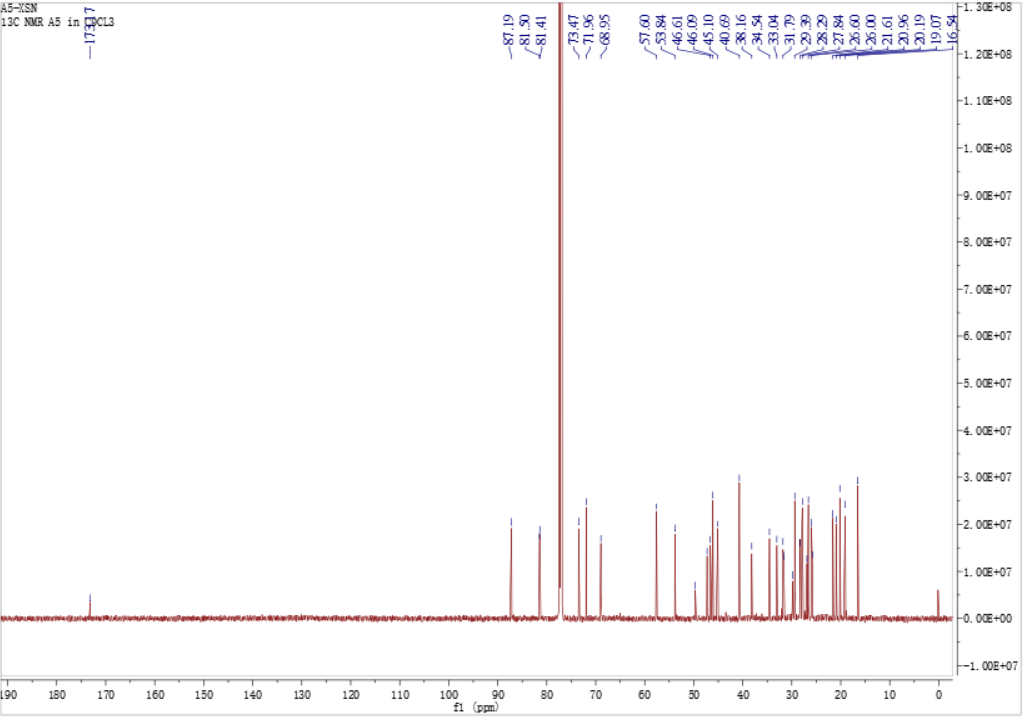


Figure.S14. ^13^C-NMR of compound A5


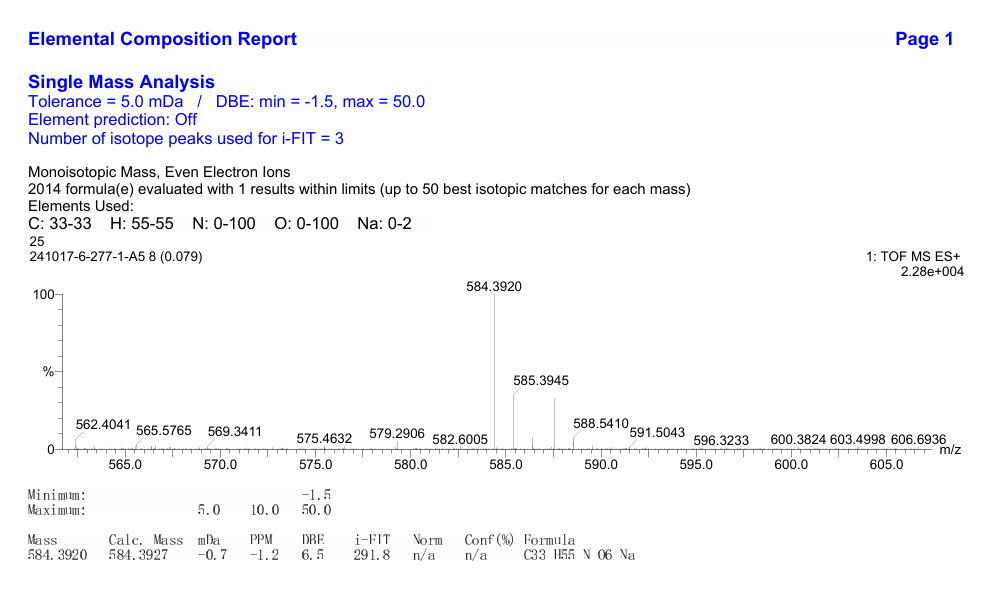


Figure.S15. HRMS of compound A5

### Compound A6

White powder, melting point: 240-242℃. ^1^H-NMR (CDCl_3_, 600 MHz, ppm): δ 0.37(d, *J* = 4.2 Hz, 1H, H-19b), 0.53(d, *J* = 3.0 Hz, 1H, H-19a), 0.95, 1.00, 1.06, 1.15, 1.23, 1.26, 1.30(s, 3H, -CH_3_×7), 2.34(d, *J* = 7.8 Hz, 1H), 2.60(q, *J* = 10.2 Hz, 1H), 3.00-3.16(m, 2H), 3.50(t, *J_1_* = 7.8 Hz, *J_2_* = 16.8 Hz, 1H, H-6), 3.77(t, *J_1_* = 7.2 Hz, *J_2_* = 14.4 Hz, 1H, H-24), 4.55-4.59(m, 2H), 4.70(q, *J* = 7.2 Hz, 1H, H-16), 4.90(d, *J* = 8.4 Hz, 1H), 7.17(d, *J* = 7.2 Hz, 2H, AR-H), 7.22-7.24(m, 1H, AR-H), 7.30(t, *J_1_* = 7.8 Hz, *J_2_* = 15.6 Hz, 2H, AR-H); ^13^C-NMR (CDCl_3_, 150 MHz, ppm): δ 171.95(-C=O), 136.21, 129.53, 128.67, 127.10(AR-C), 87.21(C-20), 81.51(C-24), 81.89(C-3), 73.48(C-16), 76.96(C-25), 68.94(C-6), 57.59(C-17), 54.76(-CH_2_-CH-NH_2_), 53.86(C-5), 47.17(C-8), 46.58(C-14), 46.09(C-15), 45.13(C-13), 40.55(C-4), 38.56(C_6_H_5_-CH_2_-CH-), 38.11(C-7), 34.55(C-22), 33.06(C-1), 31.77(C-12), 31.51(C-19), 29.85(C-2), 29.32(C-10), 27.92(C-28), 27.86(C-21), 26.77(C-11), 26.62(C-27), 25.98(C-26), 25.75(C-23), 21.61(C-9), 20.96(C-18), 20.18(C-30), 16.57(C-29); ESI-HRMS (m/z): calcd for C_39_H_60_O_6_NNa^+^ [M+Na]^+^: 638.4421, found: 638.4414.


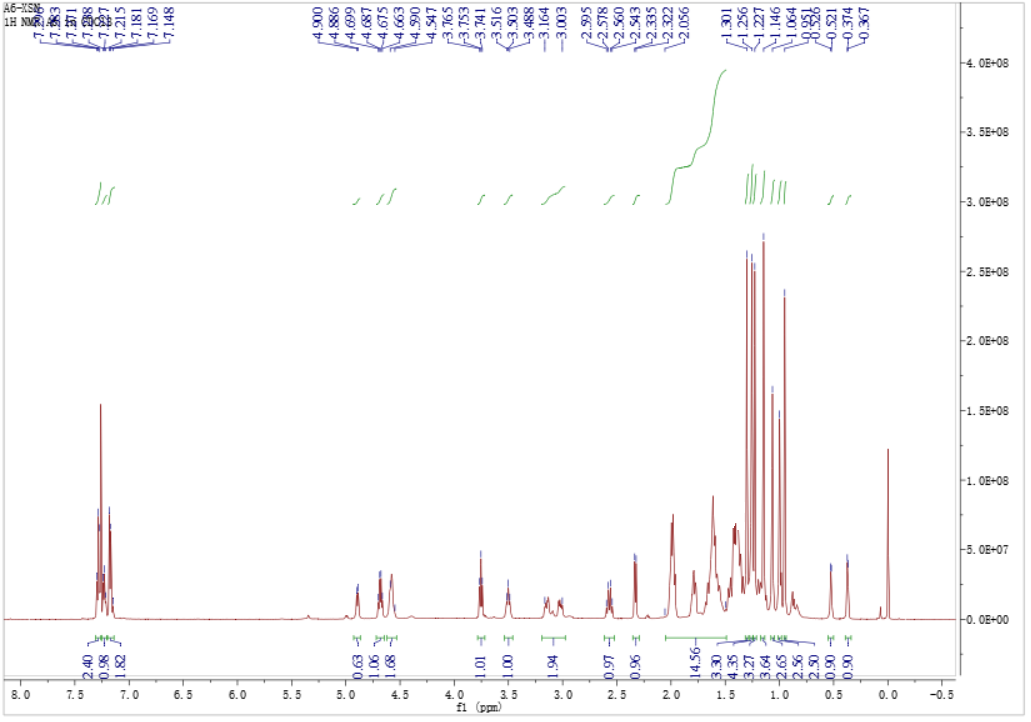


Figure.S16. ^1^H-NMR of compound A6


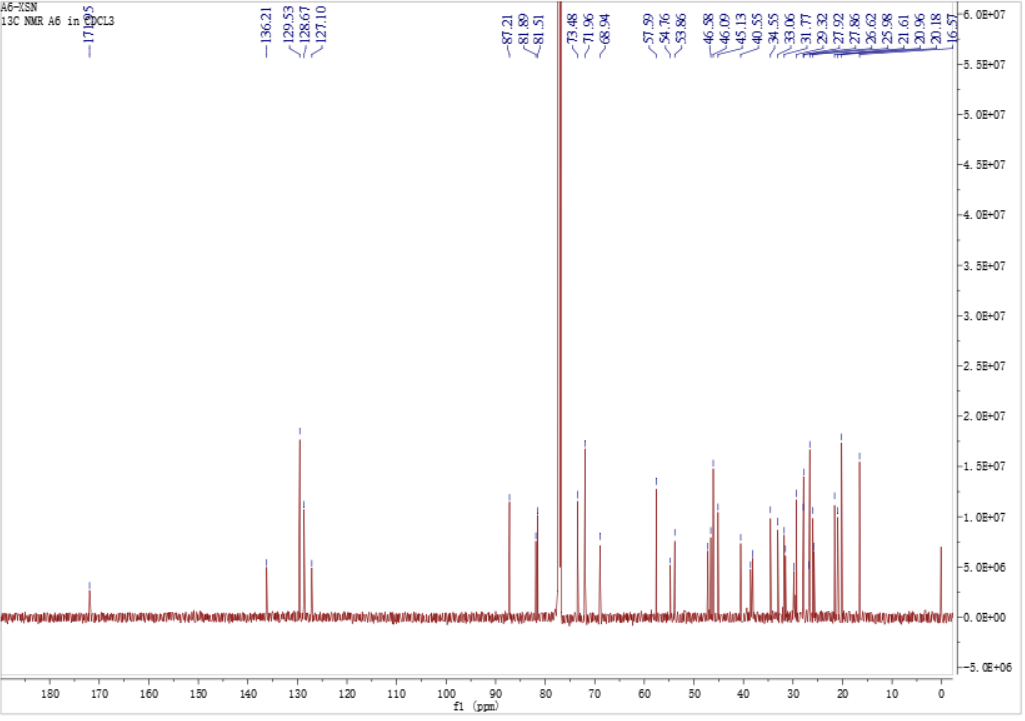


Figure.S17. ^13^C-NMR of compound A6


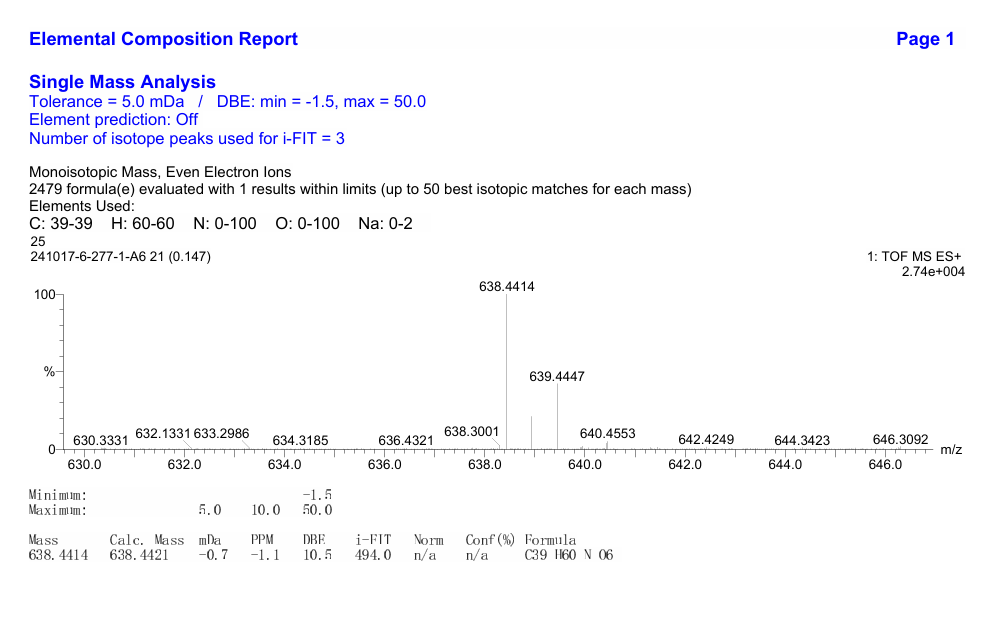


Figure.S18. HRMS of compound A6

### Compound A7

White powder, melting point: 226-228℃. ^1^H-NMR (CDCl_3_, 600 MHz, ppm): δ 0.38(d, *J* = 4.2 Hz, 1H, H-19b), 0.53(d, *J* = 4.2 Hz, 1H, H-19a), 0.94, 1.03, 1.11, 1.13, 1.21, 1.25, 1.29(s, 3H, -CH_3_×7), 2.33(d, *J* = 7.8 Hz, 1H), 2.60(q, *J* = 10.8 Hz, 1H), 3.51(t, *J_1_* = 8.4 Hz, *J_2_* = 16.8 Hz, 1H, H-6), 3.75(t, *J_1_* = 7.8 Hz, *J_2_* = 14.4 Hz, 1H, H-24), 3.87-3.94(m, 2H, -NH-CH_2_-CO-), 4.61-4.69(m, 2H, H-16 and H-3), 5.02(s, 1H); ^13^C-NMR (CDCl_3_, 150 MHz, ppm): δ 170.25(-C=O), 87.15(C-20), 81.46(C-24), 81.67(C-3), 73.45(C-16), 71.94(C-25), 68.83(C-6), 57.59(C-17), 53.74(C-5), 47.33(C-8), 46.63(C-14), 46.06(C-15), 45.02(C-13), 42.76(NH_2_-CH_2_-CO), 40.61(C-4), 38.13(C-7), 34.53(C-22), 32.99(C-1), 31.79(C-12), 31.67(C-19), 29.83(C-2), 29.38(C-10), 28.33(C-28), 27.78(C-21), 26.87(C-11), 26.59(C-27), 26.01(C-26), 25.89(C-23), 21.59(C-9), 20.90(C-18), 20.18(C-30), 16.46(C-29); ESI-HRMS (m/z): calcd for C_32_H_53_O_6_NNa^+^ [M+Na]^+^: 570.3771, found: 570.3768.


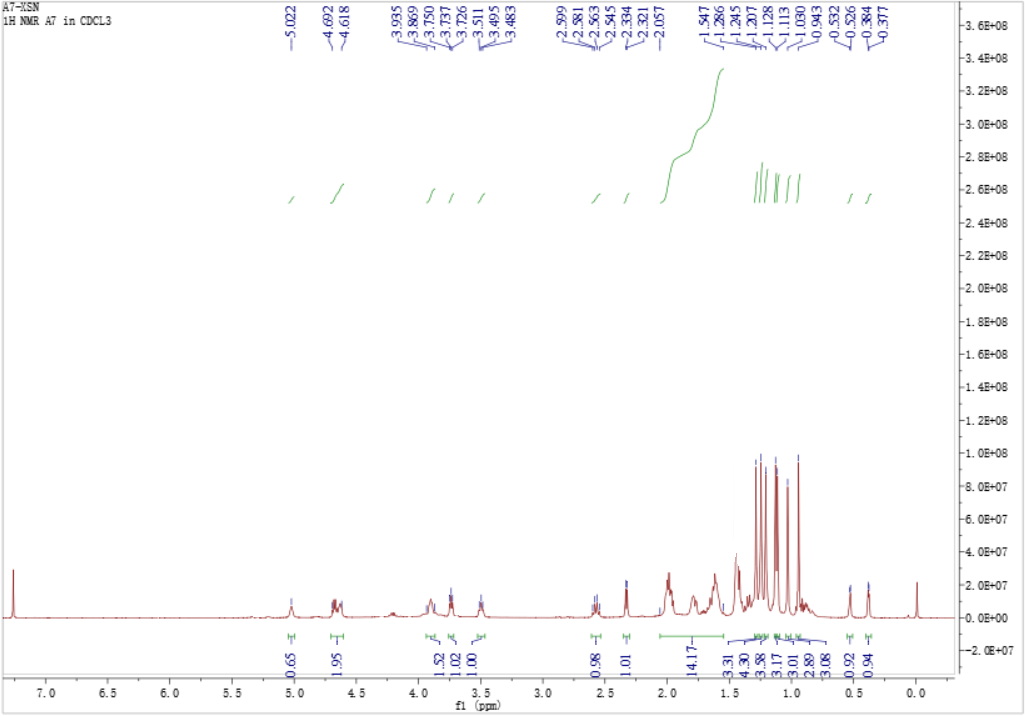


Figure.S19. ^1^H-NMR of compound A7


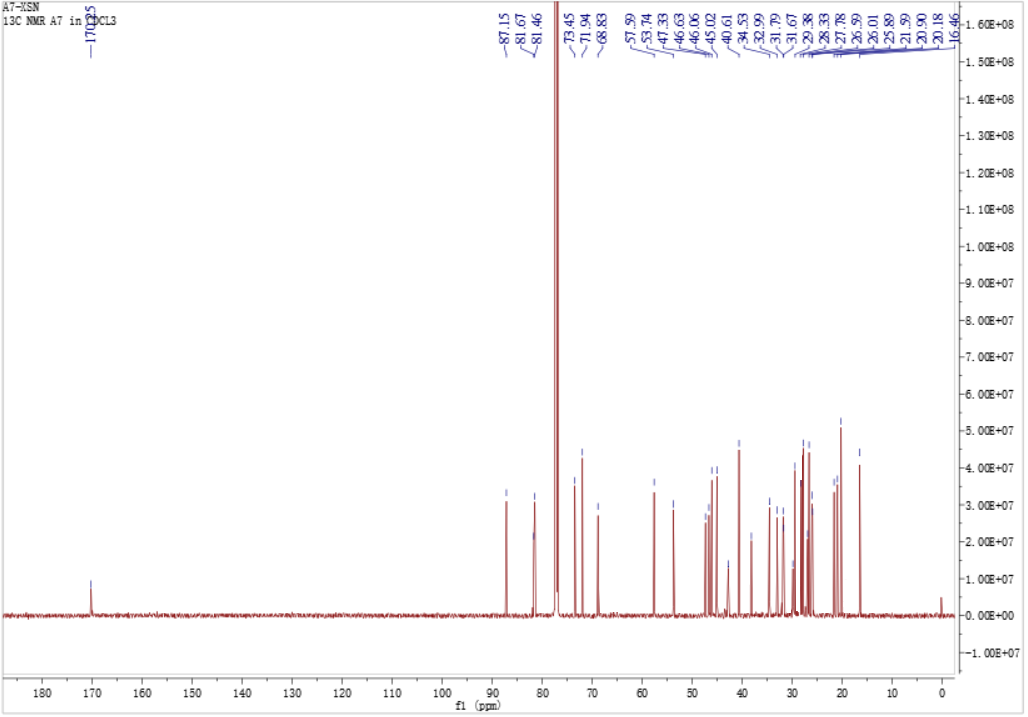


Figure.S20. ^13^C-NMR of compound A7


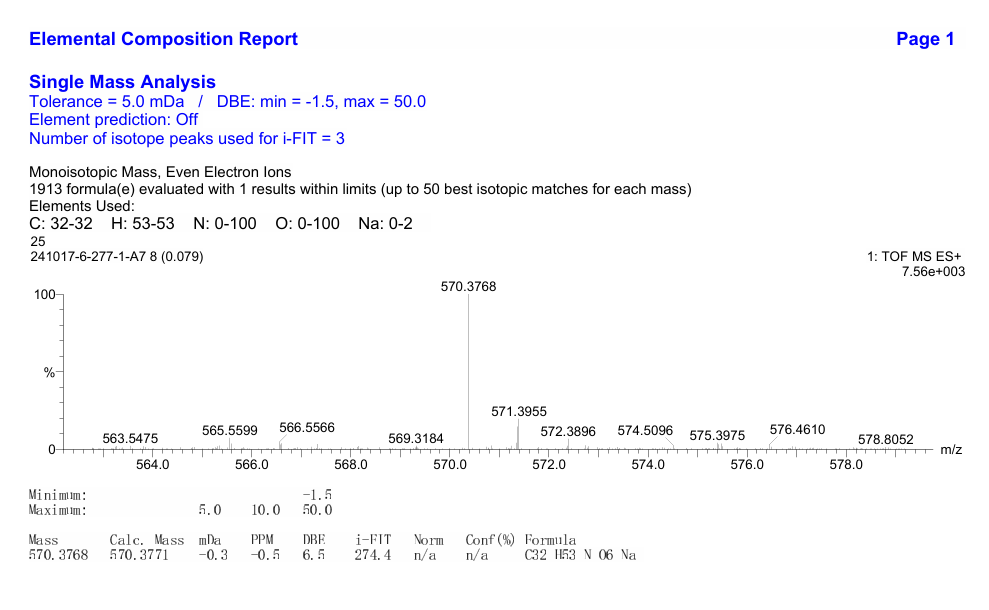


Figure.S21. HRMS of compound A7

### Compound A8

White powder, melting point: 234-236℃. ^1^H-NMR (CDCl_3_, 600 MHz, ppm): δ 0.39(d, *J* = 4.2 Hz, 1H, H-19b), 0.54(d, *J* = 4.2 Hz, 1H, H-19a), 0.86-0.88(m, 3H, CH_3_-CH-CH_3_), 0.99(d, *J* = 6.6 Hz, 3H, CH_3_-CH-CH_3_), 0.95, 1.05, 1.12, 1.23, 1.21, 1.25, 1.29(s, 3H, -CH_3_×7), 2.34(d, *J* = 7.8 Hz, 1H), 2.60(q, *J* = 10.2 Hz, 1H), 3.52(t, *J_1_* = 3.0 Hz, *J_2_* = 9.6 Hz, 1H, H-6), 3.74(t, *J_1_* = 7.8 Hz, *J_2_* = 14.4 Hz, 1H, H-24), 4.20-4.24(m, 1H), 4.30(t, *J_1_* = 7.2 Hz, *J_2_* = 13.8 Hz, 1H), 4.61(dd, *J_1_* = 4.8 Hz, *J_2_* = 10.8 Hz, 1H, H-3), 4.70(q, *J* = 7.8 Hz, 1H, H-16), 5.00(d, *J* = 9.0 Hz, 1H); ^13^C-NMR (CDCl_3_, 150 MHz, ppm): δ 172.23(-C=O), 87.15(C-20), 81.46(C-24), 81.66(C-3), 73.45(C-16), 71.94(C-25), 68.87(C-6), 58.99(NH_2_-CH-CO-), 57.59(C-17), 53.80(C-5), 47.34(C-8), 46.65(C-14), 46.06(C-15), 45.03(C-13), 40.53(C-4), 38.16(C-7), 34.53(C-22), 32.99(C-1), 31.79(C-12), 31.65(C-19), 31.25(C-2), 29.40(C-10), 28.39(C-28), 28.01(-CH(CH_3_)_2_), 27.78(C-21), 26.98(C-11), 26.58(C-27), 26.01(C-26), 25.89(C-23), 21.59(C-9), 20.91(C-18), 20.18(C-30), 19.31(-CH(CH_3_)_2_), 16.64(C-29); ESI-HRMS (m/z): calcd for C_35_H_60_O_6_N [M]^+^: 590.4421, found: 590.4420.


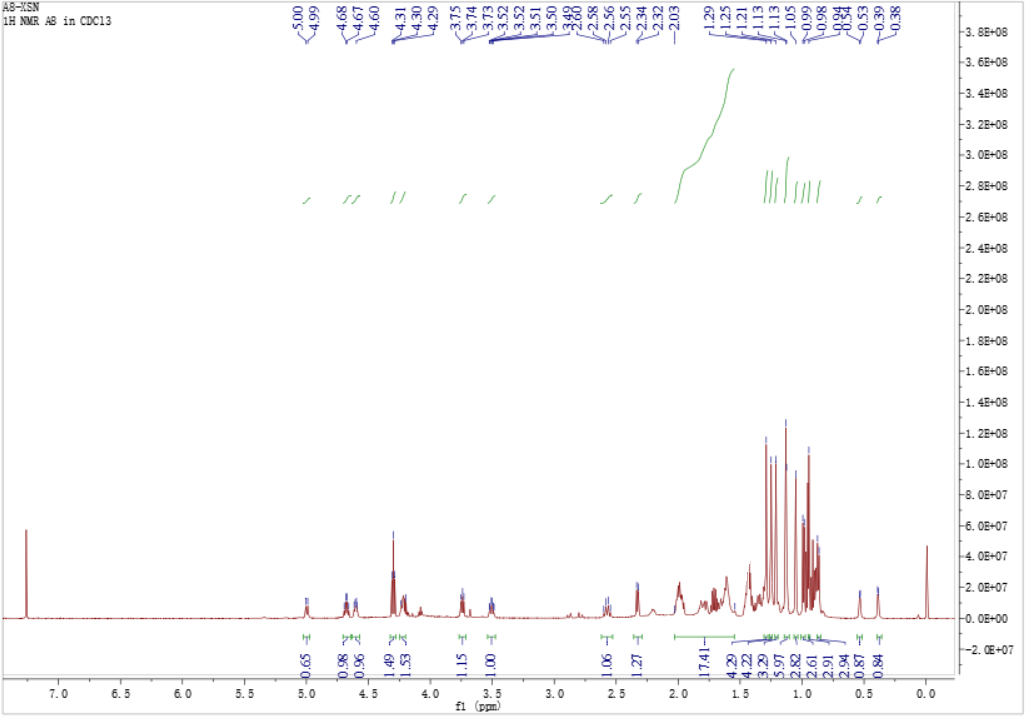


Figure.S22. ^1^H-NMR of compound A8


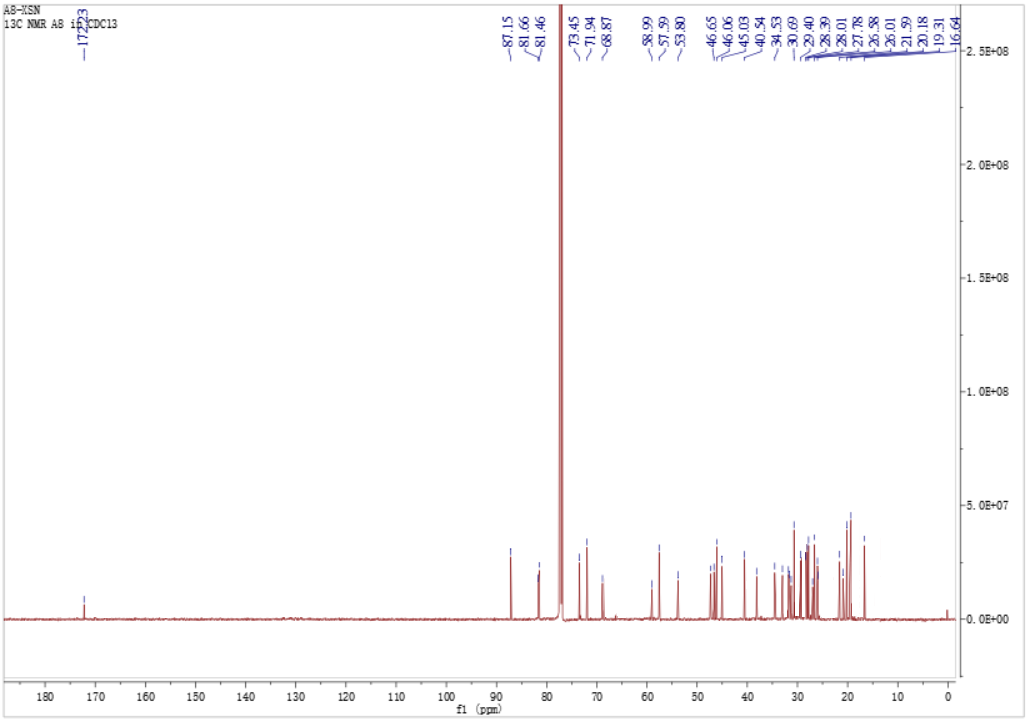


Figure.S23. ^13^C-NMR of compound A8


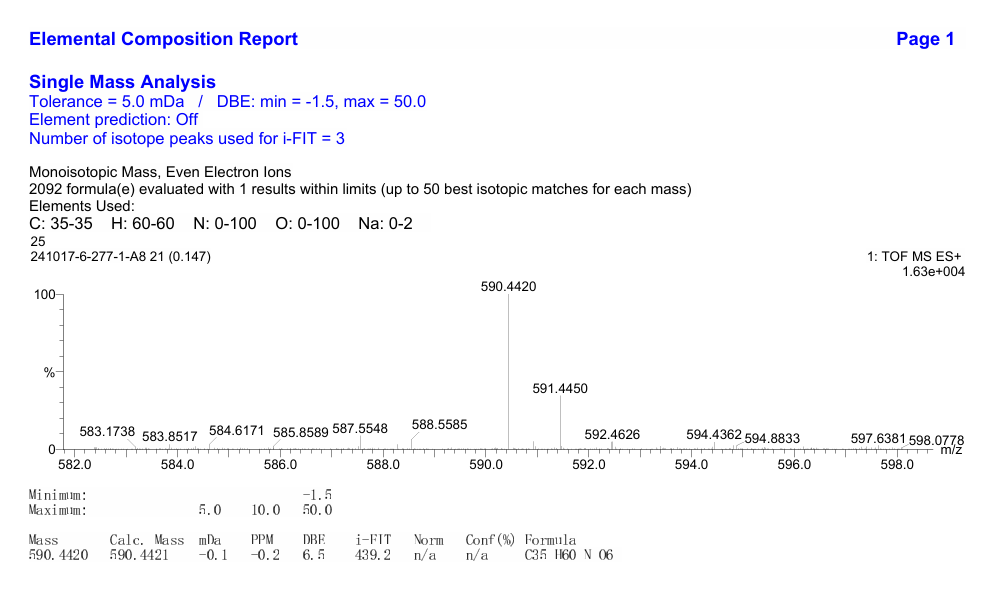


Figure.S24. HRMS of compound A8

### Compound B1

White powder, melting point: 229-231℃. ^1^H-NMR (CDCl_3_, 600 MHz, ppm): δ 0.43(d, *J* = 4.2 Hz, 1H, H-19b), 0.59(d, *J* = 4.2 Hz, 1H, H-19a), 0.98, 1.16, 1.21, 1.24, 1.25, 1.28, 1.31(s, 3H, -CH_3_×7), 2.34(d, *J* = 7.8 Hz, 1H), 2.58(q, *J* = 10.2 Hz, 1H), 3.57(td, *J_1_* = 2.4 Hz, *J_2_* = 9.6 Hz, 1H, H-6), 3.75(t, *J_1_* = 7.2 Hz, *J_2_* = 14.4 Hz, 1H, H-24), 4.70(q, *J* = 7.2 Hz, 1H, H-16), 4.82(dd, *J_1_* = 4.8 Hz, *J_2_* = 10.8 Hz, 1H, H-3), 7.45(t, *J_1_* = 7.2 Hz, *J_2_* = 14.4 Hz, 2H, AR-H), 7.55(t, *J_1_* = 7.2 Hz, *J_2_* = 14.4 Hz, 1H, AR-H), 8.06(d, *J* = 7.8 Hz, 2H, AR-H); ^13^C-NMR (CDCl_3_, 150 MHz, ppm): δ 166.39(-C=O), 132.92, 131.00, 129.70, 128.49(AR-C), 87.24(C-20), 80.97(C-24), 81.55(C-3), 73.51(C-16), 71.98(C-25), 69.09(C-6), 57.61(C-17), 54.04(C-5), 47.25(C-8), 46.60(C-14), 46.15(C-15), 45.18(C-13), 40.94(C-4), 38.14(C-7), 34.56(C-22), 33.11(C-1), 31.91(C-12), 31.61(C-19), 29.85(C-2), 29.51(C-10), 28.45(C-28), 27.89(C-21), 27.00(C-11), 26.62(C-27), 26.03(C-26), 25.72(C-23), 21.66(C-9), 21.02(C-18), 20.22(C-30), 16.81(C-29); ESI-HRMS (m/z): calcd for C_37_H_54_O_6_Na^+^ [M+Na]^+^: 617.3818, found: 617.3822.


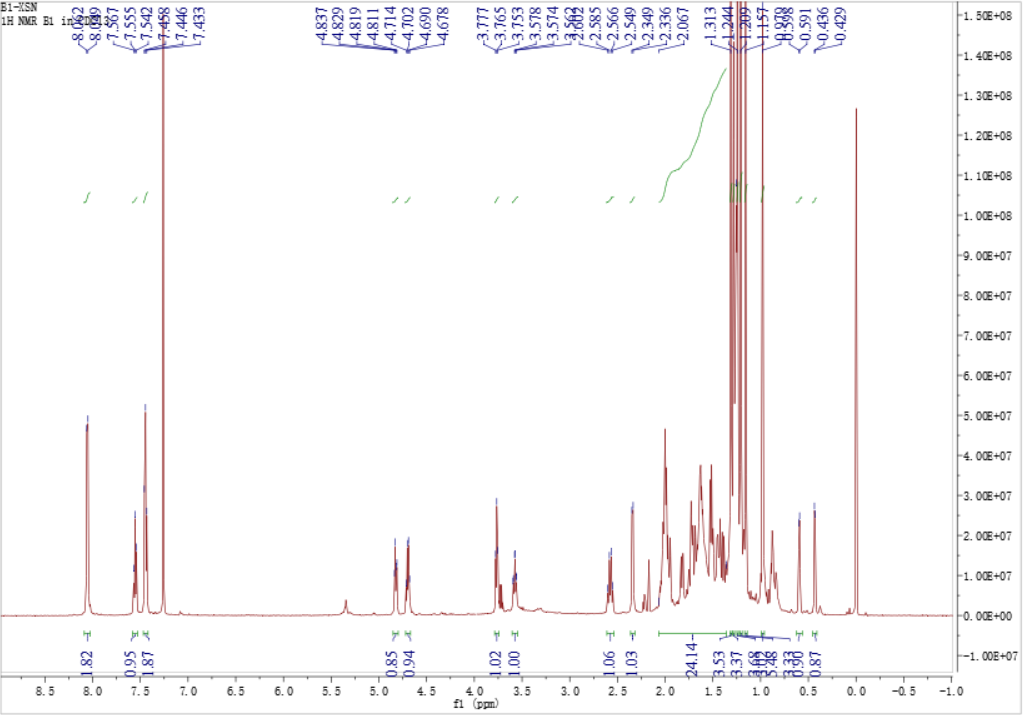


Figure.S25. ^1^H-NMR of compound B1


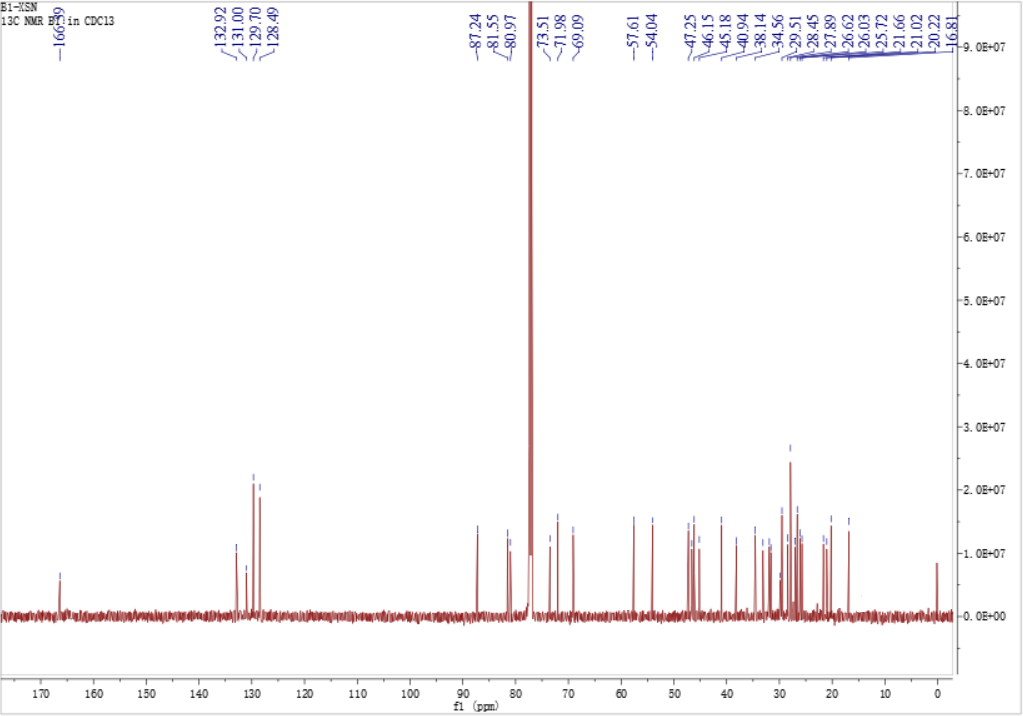


Figure.S26. ^13^C-NMR of compound B1


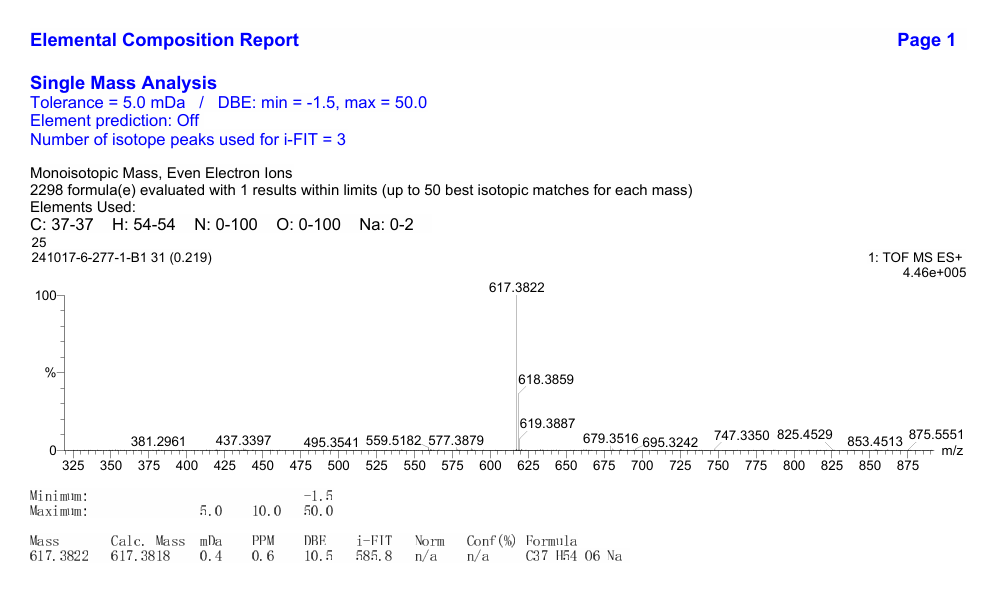


Figure.S27. HRMS of compound B1

### Compound B2

White powder, melting point: 228-230℃. ^1^H-NMR (CDCl_3_, 600 MHz, ppm): δ 0.43(d, *J* = 4.2 Hz, 1H, H-19b), 0.59(d, *J* = 3.6 Hz, 1H, H-19a), 0.97, 1.15, 1.23, 1.27, 1.30(s, 3H, -CH_3_×5), 1.19(s, 3H, -CH_3_×2), 2.36(d, *J* = 7.8 Hz, 1H), 2.59(q, *J* = 10.8 Hz, 1H), 3.57(td, *J_1_* = 3.0 Hz, *J_2_* = 9.6 Hz, 1H, H-6), 3.76(t, *J_1_* = 7.2 Hz, *J_2_* = 13.8 Hz, 1H, H-24), 4.72(q, *J* = 7.2 Hz, 1H, H-16), 4.81(dd, *J_1_* = 3.6 Hz, *J_2_* = 10.2 Hz, 1H, H-3), 7.11(t, *J_1_* = 9.0 Hz, *J_2_* = 16.8 Hz, 2H, AR-H), 8.05(t, *J_1_* = 7.8 Hz, *J_2_* = 13.2 Hz, 2H, AR-H); ^13^C-NMR (CDCl_3_, 150 MHz, ppm): δ 165.44(-C=O), 132.21, 132.15, 127.22, 127.20, 115.68, 115.53(AR-C), 87.20(C-20), 81.20(C-24), 81.50(C-3), 73.51(C-16), 72.01(C-25), 69.02(C-6), 57.60(C-17), 53.94(C-5), 47.33(C-8), 46.58(C-14), 46.13(C-15), 45.11(C-13), 40.92(C-4), 38.18(C-7), 34.56(C-22), 33.05(C-1), 31.89(C-12), 31.69(C-19), 30.50(C-2), 29.52(C-10), 28.45(C-28), 27.81(C-21), 27.00(C-11), 26.60(C-27), 26.03(C-26), 25.83(C-23), 21.64(C-9), 21.00(C-18), 20.21(C-30), 16.80(C-29); ESI-HRMS (m/z): calcd for C_37_H_53_O_6_FNa^+^ [M+Na]^+^: 635.3724, found: 635.3728.


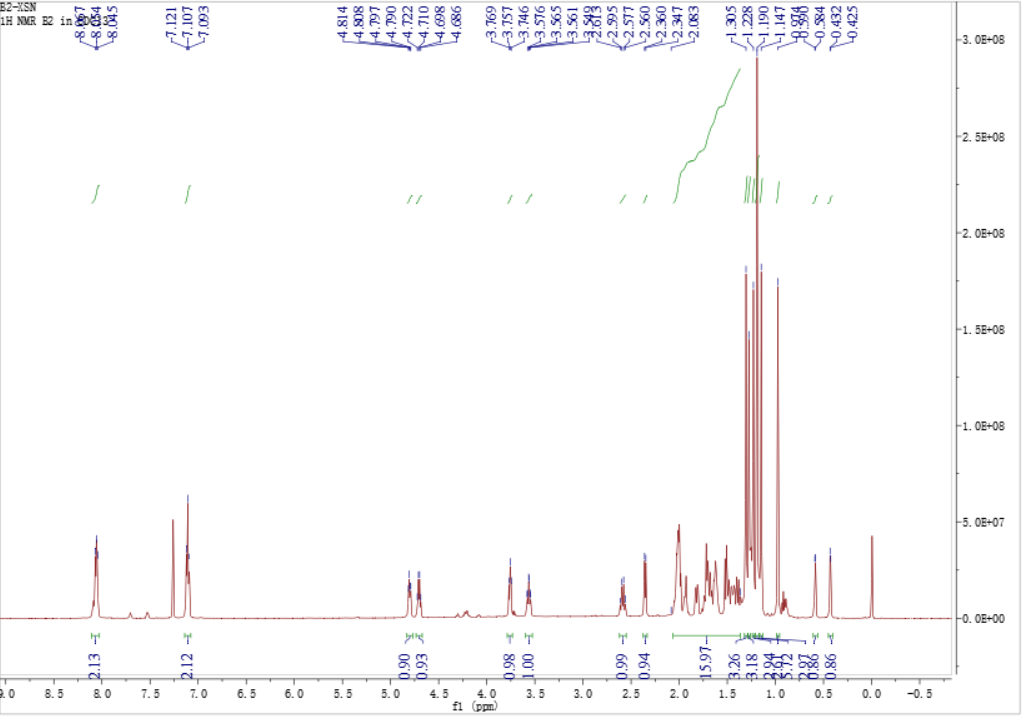


Figure.S28. ^1^H-NMR of compound B2


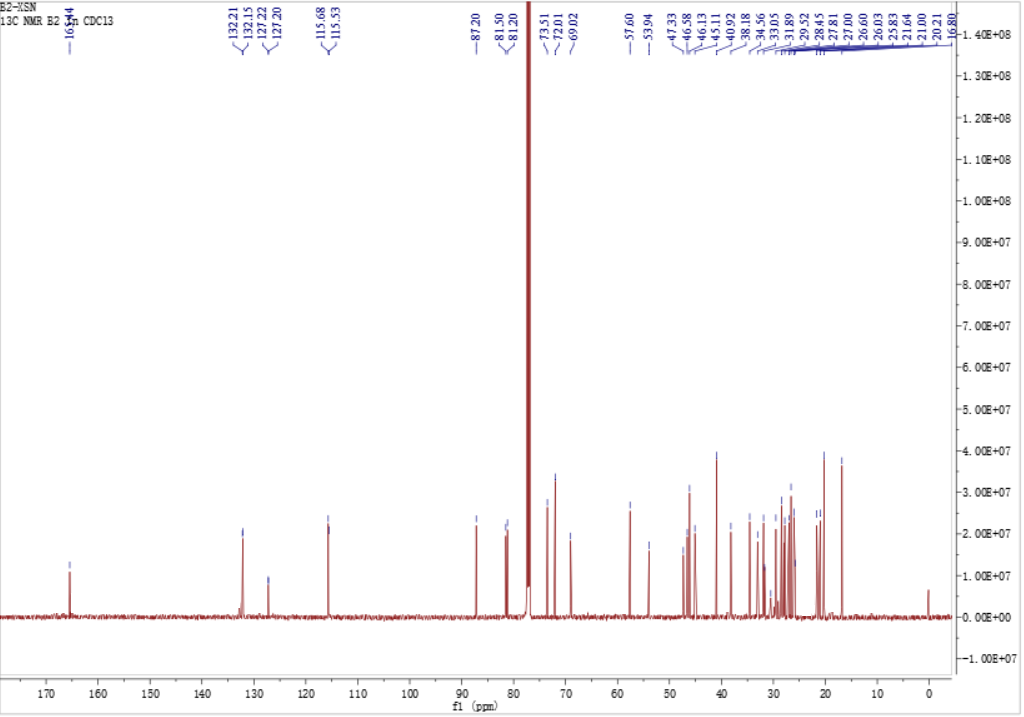


Figure.S29. ^13^C-NMR of compound B2


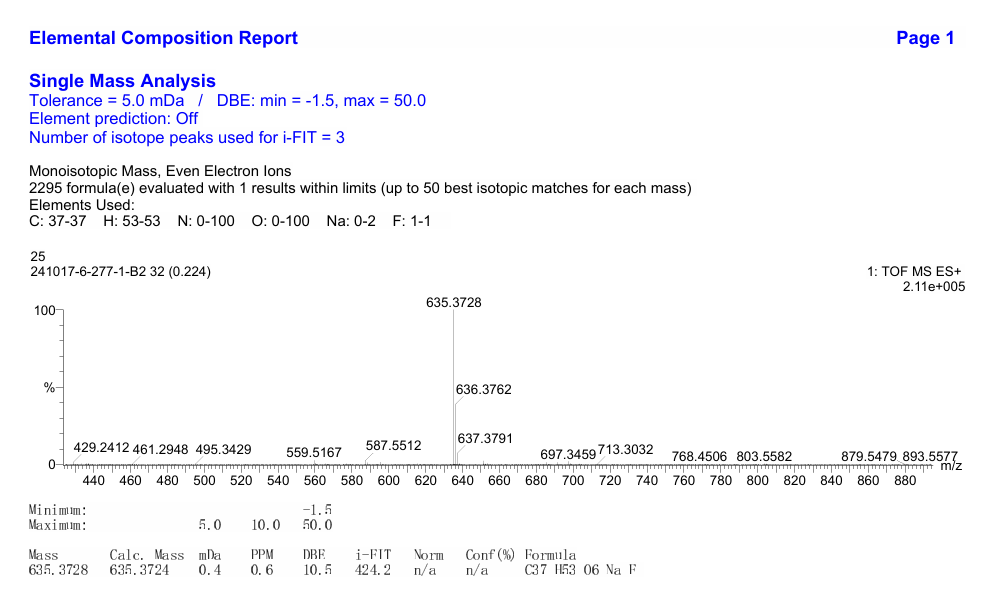


Figure.S30. HRMS of compound B2

### Compound B3

White powder, melting point: 228-230℃. ^1^H-NMR (CDCl_3_, 600 MHz, ppm): δ 0.43(d, *J* = 4.8 Hz, 1H, H-19b), 0.59(d, *J* = 4.2 Hz, 1H, H-19a), 0.97, 1.15, 1.23, 1.27, 1.31(s, 3H, -CH_3_×5), 1.18(s, 3H, -CH_3_×2), 2.36(d, *J* = 8.4 Hz, 1H), 2.61(q, *J* = 10.2 Hz, 1H), 3.57(td, *J_1_* = 3.0 Hz, *J_2_* = 9.6 Hz, 1H, H-6), 3.76(t, *J_1_* = 7.8 Hz, *J_2_* = 14.4 Hz, 1H, H-24), 4.72(q, *J* = 7.8 Hz, 1H, H-16), 4.81(dd, *J_1_* = 4.8 Hz, *J_2_* = 10.8 Hz, 1H, H-3), 7.41(t, *J_1_* = 7.8 Hz, *J_2_* = 15.6 Hz, 2H, AR-H), 7.97-8.01(m, 2H, AR-H); ^13^C-NMR (CDCl_3_, 150 MHz, ppm): δ 165.55(-C=O), 139.55, 131.67, 131.07, 129.42, 128.88, 128.83(AR-C), 87.21(C-20), 81.36(C-24), 81.48(C-3), 73.55(C-16), 72.06(C-25), 69.02(C-6), 57.59(C-17), 53.89(C-5), 47.34(C-8), 46.50(C-14), 46.13(C-15), 45.10(C-13), 40.92(C-4), 38.17(C-7), 34.56(C-22), 33.03(C-1), 31.88(C-12), 31.68(C-19), 29.51(C-2), 28.45(C-10), 28.03(C-28), 27.77(C-21), 26.98(C-11), 26.60(C-27), 26.03(C-26), 25.87(C-23), 21.63(C-9), 20.99(C-18), 20.21(C-30), 16.80(C-29); ESI-HRMS (m/z): calcd for C_37_H_53_O_6_ClNa^+^ [M+Na]^+^: 651.3428, found: 651.3431.


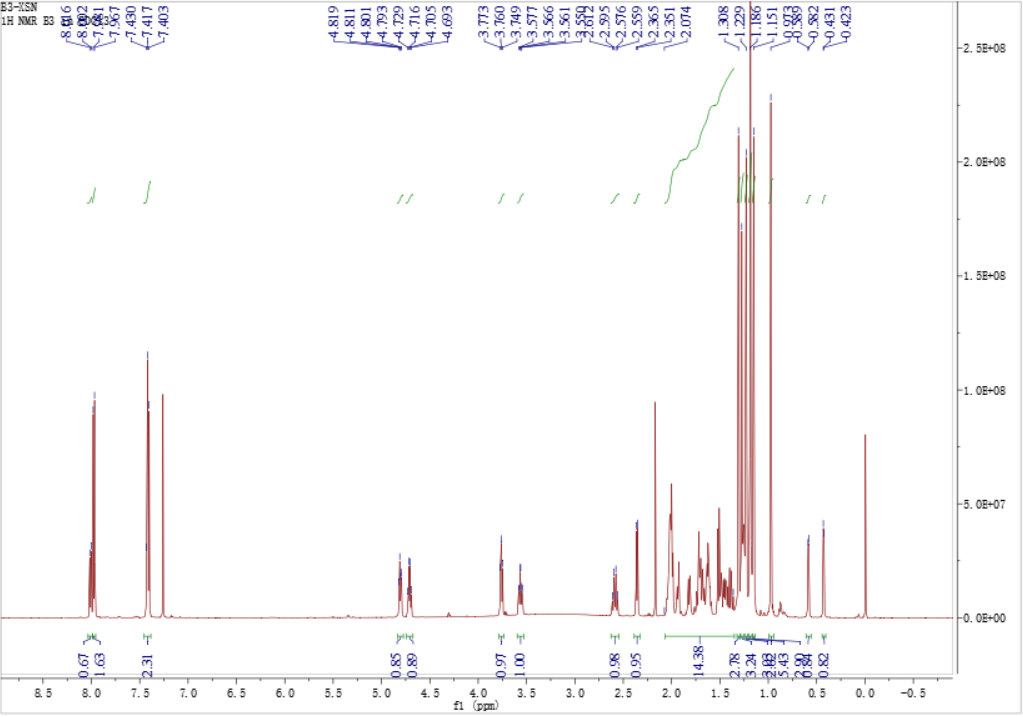


Figure.S31. ^1^H-NMR of compound B3


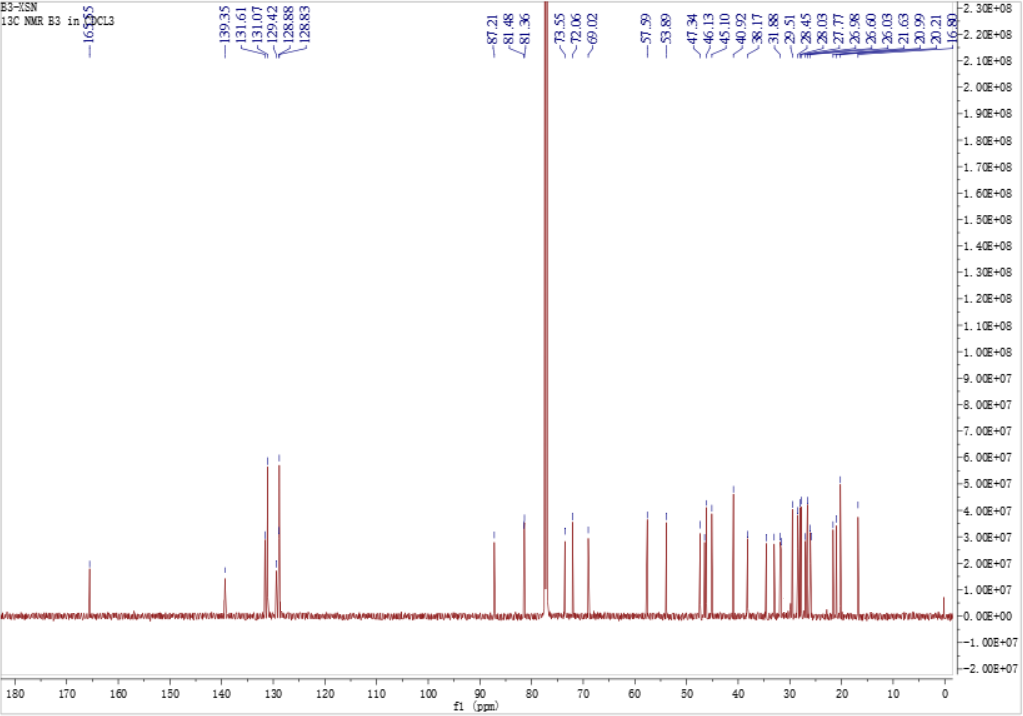


Figure.S32. ^13^C-NMR of compound B3


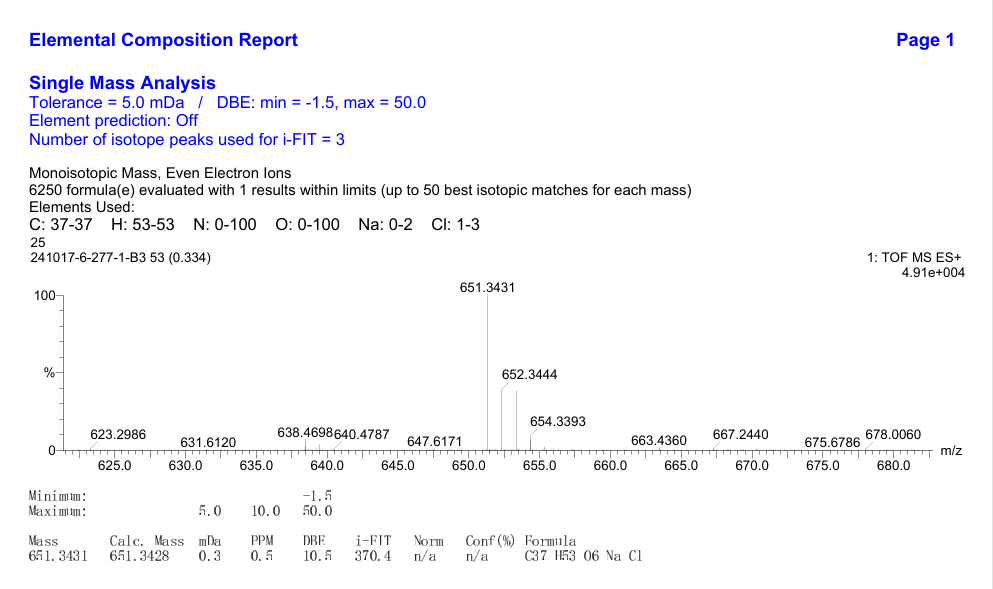


Figure.S33. HRMS of compound B3

### Compound B4

White powder, melting point: 234-236℃. ^1^H-NMR (CDCl_3_, 600 MHz, ppm): δ 0.43(d, *J* = 4.2 Hz, 1H, H-19b), 0.59(d, *J* = 4.2 Hz, 1H, H-19a), 0.97, 1.14, 1.22, 1.27, 1.30(s, 3H, -CH_3_×5), 1.18(s, 3H, -CH_3_×2), 2.36(d, *J* = 7.8 Hz, 1H), 2.61(q, *J* = 10.2 Hz, 1H), 3.57(td, *J_1_* = 3.6 Hz, *J_2_* = 10.2 Hz, 1H, H-6), 3.75(t, *J_1_* = 7.8 Hz, *J_2_* = 14.4 Hz, 1H, H-24), 4.72(q, *J* = 7.8 Hz, 1H, H-16), 4.81(dd, *J_1_* = 4.2 Hz, *J_2_* = 10.8 Hz, 1H, H-3), 7.58(d, *J* = 8.4 Hz, 2H, AR-H), 7.90(d, *J* = 8.4 Hz, 2H, AR-H); ^13^C-NMR (CDCl_3_, 150 MHz, ppm): δ 165.67(-C=O), 131.83, 131.22, 129.88, 128.00(AR-C), 87.20(C-20), 81.40(C-24), 81.49(C-3), 73.53(C-16), 72.04(C-25), 69.00(C-6), 57.59(C-17), 53.88(C-5), 47.35(C-8), 46.54(C-14), 46.12(C-15), 45.09(C-13), 40.91(C-4), 38.18(C-7), 34.56(C-22), 33.03(C-1), 31.88(C-12), 31.70(C-19), 29.51(C-2), 28.45(C-10), 28.02(C-28), 27.78(C-21), 26.97(C-11), 26.60(C-27), 26.03(C-26), 25.87(C-23), 21.63(C-9), 20.99(C-18), 20.21(C-30), 16.80(C-29); ESI-HRMS (m/z): calcd for C_37_H_53_O_6_BrNa^+^ [M+Na]^+^: 695.2923, found: 695.2927.


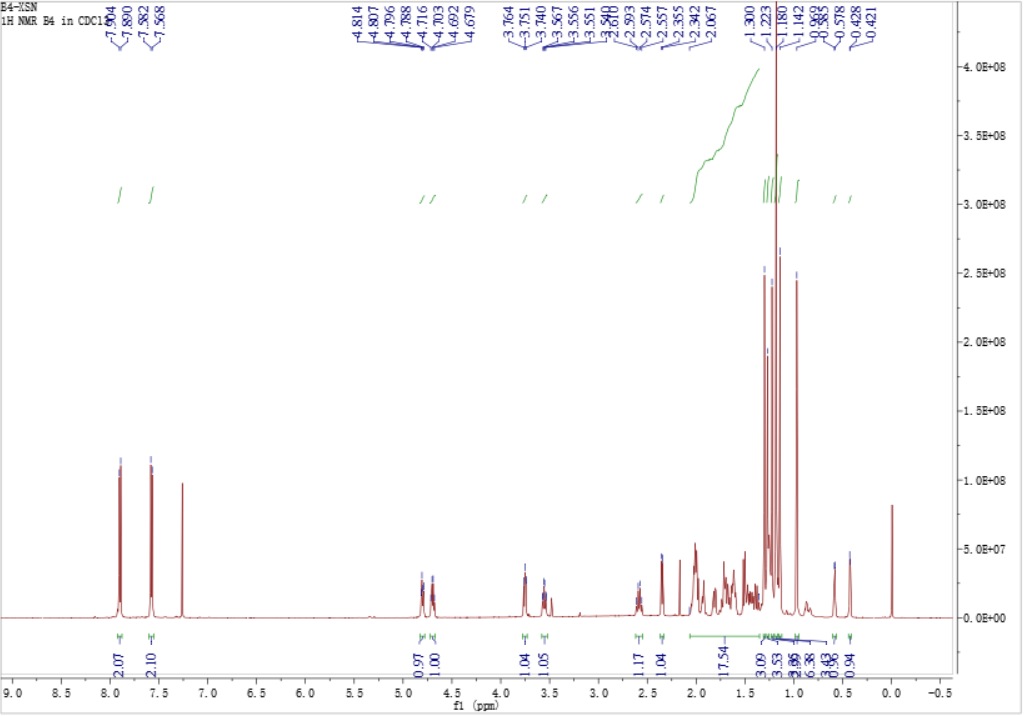


Figure.S34. ^1^H-NMR of compound B4


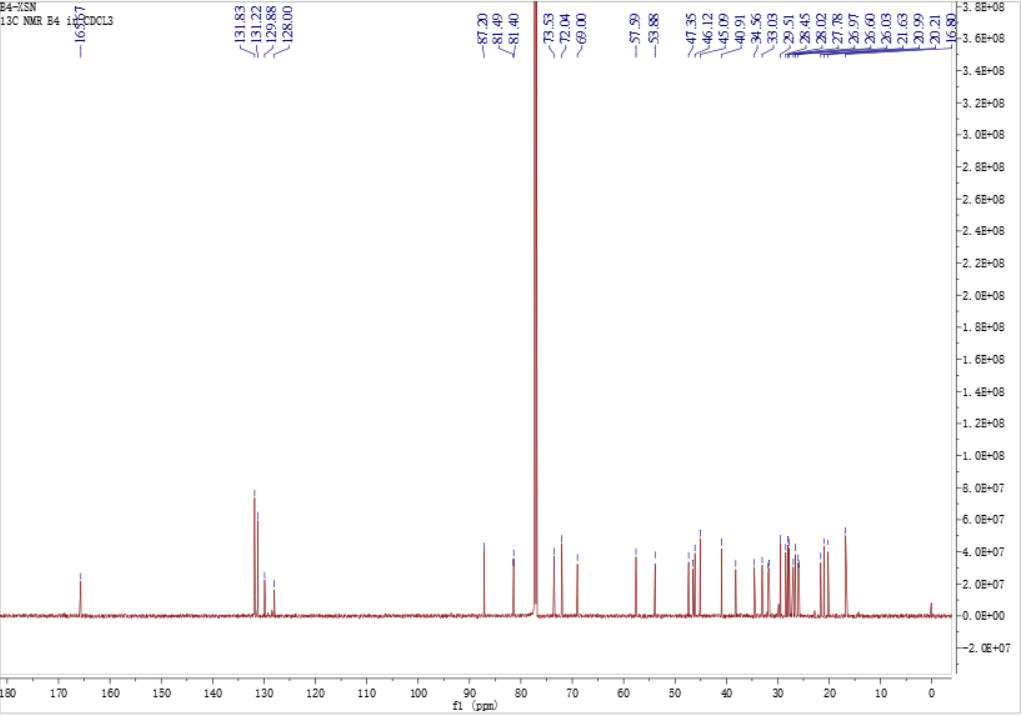


Figure.S35. ^13^C-NMR of compound B4


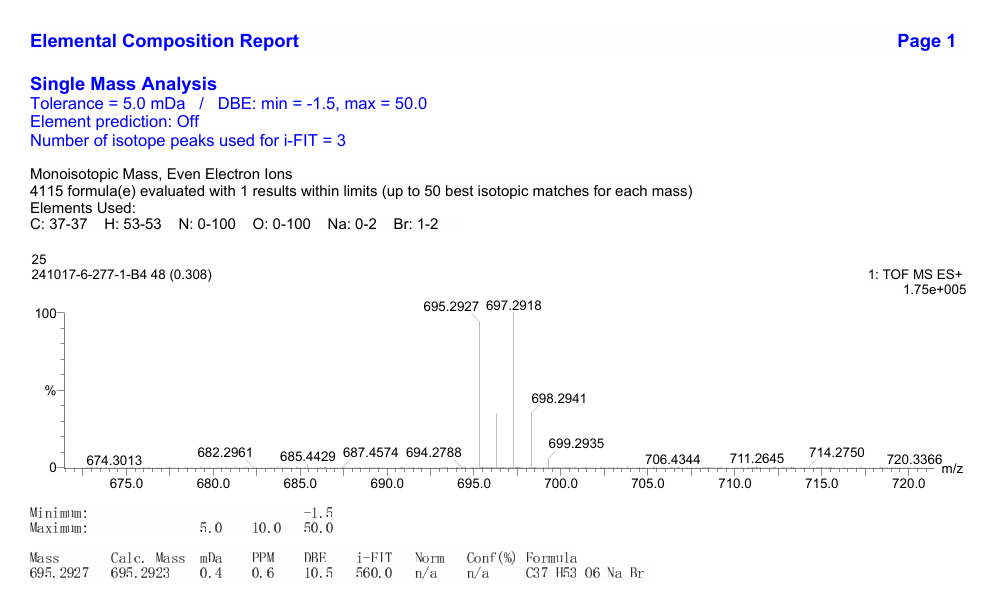


Figure.S36. HRMS of compound B4

### Compound C1

White powder, melting point: 240-242℃. ^1^H-NMR (CDCl_3_, 600 MHz, ppm): δ 0.44(d, *J* = 3.6 Hz, 1H, H-19b), 0.60(d, *J* = 3.6 Hz, 1H, H-19a), 0.98, 1.51, 1.23, 1.28, 1.31(s, 3H, -CH_3_×5), 1.21(s, 3H, -CH_3_×2), 2.35(d, *J* = 7.8 Hz, 1H), 2.61(q, *J* = 10.8 Hz, 1H), 3.57(td, *J_1_* = 9.0 Hz, *J_2_* = 18.0 Hz, 1H, H-6), 3.76(t, *J_1_* = 7.2 Hz, *J_2_* = 14.4 Hz, 1H, H-24), 4.71(q, *J* = 7.2 Hz, 1H, H-16), 4.86(dd, *J_1_* = 4.2 Hz, *J_2_* = 10.8 Hz, 1H, H-3), 6.89(t, *J_1_* = 7.2 Hz, *J_2_* = 14.4 Hz, 1H, AR-H), 6.99(d, *J* = 8.4 Hz, 1H, AR-H), 7.45(t, *J_1_* = 7.8 Hz, *J_2_* = 15.0 Hz, 1H, AR-H), 7.86(d, *J* = 7.8 Hz, 1H, AR-H), 10.92(s, 1H, AR-H); ^13^C-NMR (CDCl_3_, 150 MHz, ppm): δ 170.03(-C=O), 161.88, 135.67, 129.88, 119.25,117.74, 113.11(AR-C), 87.20(C-20), 81.51(C-24), 81.72(C-3), 73.84(C-16), 71.98(C-25), 68.97(C-6), 57.61(C-17), 53.92(C-5), 47.30(C-8), 46.62(C-14), 46.11(C-15), 45.12(C-13), 40.91(C-4), 38.21(C-7), 34.55(C-22), 33.05(C-1), 31.85(C-12), 31.65(C-19), 29.43(C-2), 28.46(C-10), 27.96(C-28), 27.86(C-21), 26.95(C-11), 26.62(C-27), 26.04(C-26), 25.80(C-23), 21.64(C-9), 21.04(C-18), 20.22(C-30), 16.77(C-29); ESI-HRMS (m/z): calcd for C_37_H_54_O_7_Na^+^ [M+Na]^+^: 633.3767, found: 633.3771.


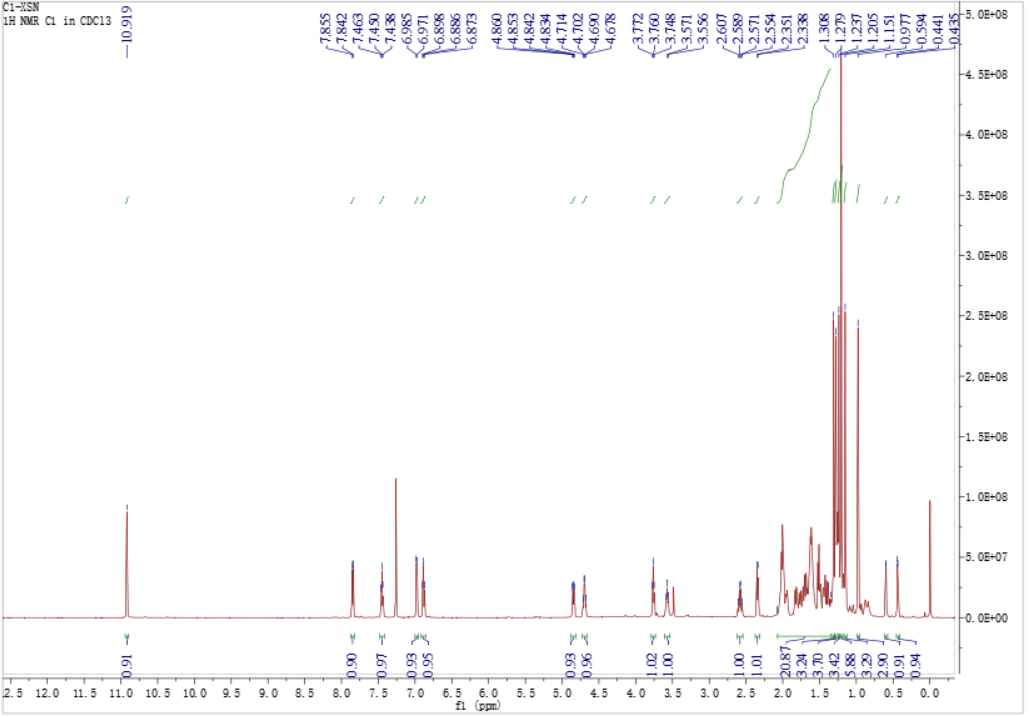


Figure.S37. ^1^H-NMR of compound C1


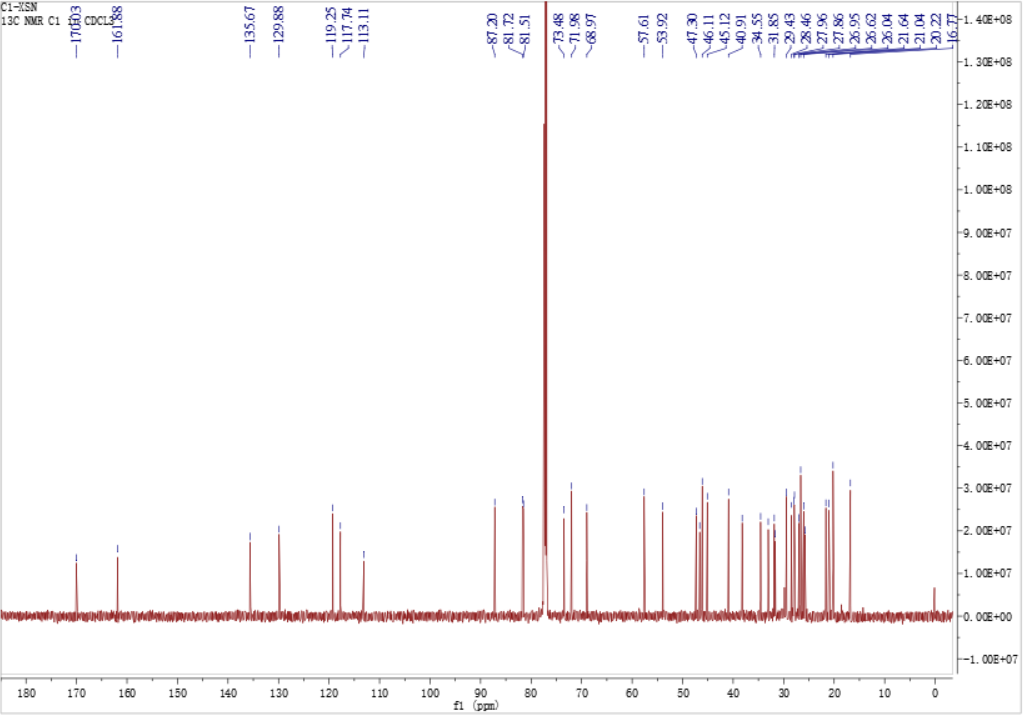


Figure.S38. ^13^C-NMR of compound C1


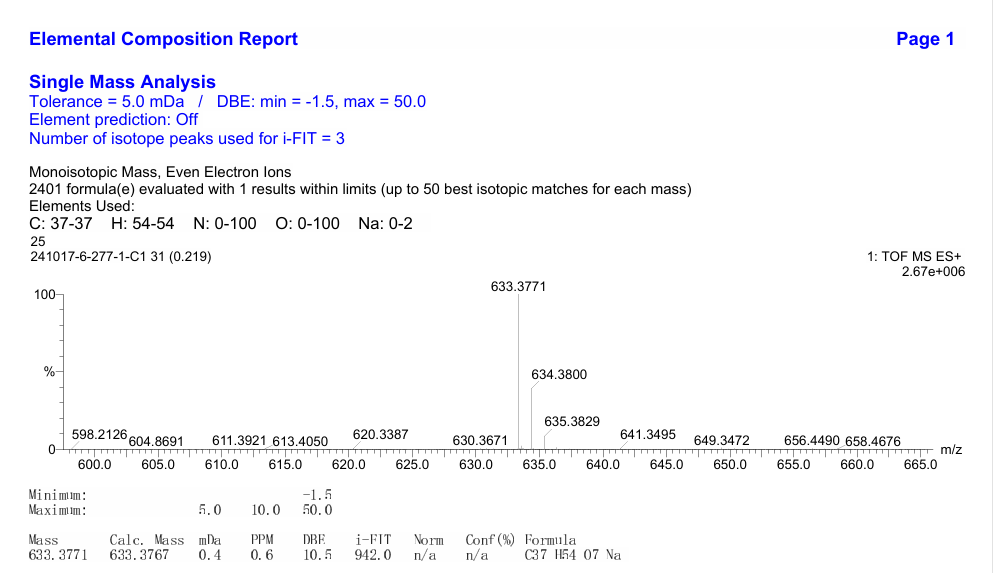


Figure.S39. HRMS of compound C1

### Compound C2

White powder, melting point: 246-248℃. ^1^H-NMR (CDCl_3_, 600 MHz, ppm): δ 0.44(d, *J* = 4.2 Hz, 1H, H-19b), 0.60(d, *J* = 4.2 Hz, 1H, H-19a), 0.98, 1.15, 1.24, 1.28, 1.31(s, 3H, -CH_3_×5), 1.20(s, 3H, -CH_3_×2), 2.27(s, 3H, -CH_3_, AR-CH_3_), 2.35(d, *J* = 7.8 Hz, 1H), 2.61(q, *J* = 10.2 Hz, 1H), 3.57(td, *J_1_* = 2.4 Hz, *J_2_* = 9.6 Hz, 1H, H-6), 3.76(t, *J_1_* = 7.8 Hz, *J_2_* = 15.0 Hz, 1H, H-24), 4.71(q, *J* = 7.2 Hz, 1H, H-16), 4.86(dd, *J_1_* = 4.2 Hz, *J_2_* = 10.8 Hz, 1H, H-3), 6.79(t, *J_1_* = 7.8 Hz, *J_2_* = 15.6 Hz, 1H, AR-H), 7.32(d, *J* = 7.2 Hz, 1H, AR-H), 7.71(d, *J* = 8.4 Hz, 1H, AR-H), 11.17(s, 1H, AR-OH); ^13^C-NMR (CDCl_3_, 150 MHz, ppm): δ 170.47(-C=O), 160.30, 136.41, 127.39, 126.78, 118.57, 112.37(AR-C), 87.22(C-20), 81.53(C-24), 81.59(C-3), 73.48(C-16), 71.98(C-25), 68.99(C-6), 57.61(C-17), 53.96(C-5), 47.25(C-8), 46.61(C-14), 46.12(C-15), 45.14(C-13), 40.92(C-4), 38.19(C-7), 34.56(C-22), 33.08(C-1), 31.86(C-12), 31.62(C-19), 29.43(C-2), 28.44(C-10), 27.92(C-28), 27.87(C-21), 26.94(C-11), 26.63(C-27), 26.03(C-26), 25.76(C-23), 21.65(C-9), 21.05(C-18), 20.22(C-30), 16.77(C-29), 15.83(AR-CH_3_); ESI-HRMS (m/z): calcd for C_38_H_56_O_7_Na^+^ [M+Na]^+^: 647.3924, found: 647.3927.


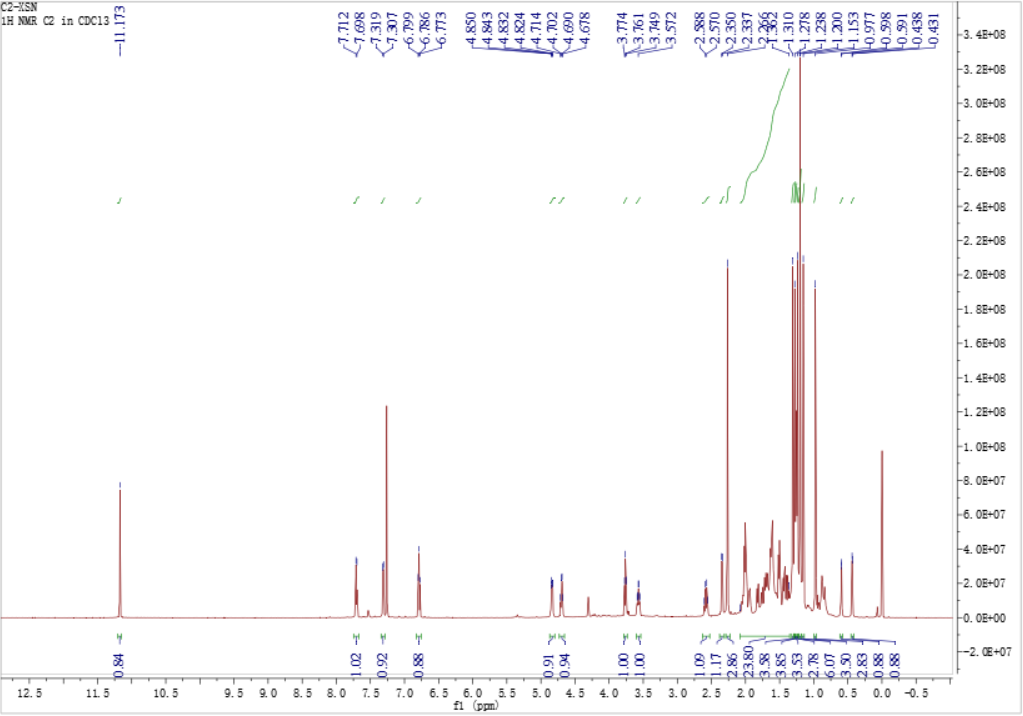


Figure.S40. ^1^H-NMR of compound C2


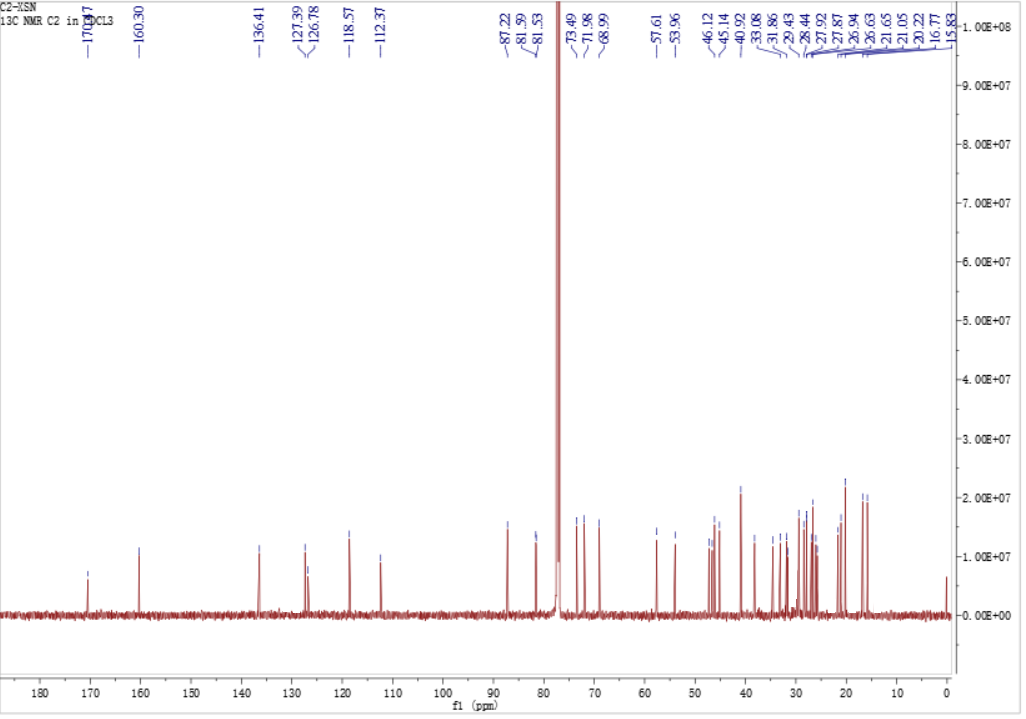


Figure.S41. ^13^C-NMR of compound C2


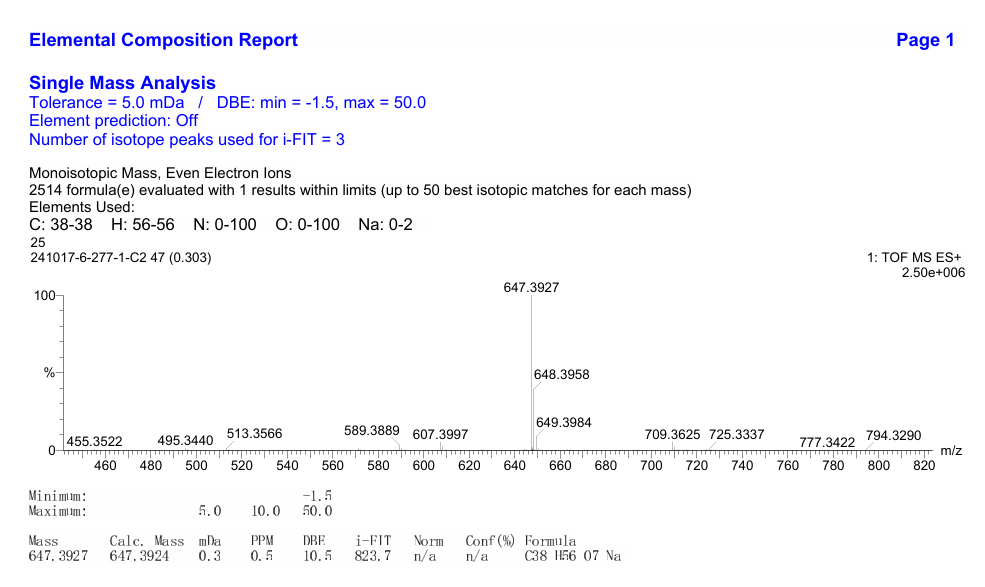


Figure.S42. HRMS of compound C2

### Compound C3

White powder, melting point: 238-240℃. ^1^H-NMR (CDCl_3_, 600 MHz, ppm): δ 0.44(d, *J* = 4.2 Hz, 1H, H-19b), 0.60(d, *J* = 4.2 Hz, 1H, H-19a), 0.98, 1.16, 1.24, 1.28, 1.31(s, 3H, -CH_3_×5), 1.20(s, 3H, -CH_3_×2), 2.34(s, 3H, AR-CH_3_), 2.60(q, *J* = 9.6 Hz, 1H), 3.57(td, *J_1_* = 3.0 Hz, *J_2_* = 9.6 Hz, 1H, H-6), 3.77(t, *J_1_* = 7.2 Hz, *J_2_* = 15.0 Hz, 1H, H-24), 4.71(q, *J* = 7.8 Hz, 1H, H-16), 4.84(dd, *J_1_* = 4.2 Hz, *J_2_* = 10.8 Hz, 1H, H-3), 6.70(d, *J* = 7.8 Hz, 1H, AR-H), 6.79(s, 1H, AR-H), 7.73(d, *J* = 7.8 Hz, 1H, AR-H), 10.86(s, 1H, AR-OH); ^13^C-NMR (CDCl_3_, 150 MHz, ppm): δ 170.04(-C=O), 161.85, 146.98, 129.69, 120.52, 117.87, 112.15, 110.52(AR-C), 87.23(C-20), 81.40(C-24), 81.54(C-3), 73.49(C-16), 71.99(C-25), 69.02(C-6), 57.61(C-17), 53.98(C-5), 47.22(C-8), 46.85(C-14), 46.13(C-15), 45.17(C-13), 40.91(C-4), 38.18(C-7), 34.56(C-22), 33.09(C-1), 31.85(C-12), 31.59(C-19), 29.42(C-2), 28.44(C-10), 27.89(C-28), 27.36(C-21), 26.97(C-11), 26.63(C-27), 26.03(C-26), 25.72(C-23), 22.00(AR-CH_3_), 21.65(C-9), 21.05(C-18), 20.21(C-30), 16.77(C-29); ESI-HRMS (m/z): calcd for C_38_H_56_O_7_Na^+^ [M+Na]^+^: 647.3924, found: 647.3920.


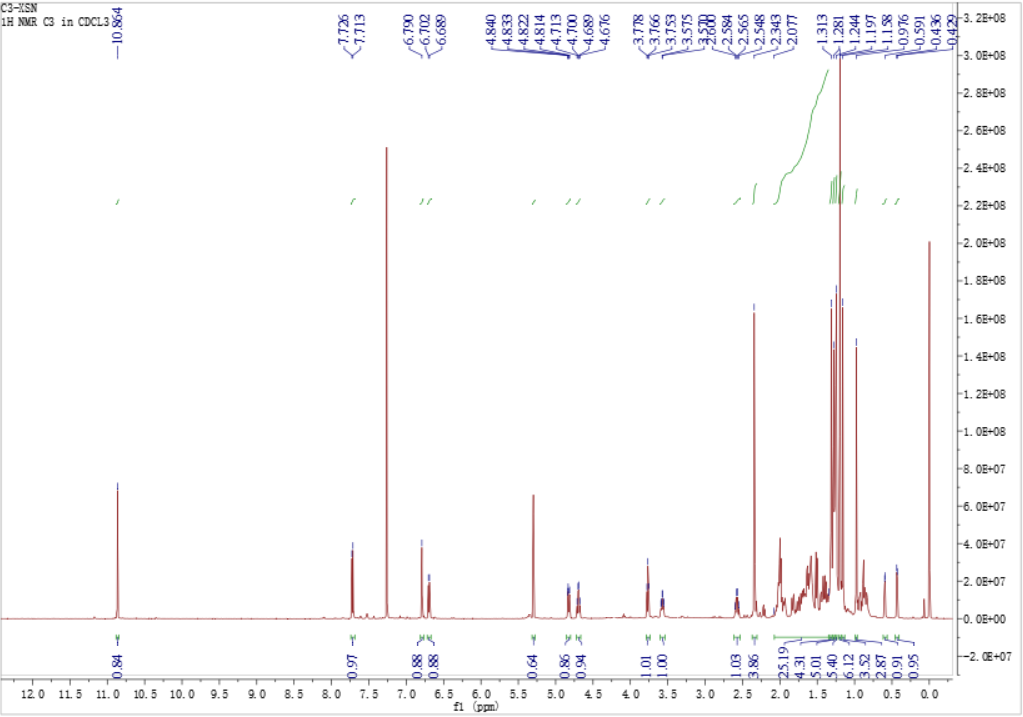


Figure.S43. ^1^H-NMR of compound C3


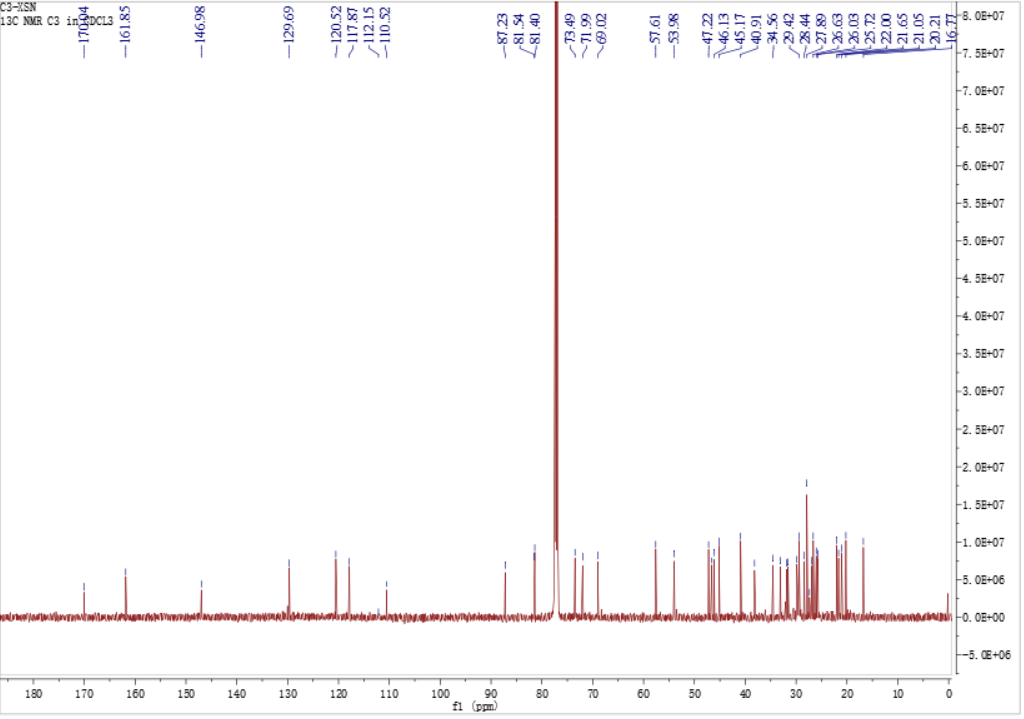


Figure.S44. ^13^C-NMR of compound C3


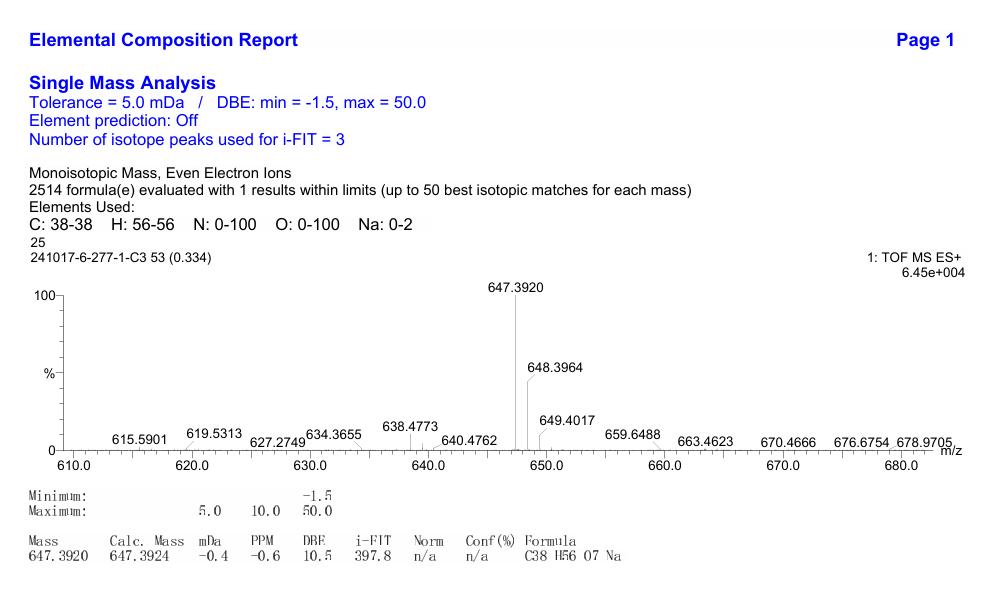


Figure.S45. HRMS of compound C3

### Compound C4

White powder, melting point: 241-243℃. ^1^H-NMR (CDCl_3_, 600 MHz, ppm): δ 0.44(d, *J* = 4.2 Hz, 1H, H-19b), 0.60(d, *J* = 4.2 Hz, 1H, H-19a), 0.98, 1.15, 1.21, 1.24, 1.25, 1.28, 1.31(s, 3H, -CH_3_×7), 2.30(s, 3H, AR-CH_3_), 2.35(d, *J* = 7.8 Hz, 1H), 2.60(q, *J* = 10.2 Hz, 1H), 3.60(td, *J_1_* = 2.4 Hz, *J_2_* = 9.6 Hz, 1H, H-6), 3.77(t, *J_1_* = 7.8 Hz, *J_2_* = 14.4 Hz, 1H, H-24), 4.71(q, *J* = 7.2 Hz, 1H, H-16), 4.86(dd, *J_1_* = 4.2 Hz, *J_2_* = 10.8 Hz, 1H, H-3), 6.88(d, *J* = 8.4 Hz, 1H, AR-H), 7.53(q, *J* = 3.6 Hz, 1H, AR-H), 7.61(s, 1H, AR-H), 10.74(s, 1H, AR-OH); ^13^C-NMR (CDCl_3_, 150 MHz, ppm): δ 169.92(-C=O), 159.66, 136.52, 132.46, 130.90, 128.82, 117.39(AR-C), 87.08(C-20), 81.39(C-24), 81.39(C-3), 73.35(C-16), 71.84(C-25), 68.86(C-6), 57.48(C-17), 53.84(C-5), 47.15(C-8), 46.48(C-14), 45.99(C-15), 45.01(C-13), 40.08(C-4), 38.08(C-7), 34.43(C-22), 32.94(C-1), 31.74(C-12), 31.51(C-19), 29.72(C-2), 28.31(C-10), 27.80(C-28), 27.74(C-21), 26.85(C-11), 26.50(C-27), 25.90(C-26), 25.64(C-23), 21.53(C-9), 20.92(C-18), 20.52(AR-CH_3_), 20.09(C-30), 16.70(C-29); ESI-HRMS (m/z): calcd for C_38_H_56_O_7_Na^+^ [M+Na]^+^: 647.3924, found: 647.3928.


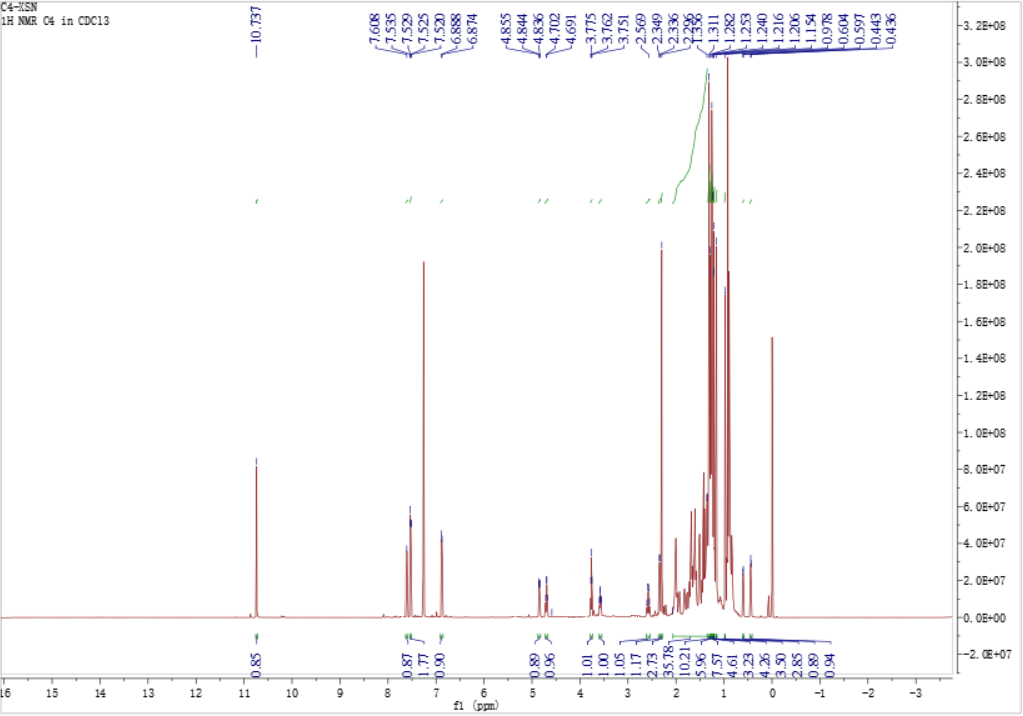


Figure.S46. ^1^H-NMR of compound C4


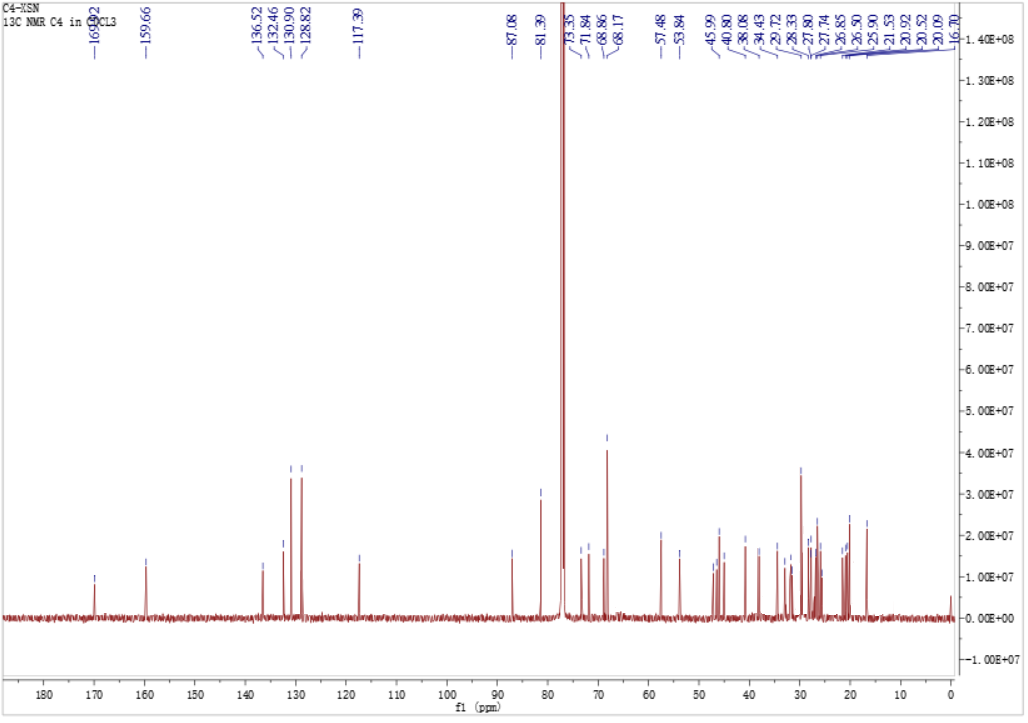


Figure.S47. ^13^C-NMR of compound C4


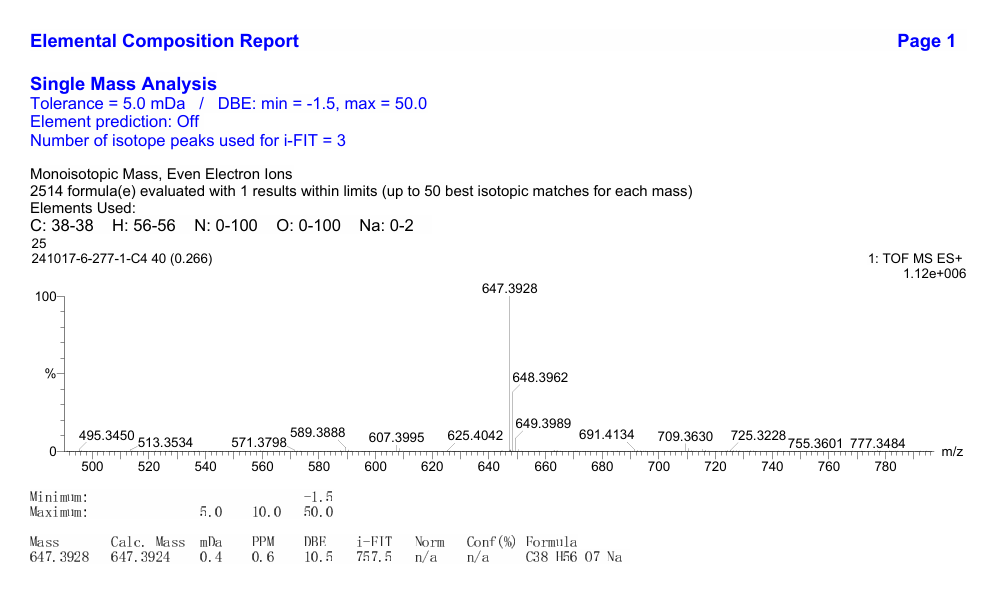


Figure.S48. HRMS of compound C4

### Compound C5

White powder, melting point: 226-228℃. ^1^H-NMR (CDCl_3_, 600 MHz, ppm): δ 0.44(d, *J* = 4.2 Hz, 1H, H-19b), 0.61(d, *J* = 4.2 Hz, 1H, H-19a), 0.98, 1.16, 1.20, 1.25, 1.28, 1.31, 1.34(s, 3H, -CH_3_×7), 2.35(d, *J* = 7.8 Hz, 1H), 2.60(q, *J* = 10.2 Hz, 1H), 3.59(td, *J_1_* = 3.0 Hz, *J_2_* = 9.6 Hz, 1H, H-6), 3.78(t, *J_1_* = 7.8 Hz, *J_2_* = 15.0 Hz, 1H, H-24), 4.71(q, *J* = 7.8 Hz, 1H, H-16), 4.91(dd, *J_1_* = 4.2 Hz, *J_2_* = 11.4 Hz, 1H, H-3), 6.97-7.02(m, 1H, AR-H), 8.11-8.16(m, 2H, AR-H), 12.19(s, 1H, AR-OH); ^13^C-NMR (CDCl_3_, 150 MHz, ppm): δ 170.00(-C=O), 167.91, 156.06, 132.59, 131.03, 128.95, 118.42(AR-C), 87.21(C-20), 81.53(C-24), 83.31(C-3), 73.46(C-16), 72.00(C-25), 68.95(C-6), 57.61(C-17), 53.85(C-5), 47.20(C-8), 46.55(C-14), 46.11(C-15), 45.17(C-13), 40.91(C-4), 38.28(C-7), 34.56(C-22), 33.06(C-1), 31.80(C-12), 31.75(C-19), 29.85(C-2), 28.46(C-10), 27.90(C-28), 27.58(C-21), 26.87(C-11), 26.65(C-27), 26.04(C-26), 25.69(C-23), 21.66(C-9), 21.14(C-18), 20.22(C-30), 16.71(C-29); ESI-HRMS (m/z): calcd for C_37_H_53_NO_9_Na^+^ [M+Na]^+^: 678.3618, found: 678.3619.


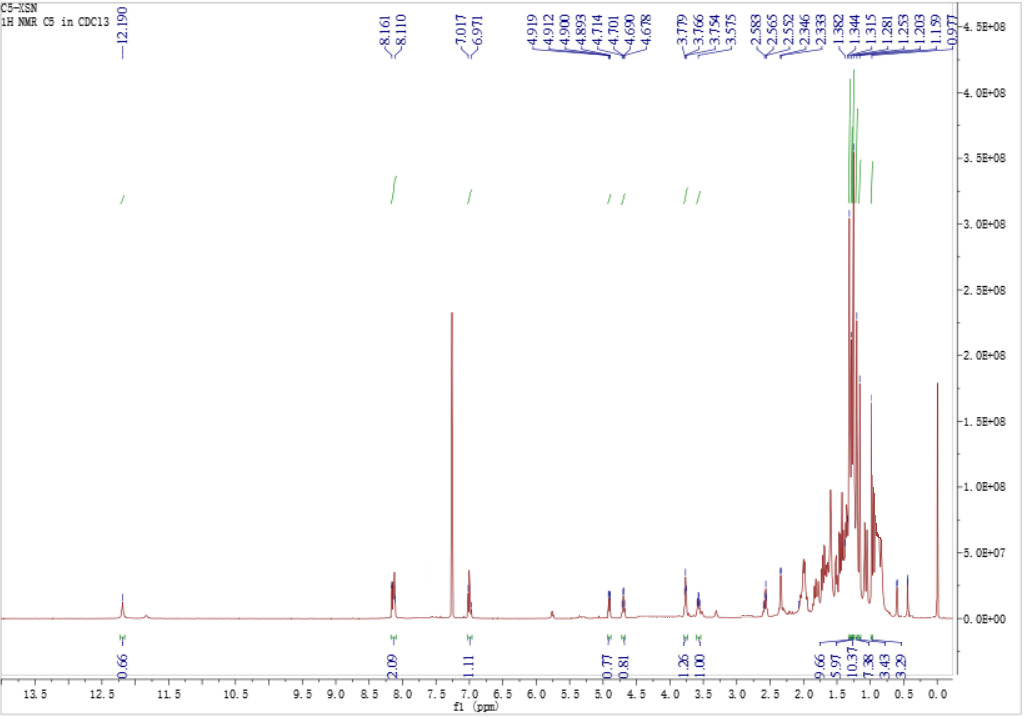


Figure.S49. 1H-NMR of compound C5


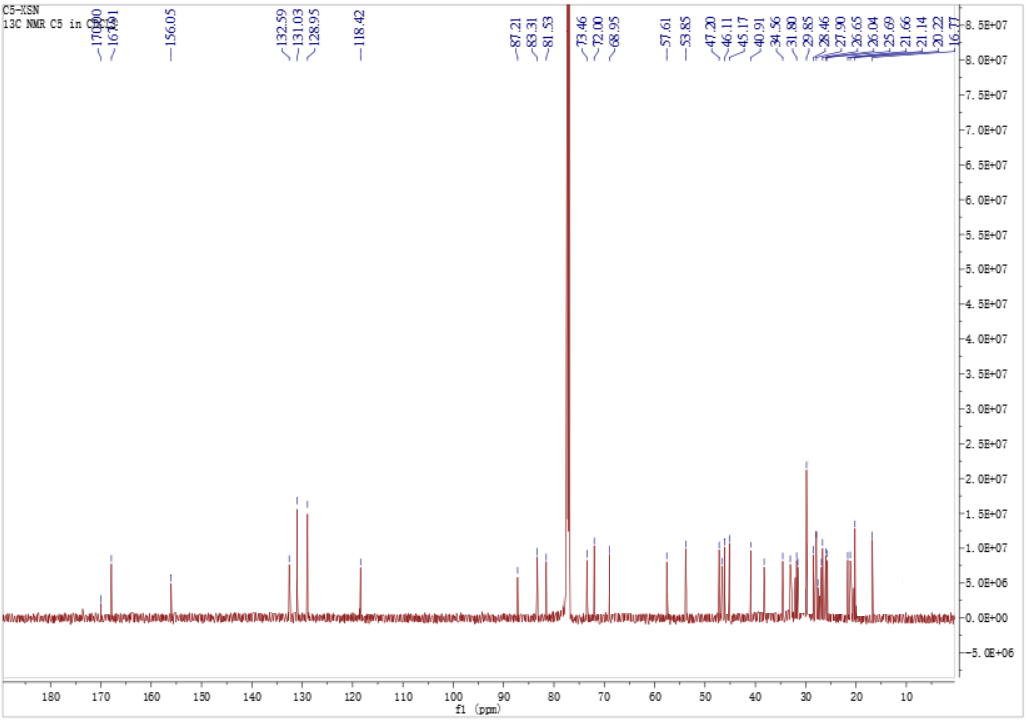


Figure.S50. ^13^C-NMR of compound C5


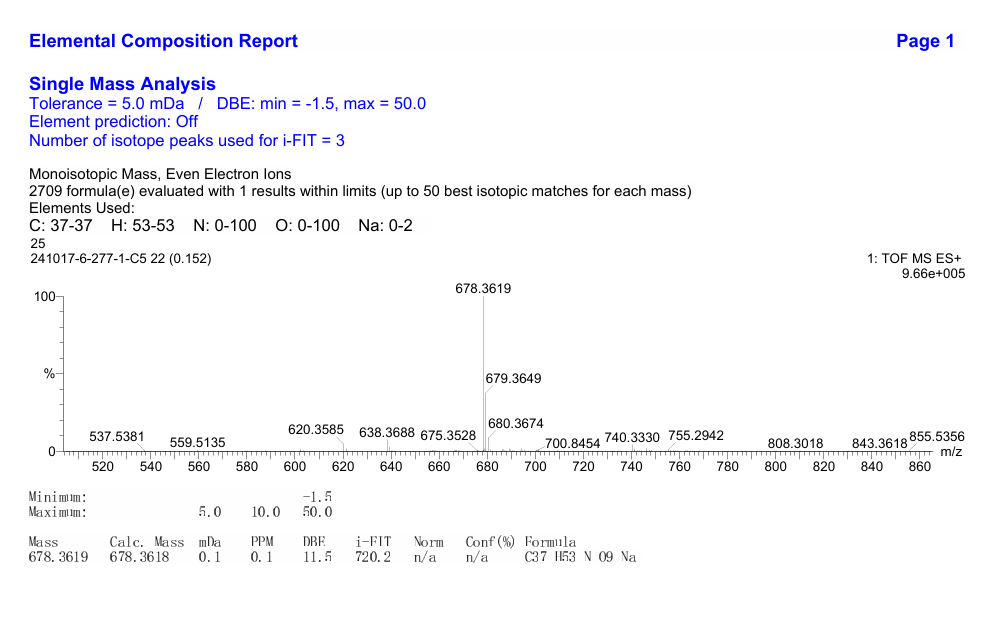


Figure.S51. HRMS of compound C5

### Compound C6

White powder, melting point: 232-234℃. ^1^H-NMR (CDCl_3_, 600 MHz, ppm): δ 0.45(d, *J* = 4.2 Hz, 1H, H-19b), 0.61(d, *J* = 4.2 Hz, 1H, H-19a), 0.98, 1.15, 1.20, 1.21, 1.23, 1.28, 1.31(s, 3H, -CH_3_×7), 2.35(d, *J* = 7.8 Hz, 1H), 2.60(q, *J* = 10.2 Hz, 1H), 3.59(td, *J_1_* = 3.6 Hz, *J_2_* = 10.2 Hz, 1H, H-6), 3.77(t, *J_1_* = 7.8 Hz, *J_2_* = 14.4 Hz, 1H, H-24), 4.71(q, *J* = 7.2 Hz, 1H, H-16), 4.92(dd, *J_1_* = 4.8 Hz, *J_2_* = 11.4 Hz, 1H, H-3), 7.71(dd, *J_1_* = 2.4 Hz, *J_2_* = 9.0 Hz, 1H, AR-H), 7.82(d, *J_1_* = 2.4 Hz, 1H, AR-H), 8.01(d, *J_1_* = 8.4 Hz, 1H, AR-H), 11.15(s, 1H, AR-OH); ^13^C-NMR (CDCl_3_, 150 MHz, ppm): δ 168.58(-C=O), 168.14, 152.04, 130.96, 117.78, 113.48, 113.08(AR-C), 87.05(C-20), 81.37(C-24), 83.07(C-3), 73.32(C-16), 71.87(C-25), 68.75(C-6), 57.48(C-17), 53.66(C-5), 47.15(C-8), 46.46(C-14), 45.96(C-15), 44.97(C-13), 40.79(C-4), 38.16(C-7), 34.43 (C-22), 32.89 (C-1), 31.68 (C-12), 31.53 (C-19), 29.21(C-2), 28.37 (C-10), 27.84 (C-28), 27.72 (C-21), 26.76 (C-11), 26.51 (C-27), 25.92 (C-26), 25.67(C-23), 21.51(C-9), 20.98(C-18), 20.09(C-30), 16.68(C-29); ESI-HRMS (m/z): calcd for C_37_H_53_NO_9_Na^+^ [M+Na]^+^: 678.3618, found: 678.3623.


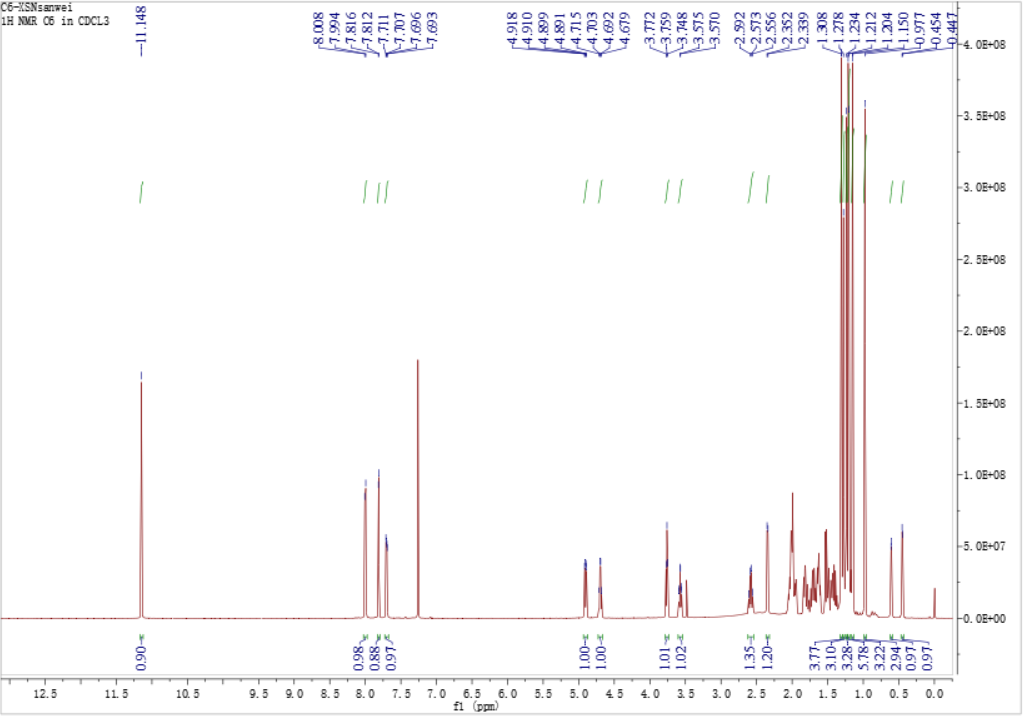


Figure.S52. ^1^H-NMR of compound C6


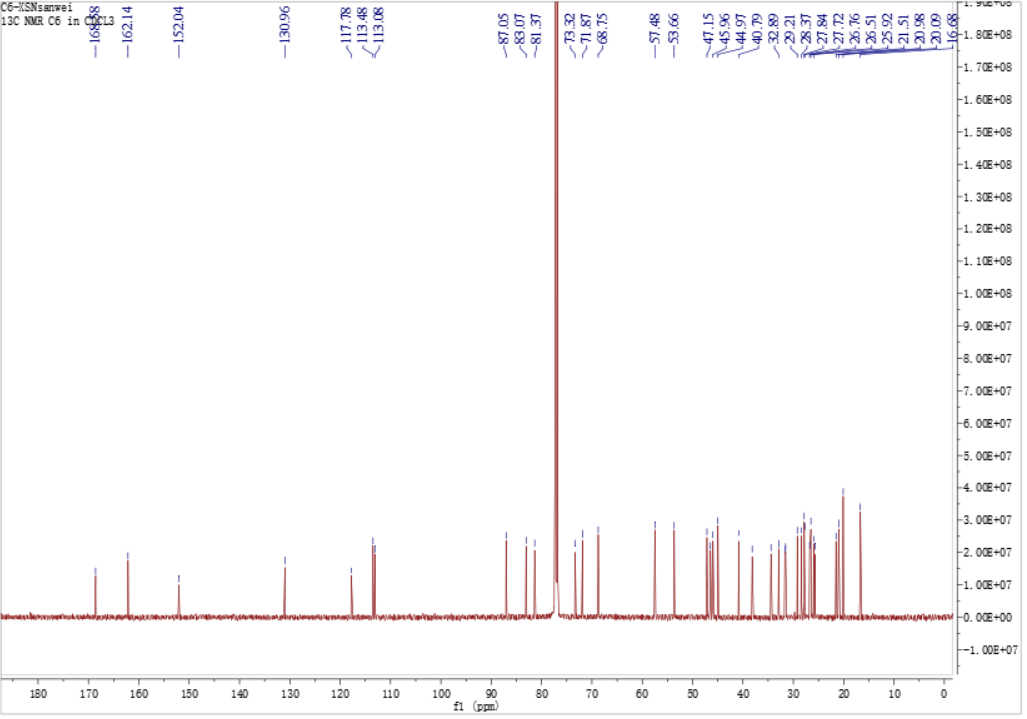


Figure.S53. ^13^C-NMR of compound C6


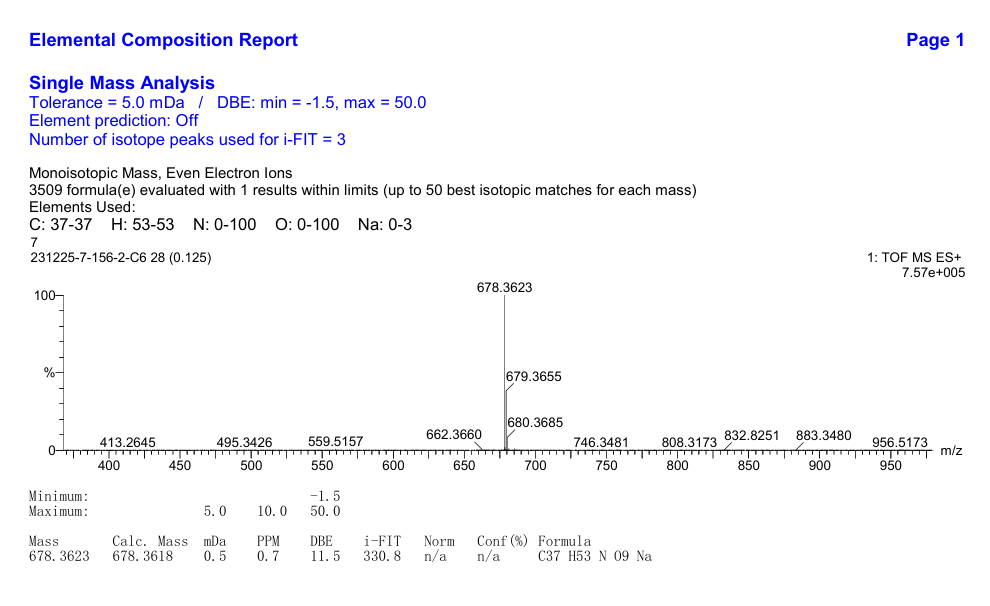


Figure.S54. HRMS of compound C6

### Compound C7

White powder, melting point: 240-242℃. ^1^H-NMR (CDCl_3_, 600 MHz, ppm): δ 0.47(d, *J* = 4.2 Hz, 1H, H-19b), 0.63(d, *J* = 4.2 Hz, 1H, H-19a), 0.98, 1.16, 1.21, 1.29, 1.31(s, 3H, -CH_3_×5), 1.24(s, 3H, -CH_3_×2), 2.35(d, *J* = 7.8 Hz, 1H), 2.61(q, *J* = 10.2 Hz, 1H), 3.59(td, *J_1_* = 3.0 Hz, *J_2_* = 9.6 Hz, 1H, H-6), 3.77(t, *J_1_* = 7.2 Hz, *J_2_* = 14.4 Hz, 1H, H-24), 4.71(q, *J* = 7.8 Hz, 1H, H-16), 4.95(dd, *J_1_* = 4.8 Hz, *J_2_* = 12.0 Hz, 1H, H-3), 7.09(d, *J_1_* = 9.0 Hz, 1H, AR-H), 8.34(dd, *J_1_* = 3.0 Hz, *J_2_* = 9.0 Hz, 1H, AR-H), 8.75(d, *J_1_* = 4.2 Hz, 1H, AR-H), 11.64(s, 1H, AR-OH); ^13^C-NMR (CDCl_3_, 150 MHz, ppm): δ 168.80(-C=O), 166.64, 140.14, 130.56, 126.50, 118.82, 112.83(AR-C), 87.22(C-20), 81.50(C-24), 83.32(C-3), 73.48(C-16), 72.02(C-25), 68.87(C-6), 57.62(C-17), 53.8(C-5), 47.17(C-8), 46.55(C-14), 46.10(C-15), 45.14(C-13), 40.97(C-4), 38.27(C-7), 34.56(C-22), 33.06(C-1), 31.83(C-12), 31.60(C-19), 29.85(C-2), 28.51(C-10), 27.92(C-28), 27.87(C-21), 26.95(C-11), 26.66(C-27), 26.05(C-26), 25.76(C-23), 21.66(C-9), 21.12(C-18), 20.22(C-30), 16.86(C-29); ESI-HRMS (m/z): calcd for C_37_H_53_NO_9_Na^+^ [M+Na]^+^: 678.3618, found: 678.3619.


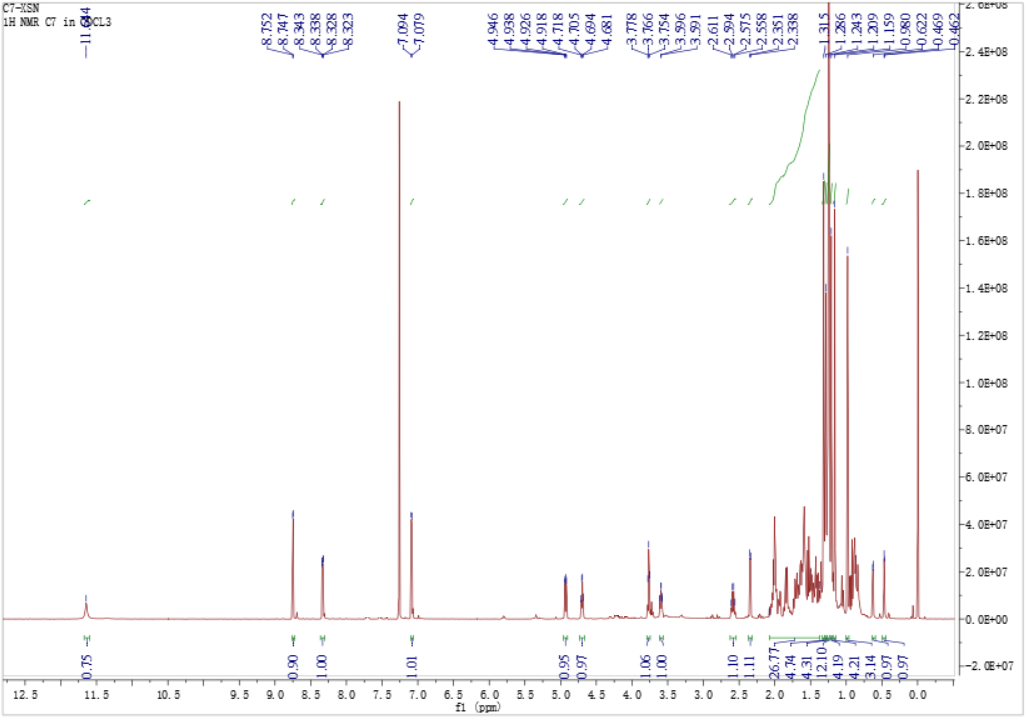


Figure.S55. ^1^H-NMR of compound C7


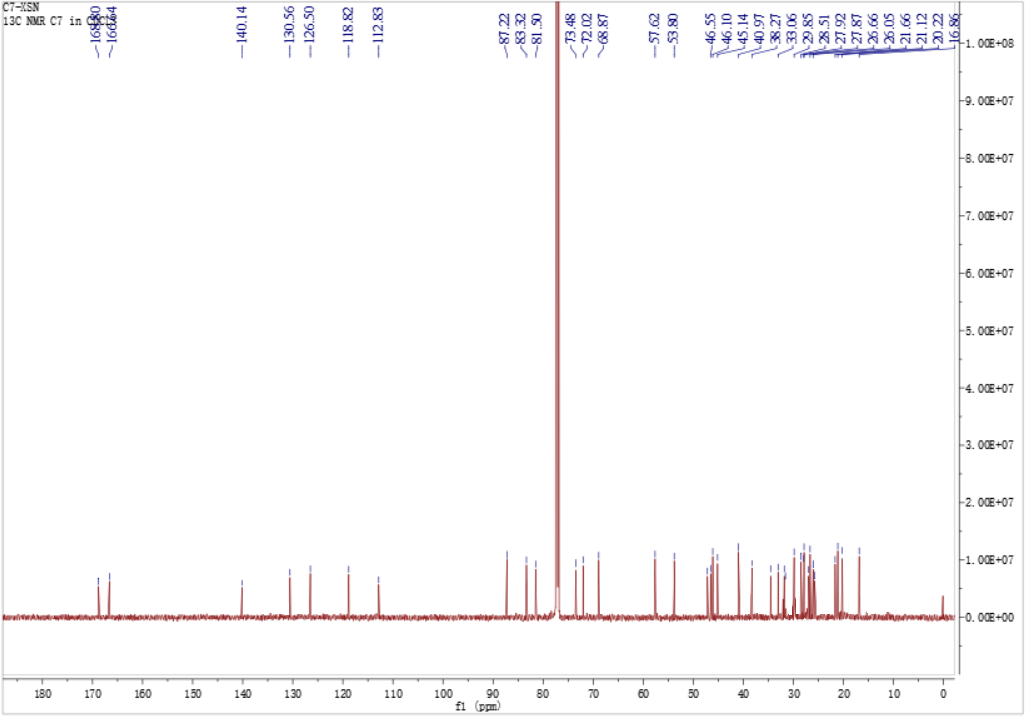


Figure.S56. ^13^C-NMR of compound C7


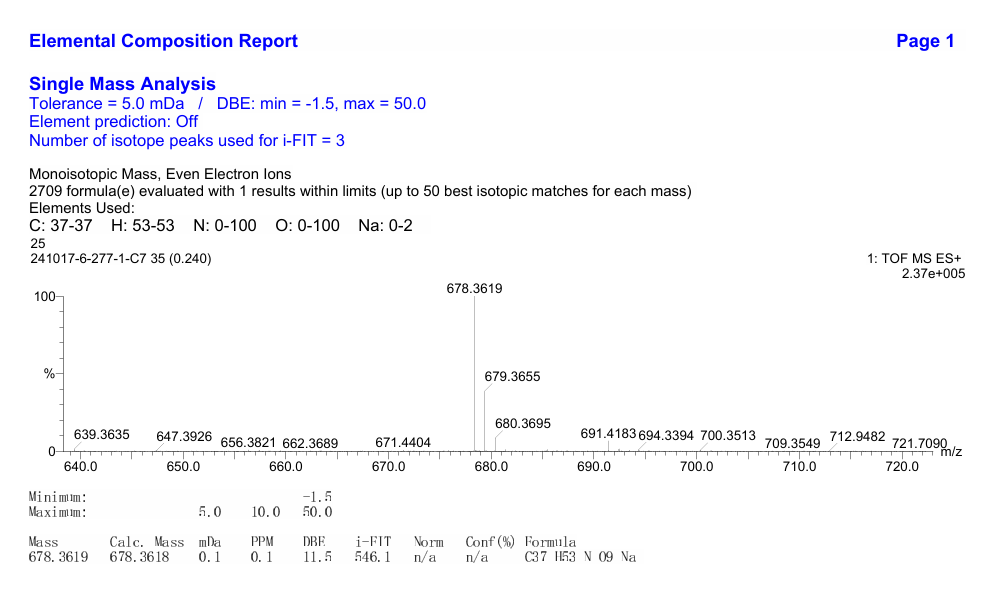


Figure.S57. HRMS of compound C7

### Compound C8

White powder, melting point: 242-244℃. ^1^H-NMR (CDCl_3_, 600 MHz, ppm): δ 0.47(d, *J* = 4.2 Hz, 1H, H-19b), 0.63(d, *J* = 3.0 Hz, 1H, H-19a), 0.97, 1.16, 1.21, 1.24, 1.25, 1.28, 1.32(s, 3H, -CH_3_×7), 3.55-3.62(m, 1H, H-6), 3.77(t, *J_1_* = 6.6 Hz, *J_2_* = 13.8 Hz, 1H, H-24), 4.71(q, *J* = 6.0 Hz, 1H, H-16), 5.00(dd, *J_1_* = 4.2 Hz, *J_2_* = 11.4 Hz, 1H, H-3), 7.52-7.53(m, 1H, AR-H), 7.70-7.72(m, 1H, AR-H), 9.02(s, 1H, AR-OH); ^13^C-NMR (CDCl_3_, 150 MHz, ppm): δ 167.87(-C=O), 132.46, 131.06, 128.99, 126.71(AR-C), 87.21(C-20), 81.51(C-24), 83.03(C-3), 73.45(C-16), 72.04(C-25), 68.83(C-6), 57.62(C-17), 53.72(C-5), 47.12(C-8), 46.49(C-14), 46.09(C-15), 45.16(C-13), 40.98(C-4), 38.33(C-7), 34.56(C-22), 33.05(C-1), 31.78(C-12), 31.56(C-19), 29.85(C-2), 29.17(C-10), 28.53(C-28), 27.8(C-21), 26.89(C-11), 26.67(C-27), 26.05(C-26), 25.70(C-23), 21.66(C-9), 21.21(C-18), 20.22(C-30), 16.85(C-29); ESI-HRMS (m/z): calcd for C_37_H_52_N_2_O_11_Na^+^ [M+Na]^+^: 723.3469, found: 723.3472.


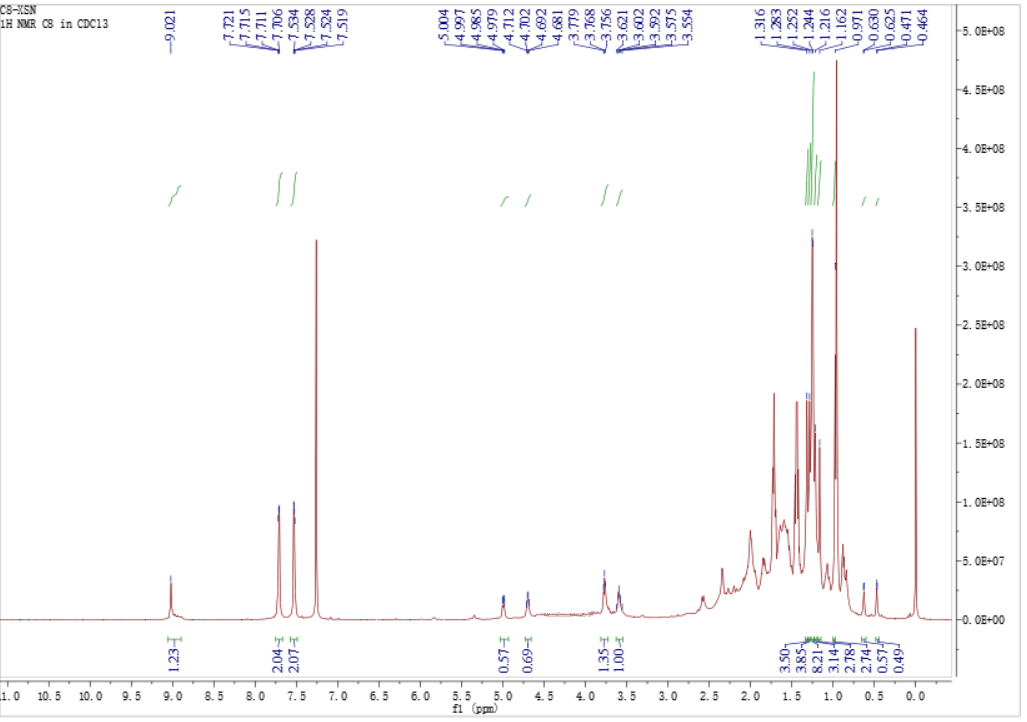


Figure.S58. ^1^H-NMR of compound C8


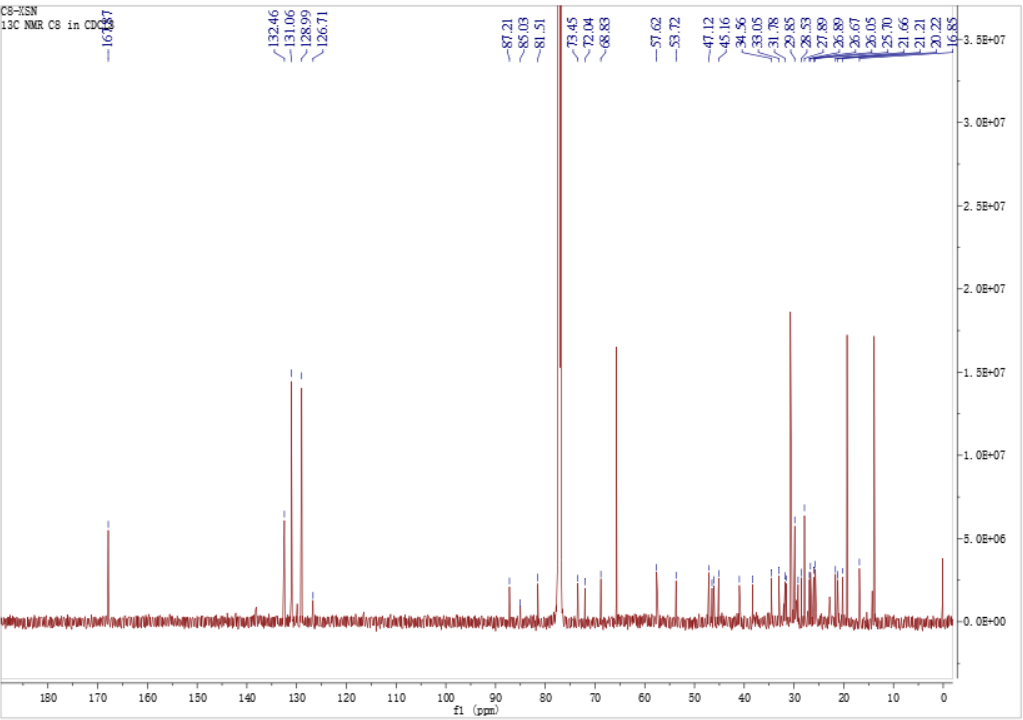


Figure.S59. ^13^C-NMR of compound C8


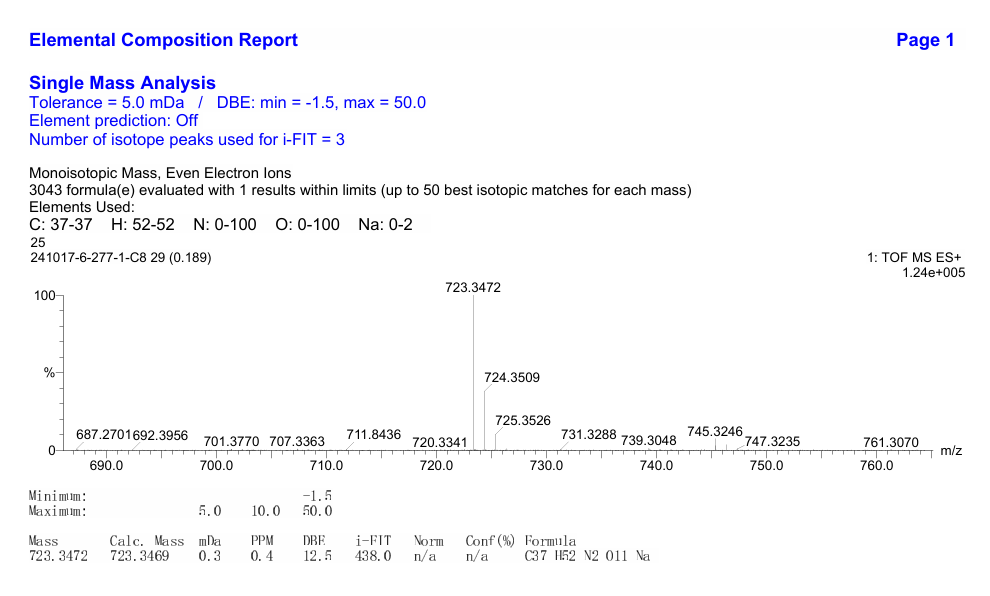


Figure.S60. HRMS of compound C8

### Compound C9

White powder, melting point: 240-242℃. ^1^H-NMR (CDCl_3_, 600 MHz, ppm): δ 0.44(d, *J* = 4.2 Hz, 1H, H-19b), 0.60(d, *J* = 3.6 Hz, 1H, H-19a), 0.98, 1.15, 1.24, 1.28, 1.31(s, 3H, -CH_3_×5), 0.19(s, 3H, -CH_3_×2), 2.35(d, *J* = 7.8 Hz, 1H), 2.61(q, *J* = 10.2 Hz, 1H), 3.58(t, *J_1_* = 8.4 Hz, *J_2_* = 16.8 Hz, 1H, H-6), 3.77(t, *J_1_* = 7.2 Hz, *J_2_* = 14.4 Hz, 1H, H-24), 4.71(q, *J* = 7.2 Hz, 1H, H-16), 4.85(dd, *J_1_* = 4.2 Hz, *J_2_* = 10.8 Hz, 1H, H-3), 6.60(t, *J_1_* = 6.6 Hz, *J_2_* = 15.0 Hz, 1H, AR-H), 6.68(d, *J* = 9.6 Hz, 1H, AR-H), 7.84(t, *J_1_* = 7.8 Hz, *J_2_* = 15.6 Hz, 1H, AR-H), 11.16(s, 1H, AR-OH); ^13^C-NMR (CDCl_3_, 150 MHz, ppm): δ 169.44(-C=O), 164.02, 132.03, 109.81, 107.48, 107.33, 104.63(AR-C), 87.20(C-20), 81.51(C-24), 81.92(C-3), 73.47(C-16), 71.98(C-25), 68.95(C-6), 57.61(C-17), 53.90(C-5), 47.29(C-8), 46.62(C-14), 46.11(C-15), 45.12(C-13), 40.90(C-4), 38.23(C-7), 34.56(C-22), 33.05(C-1), 31.84(C-12), 31.64(C-19), 29.41(C-2), 28.46(C-10), 27.96(C-28), 27.86(C-21), 26.95(C-11), 26.63(C-27), 26.04(C-26), 25.79(C-23), 21.64(C-9), 21.06(C-18), 20.22(C-30), 16.78(C-29); ESI-HRMS (m/z): calcd for C_37_H_53_O_7_FNa^+^ [M+Na]^+^: 651.3673, found: 651.3678.


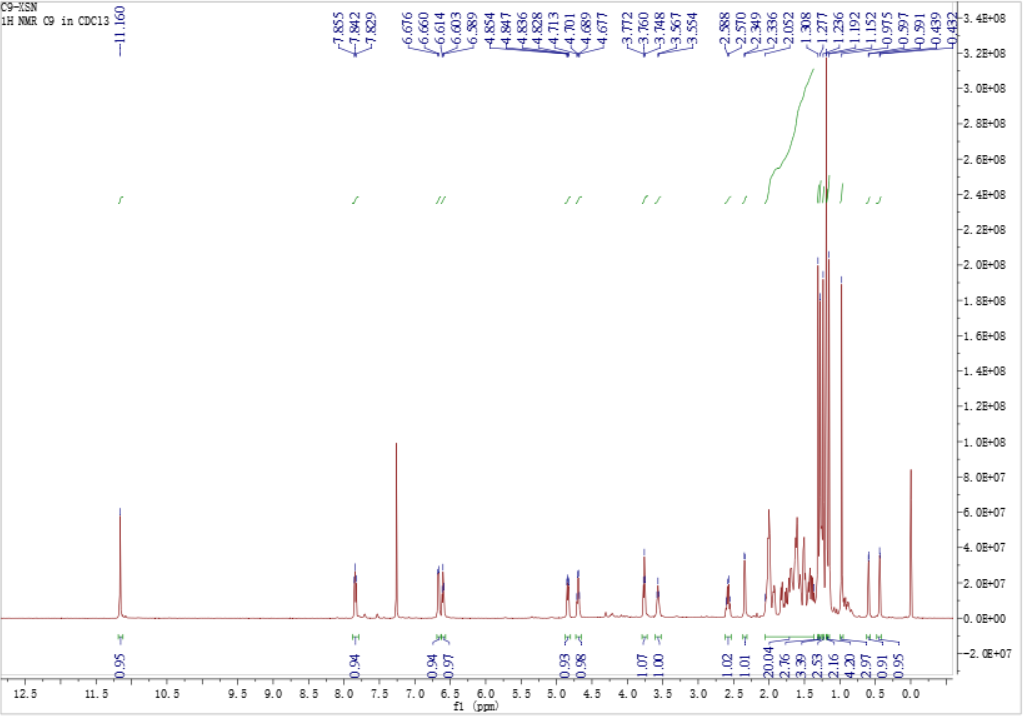


Figure.S61. ^1^H-NMR of compound C9


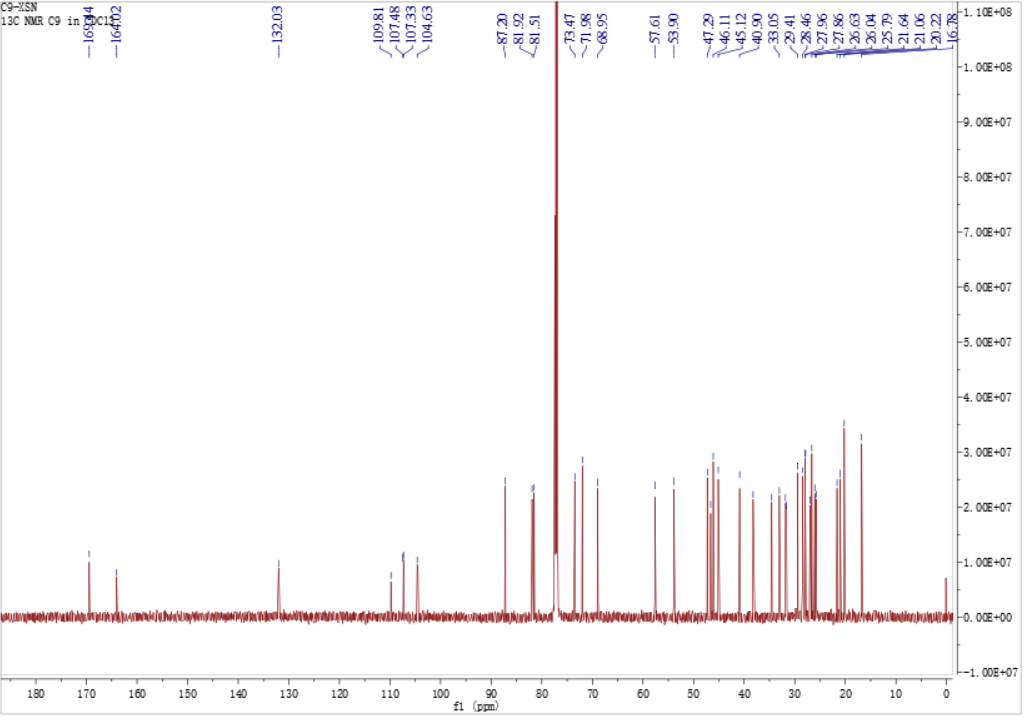


Figure.S62. ^13^C-NMR of compound C9


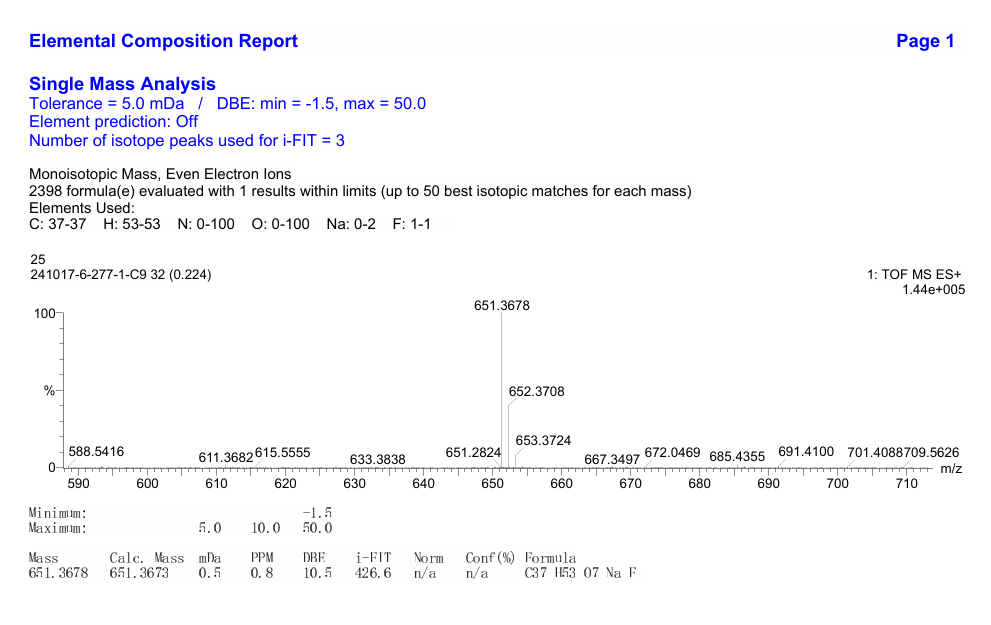


Figure.S63. HRMS of compound C9

### Compound C10

White powder, melting point: 237-239℃. ^1^H-NMR (CDCl_3_, 600 MHz, ppm): δ 0.44(d, *J* = 3.6 Hz, 1H, H-19b), 0.59(d, *J* = 3.6 Hz, 1H, H-19a), 0.97, 1.14, 1.23, 1.27, 1.30(s, 3H, -CH_3_×5), 1.19(s, 3H, -CH_3_×2), 2.35(d, *J* = 7.8 Hz, 1H), 2.61(q, *J* = 10.2 Hz, 1H), 3.57(t, *J_1_* = 9.0 Hz, *J_2_* = 16.8 Hz, 1H, H-6), 3.77(t, *J_1_* = 7.2 Hz, *J_2_* = 14.4 Hz, 1H, H-24), 4.71(q, *J* = 7.2 Hz, 1H, H-16), 4.85(dd, *J_1_* = 4.2 Hz, *J_2_* = 11.4 Hz, 1H, H-3), 6.87(dd, *J_1_* = 1.2 Hz, *J_2_* = 8.4 Hz, 1H, AR-H), 7.00(d, *J* = 1.2 Hz, 1H, AR-H), 7.76(d, *J* = 9.0 Hz, 1H, AR-H), 11.02(s, 1H, AR-OH); ^13^C-NMR (CDCl_3_, 150 MHz, ppm): δ 169.48(-C=O), 162.44, 141.39, 130.85, 119.94, 117.92, 111.73(AR-C), 87.19(C-20), 81.51(C-24), 82.10(C-3), 73.47(C-16), 71.99(C-25), 68.94(C-6), 57.62(C-17), 53.88(C-5), 47.30(C-8), 46.63(C-14), 46.11(C-15), 45.12(C-13), 40.91(C-4), 38.24(C-7), 34.56(C-22), 33.04(C-1), 31.83(C-12), 31.65(C-19), 29.40(C-2), 28.47(C-10), 27.97(C-28), 27.86(C-21), 26.94(C-11), 26.64(C-27), 26.04(C-26), 25.81(C-23), 21.65(C-9), 21.06(C-18), 20.22(C-30), 16.78(C-29); ESI-HRMS (m/z): calcd for C_37_H_53_O_7_ClNa^+^ [M+Na]^+^: 667.3378, found: 667.3383.


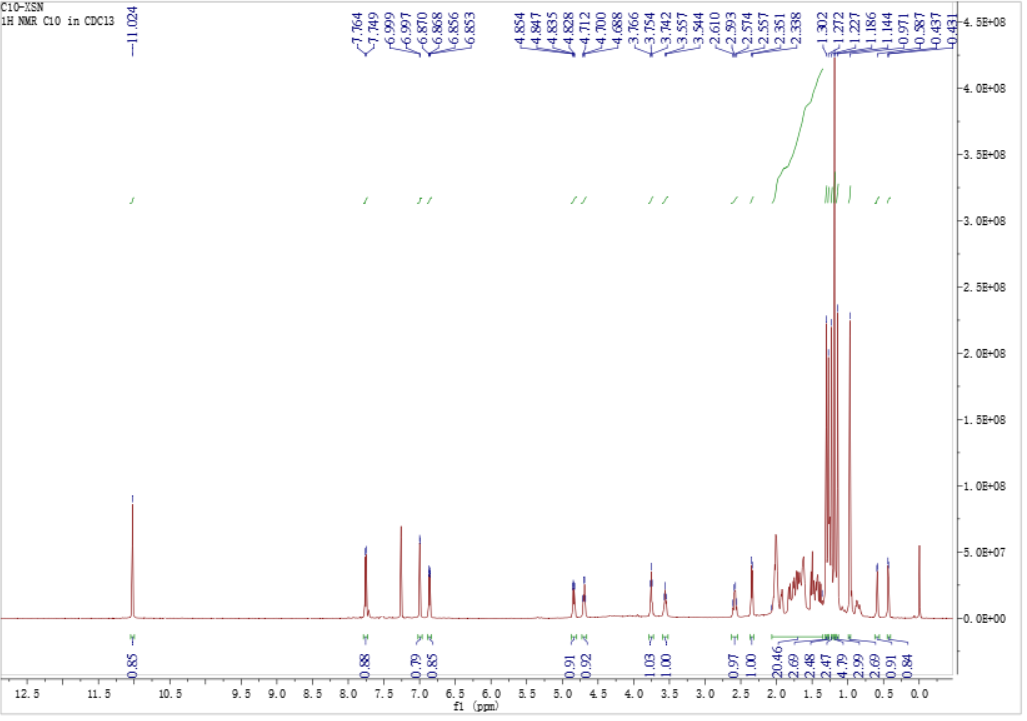


Figure.S64. ^1^H-NMR of compound C10


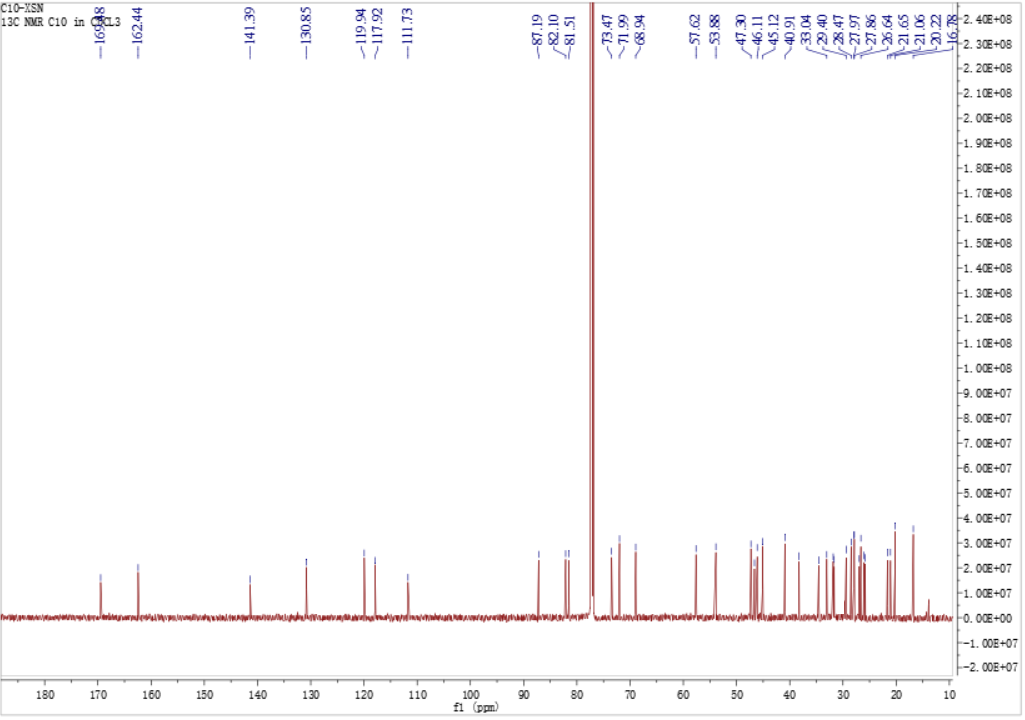


Figure.S65. ^13^C-NMR of compound C10


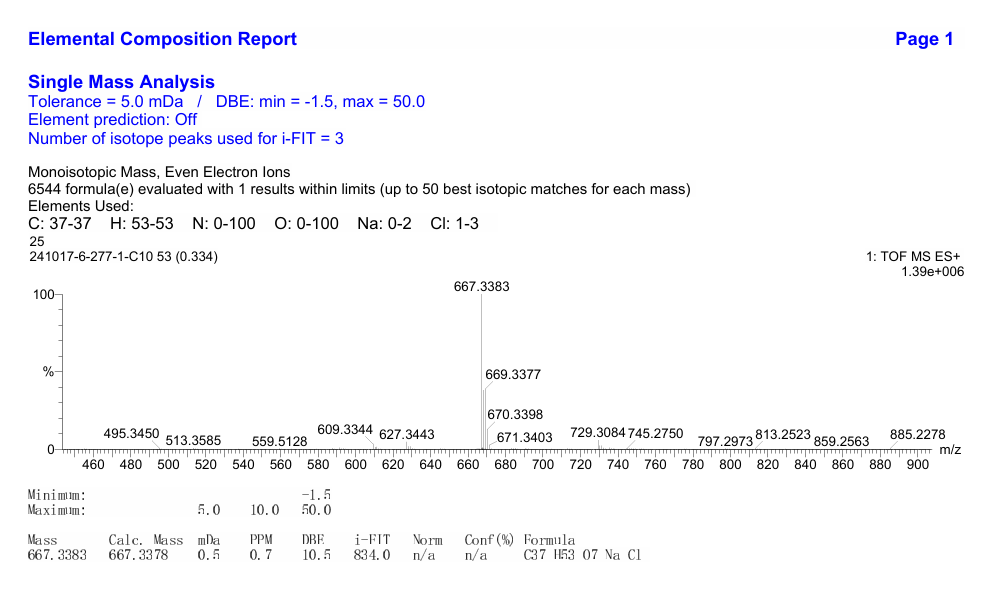


Figure.S66. HRMS of compound C10

### Compound C11

White powder, melting point: 243-245℃. ^1^H-NMR (CDCl_3_, 600 MHz, ppm): δ 0.44(d, *J* = 4.2 Hz, 1H, H-19b), 0.59(d, *J* = 4.2 Hz, 1H, H-19a), 0.97, 1.14, 1.23, 1.27, 1.30(s, 3H, -CH_3_×5), 1.18(s, 3H, -CH_3_×2), 2.35(d, *J* = 7.8 Hz, 1H), 2.61(q, *J* = 10.2 Hz, 1H), 3.57(t, *J_1_* = 7.8 Hz, *J_2_* = 16.8 Hz, 1H, H-6), 3.77(t, *J_1_* = 7.2 Hz, *J_2_* = 14.4 Hz, 1H, H-24), 4.71(q, *J* = 7.2 Hz, 1H, H-16), 4.85(dd, *J_1_* = 4.2 Hz, *J_2_* = 10.8 Hz, 1H, H-3), 7.03(d, *J* = 8.4 Hz, 1H, AR-H), 7.17-7.26(m, 1H, AR-H), 7.69(d, *J* = 8.4 Hz, 1H, AR-H), 10.99(s, 1H, AR-OH); ^13^C-NMR (CDCl_3_, 150 MHz, ppm): δ 169.59(-C=O), 162.30, 130.86, 129.87, 122.82, 121.00, 112.11(AR-C), 87.18(C-20), 81.50(C-24), 82.15(C-3), 73.47(C-16), 71.98(C-25), 68.91(C-6), 57.61(C-17), 53.85(C-5), 47.32(C-8), 46.63(C-14), 46.10(C-15), 45.09(C-13), 40.90(C-4), 38.24(C-7), 34.55(C-22), 33.03(C-1), 31.82(C-12), 31.67(C-19), 29.40(C-2), 28.47(C-10), 27.99(C-28), 27.84(C-21), 26.92(C-11), 26.63(C-27), 26.04(C-26), 25.83(C-23), 21.64(C-9), 21.05(C-18), 20.22(C-30), 16.78(C-29); ESI-HRMS (m/z): calcd for C_37_H_53_O_7_BrNa^+^ [M+Na]^+^: 711.2872, found: 711.2875.


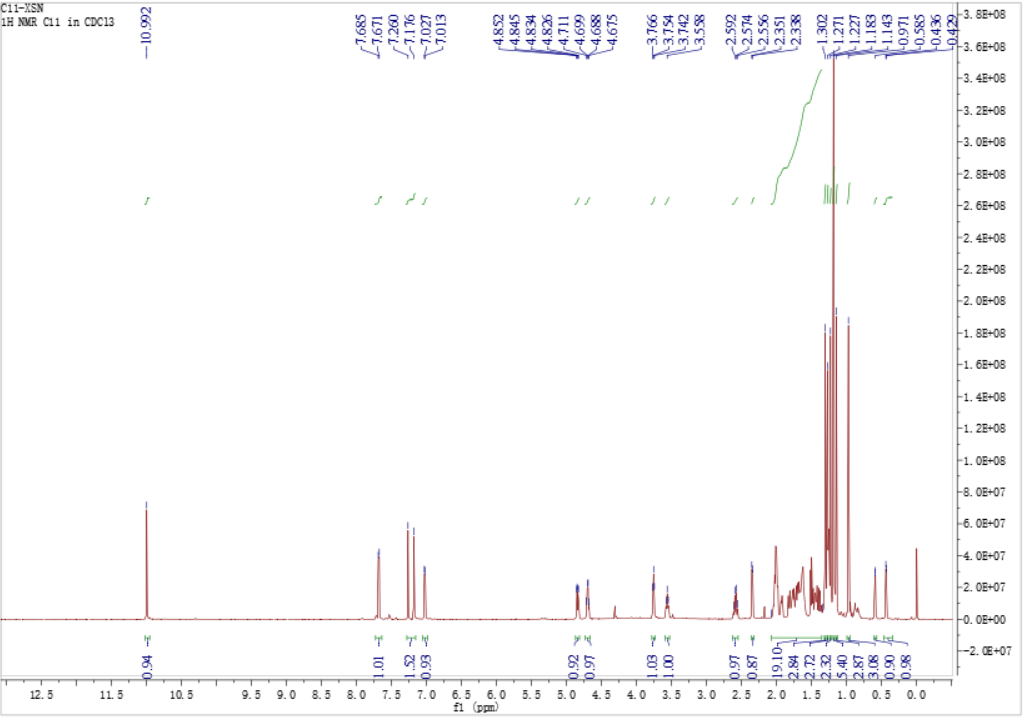


Figure.S67. ^1^H-NMR of compound C11


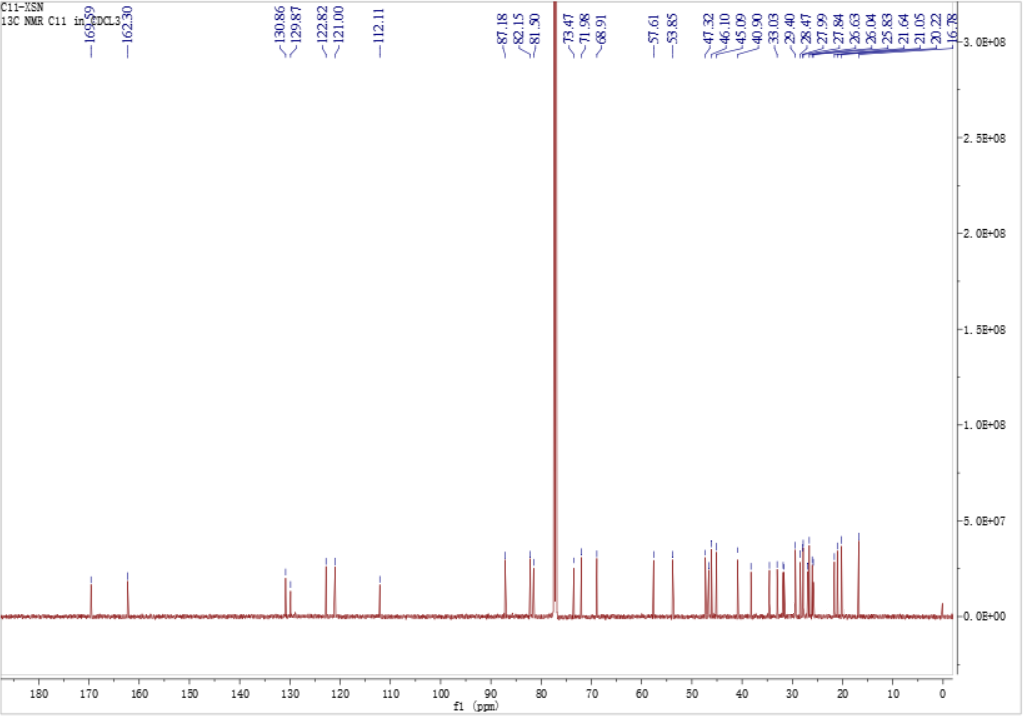


Figure.S68. ^13^C-NMR of compound C11


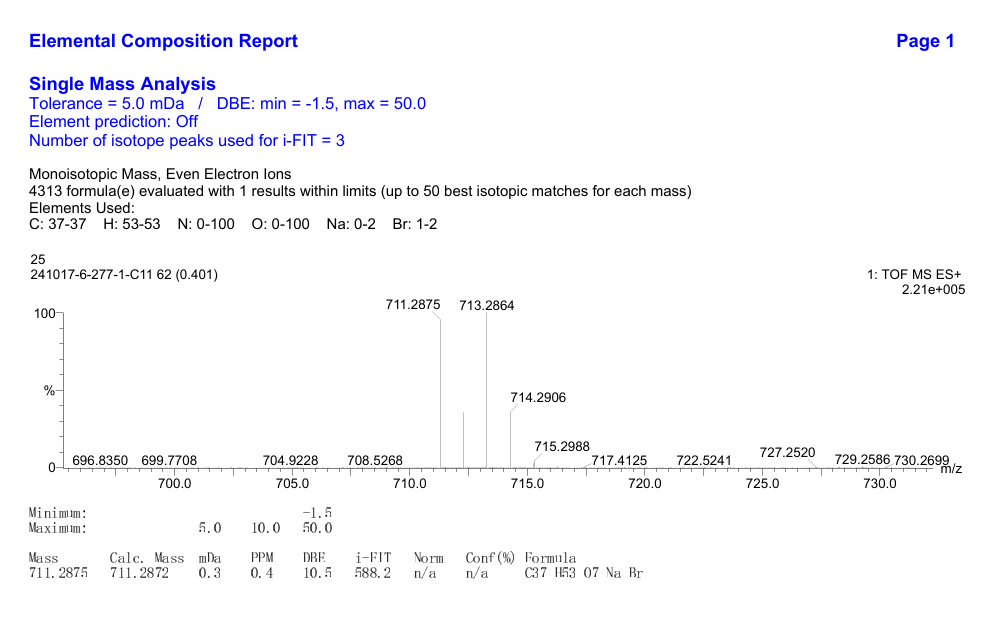


Figure.S69. HRMS of compound C11

### Compound R1

White powder, melting point: 234-236℃. ^1^H-NMR (CDCl_3_, 600 MHz, ppm): δ 0.42(d, *J* = 4.2 Hz, 1H, H-19b), 0.58(d, *J* = 4.2 Hz, 1H, H-19a), 0.97, 1.13, 1.15, 1.18, 1.24, 1.28, 1.31(s, 3H, -CH_3_×7), 2.35(d, *J* = 7.8 Hz, 1H), 2.60(q, *J* = 10.2 Hz, 1H), 3.57(t, *J_1_* = 3.0 Hz, *J_2_* = 10.2 Hz, 1H, H-6), 3.77(t, *J_1_* = 7.2 Hz, *J_2_* = 14.4 Hz, 1H, H-24), 4.68-4.73(m, 2H, H-3 and H-16), 6.47(d, *J* = 16.2 Hz, 1H, -CH=CH-), 7.38-7.39(m, 3H), 7.53-7.55(m, 2H, AR-H), 7.67(d, *J* = 15.6 Hz, AR-H); ^13^C-NMR (CDCl_3_, 150 MHz, ppm): δ 166.91(-C=O), 144.56, 118.90(-CH=CH-), 134.66, 130.31, 129.00, 128.21(AR-C), 87.22(C-20), 80.40(C-24), 81.53(C-3), 73.50(C-16), 71.96(C-25), 69.06(C-6), 57.60(C-17), 54.00(C-5), 47.25(C-8), 46.61(C-14), 46.14(C-15), 45.15(C-13), 40.78(C-4), 38.10(C-7), 34.56(C-22), 33.10(C-1), 31.92(C-12), 31.60(C-19), 29.50(C-2), 28.34(C-10), 27.92(C-28), 27.87(C-21), 27.03(C-11), 26.61(C-27), 26.02(C-26), 25.75(C-23), 21.64(C-9), 20.97(C-18), 20.21(C-30), 16.67(C-29); ESI-HRMS (m/z): calcd for C_39_H_56_O_6_Na^+^ [M+Na]^+^: 643.3975, found: 643.3978.


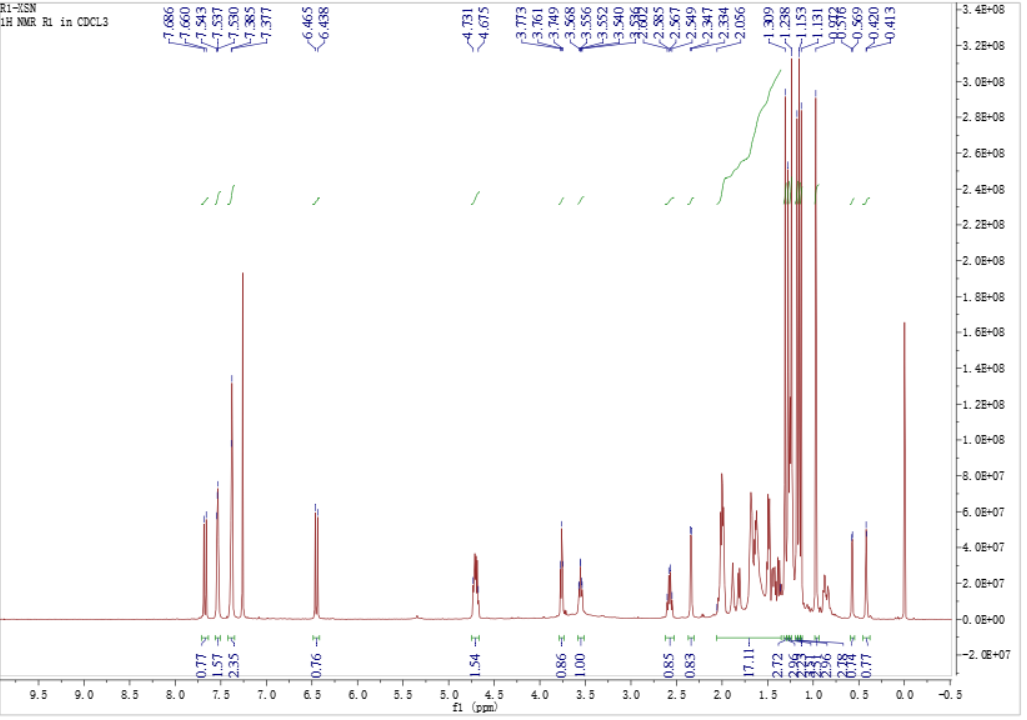


Figure.S70. ^1^H-NMR of compound R1


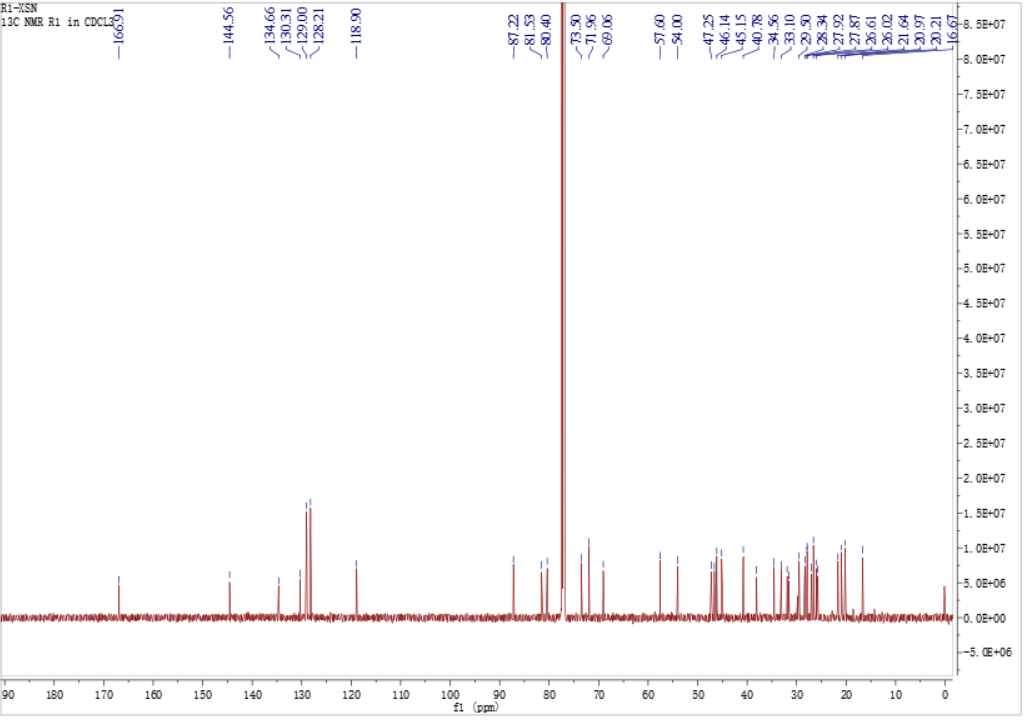


Figure.S71. ^13^C-NMR of compound R1


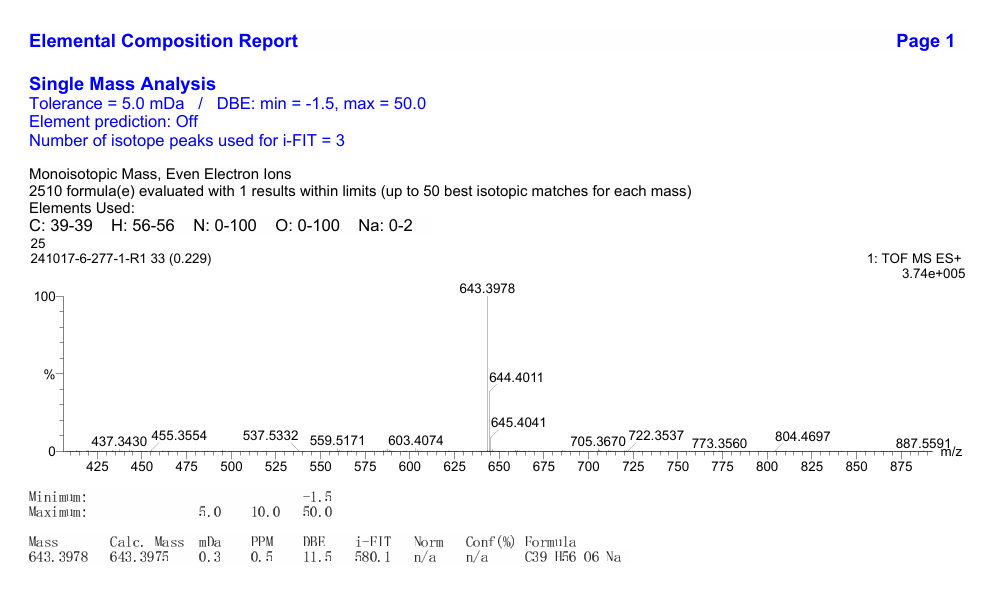


Figure.S72. HRMS of compound R1

### Compound R2

White powder, melting point: 233-235℃. ^1^H-NMR (CDCl_3_, 600 MHz, ppm): δ 0.42(d, *J* = 4.2 Hz, 1H, H-19b), 0.57(d, *J* = 4.2 Hz, 1H, H-19a), 0.97, 1.12, 1.14, 1.17, 1.23, 1.30, 1.33(s, 3H, -CH_3_×7), 2.35(d, *J* = 7.8 Hz, 1H), 2.61(q, *J* = 10.2 Hz, 1H), 3.56(t, *J_1_* = 3.0 Hz, *J_2_* = 9.6 Hz, 1H, H-6), 3.76(t, *J_1_* = 7.2 Hz, *J_2_* = 14.4 Hz, 1H, H-24), 4.67-4.72(m, 2H, H-3 and H-16), 6.38(d, *J* = 10.2 Hz, 1H, -CH=CH-), 7.08(t, *J_1_* = 8.4 Hz, *J_2_* = 17.4 Hz, 2H, AR-H), 7.51-7.53(m, 2H, AR-H), 7.64(d, *J* = 16.2 Hz, 1H, -CH=CH-); ^13^C-NMR (CDCl_3_, 150 MHz, ppm): δ 166.78(-C=O), 143.24, 118.64(-CH=CH-), 163.14, 130.91, 130.08, 130.02, 116.21, 116.07(AR-C), 87.19(C-20), 80.50(C-24), 81.50(C-3), 73.48(C-16), 71.95(C-25), 69.00(C-6), 57.61(C-17), 53.94(C-5), 47.35(C-8), 46.66(C-14), 46.11(C-15), 45.09(C-13), 40.77(C-4), 38.14(C-7), 34.55(C-22), 33.05(C-1), 31.91(C-12), 31.86(C-19), 29.52(C-2), 28.35(C-10), 27.99(C-28), 27.83(C-21), 27.02(C-11), 26.60(C-27), 26.03(C-26), 25.85(C-23), 21.63(C-9), 20.95(C-18), 20.21(C-30), 16.66(C-29); ESI-HRMS (m/z): calcd for C_39_H_55_O_6_FNa^+^ [M+Na]^+^: 661.3880, found: 661.3883.


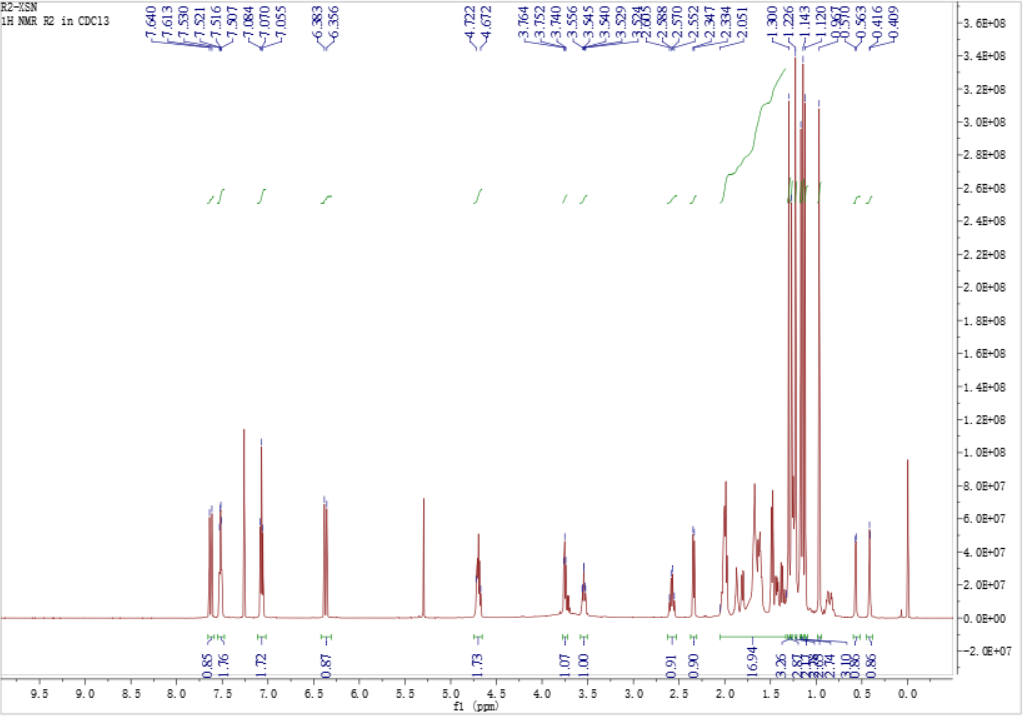


Figure.S73. ^1^H-NMR of compound R2


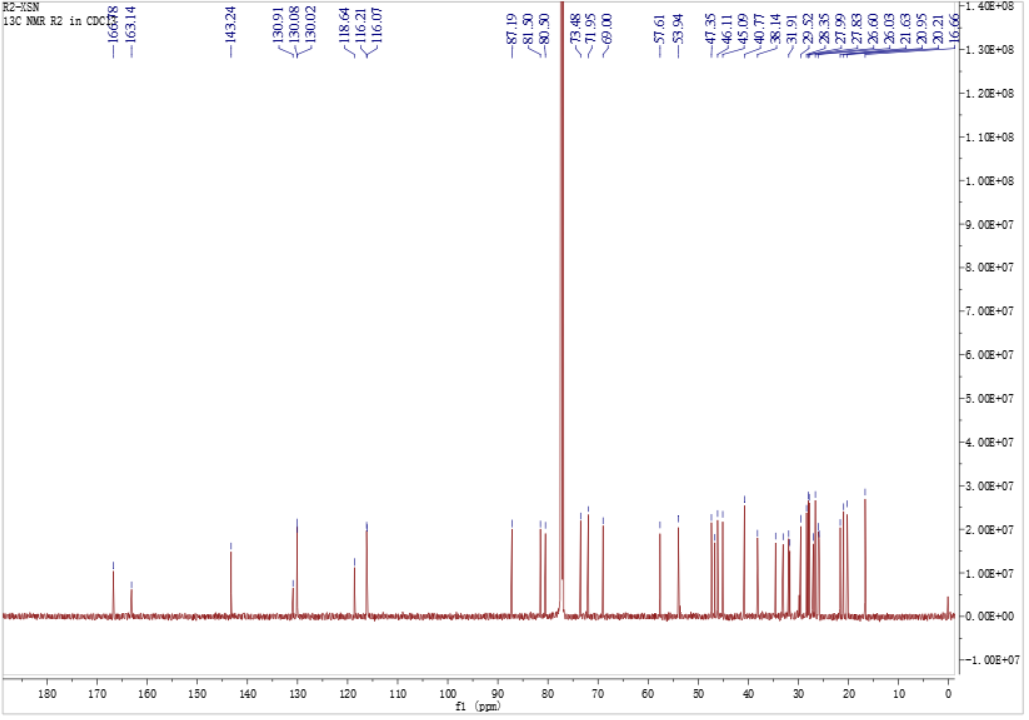


Figure.S74. ^13^C-NMR of compound R2


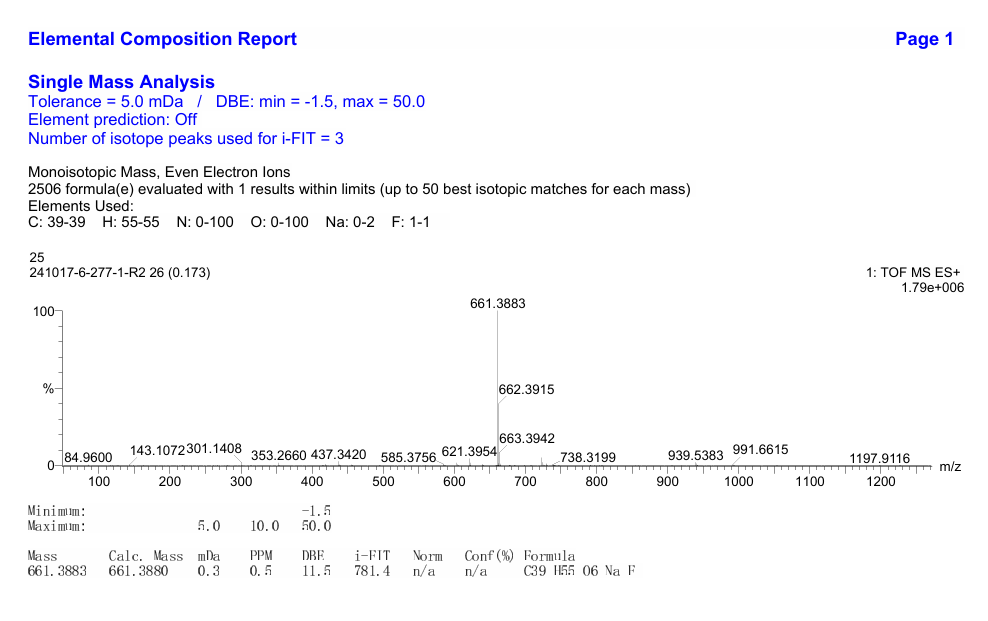


Figure.S75. HRMS of compound R2

### Compound R3

White powder, melting point: 236-238℃. ^1^H-NMR (CDCl_3_, 600 MHz, ppm): δ 0.42(d, *J* = 4.2 Hz, 1H, H-19b), 0.57(d, *J* = 4.2 Hz, 1H, H-19a), 0.97, 1.12, 1.16, 1.17, 1.24, 1.28, 1.31(s, 3H, -CH_3_×7), 2.34(d, *J* = 7.8 Hz, 1H), 2.60(q, *J* = 10.2 Hz, 1H), 3.52-3.58(m, 1H, H-6), 3.76(t, *J_1_* = 7.2 Hz, *J_2_* = 14.4 Hz, 1H, H-24), 4.67-4.73(m, 2H, H-3 and H-16), 6.43(d, *J* = 15.6 Hz, 1H, -CH=CH-), 7.36(d, *J* = 8.4 Hz, 2H, AR-H), 7.47(d, *J* = 8.4 Hz, 2H, AR-H), 7.63(d, *J* = 15.6 Hz, 1H, -CH=CH-); ^13^C-NMR (CDCl_3_, 150 MHz, ppm): δ 166.65(-C=O), 143.10, 119.48(-CH=CH-), 136.21, 133.15, 129.36, 129.29(AR-C), 87.21(C-20), 80.58(C-24), 81.53(C-3), 73.49(C-16), 71.96(C-25), 69.04(C-6), 57.61(C-17), 53.98(C-5), 47.28(C-8), 46.63(C-14), 46.12(C-15), 45.13(C-13), 40.76(C-4), 38.13(C-7), 34.56(C-22), 33.08(C-1), 31.91(C-12), 31.62(C-19), 29.85(C-2), 29.51(C-10), 28.35(C-28), 27.94(C-21), 27.01(C-11), 26.61(C-27), 26.03(C-26), 25.77(C-23), 21.63(C-9), 20.97(C-18), 20.21(C-30), 16.66(C-29); ESI-HRMS (m/z): calcd for C_39_H_55_O_6_ClNa^+^ [M+Na]^+^: 677.3585, found: 677.3586.


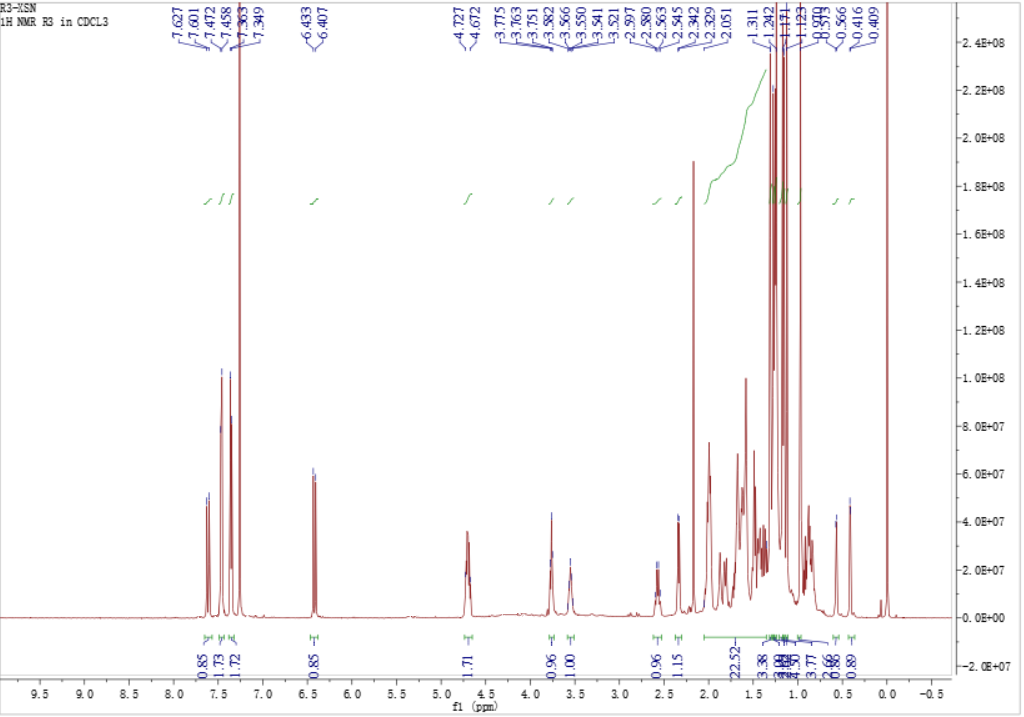


Figure.S76. ^1^H-NMR of compound R3


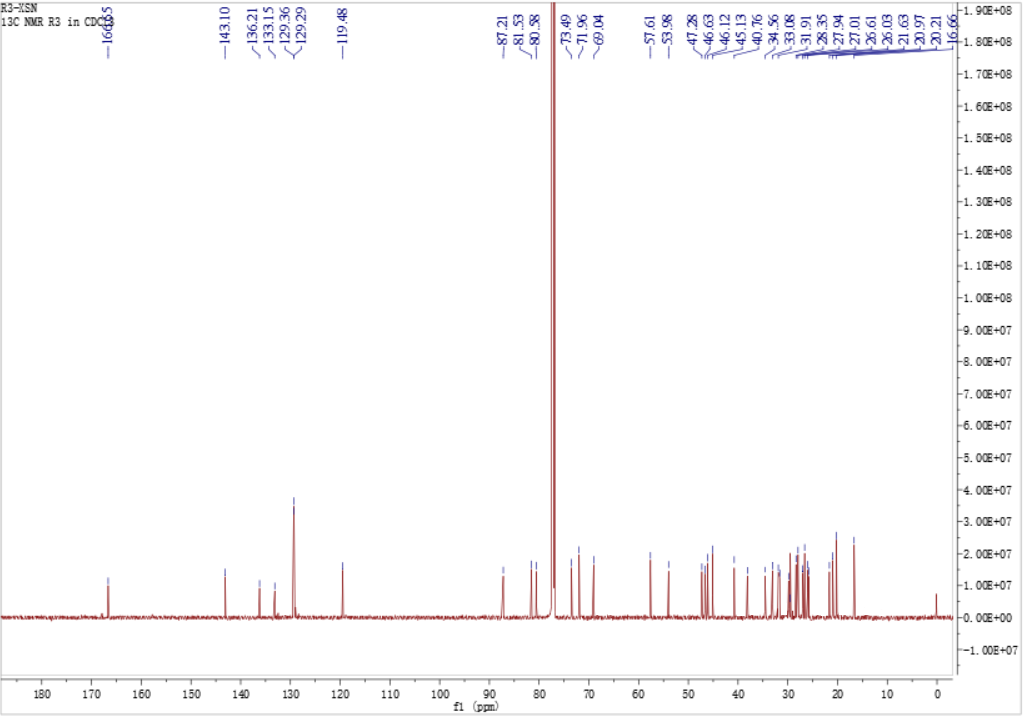


Figure.S77. ^13^C-NMR of compound R3


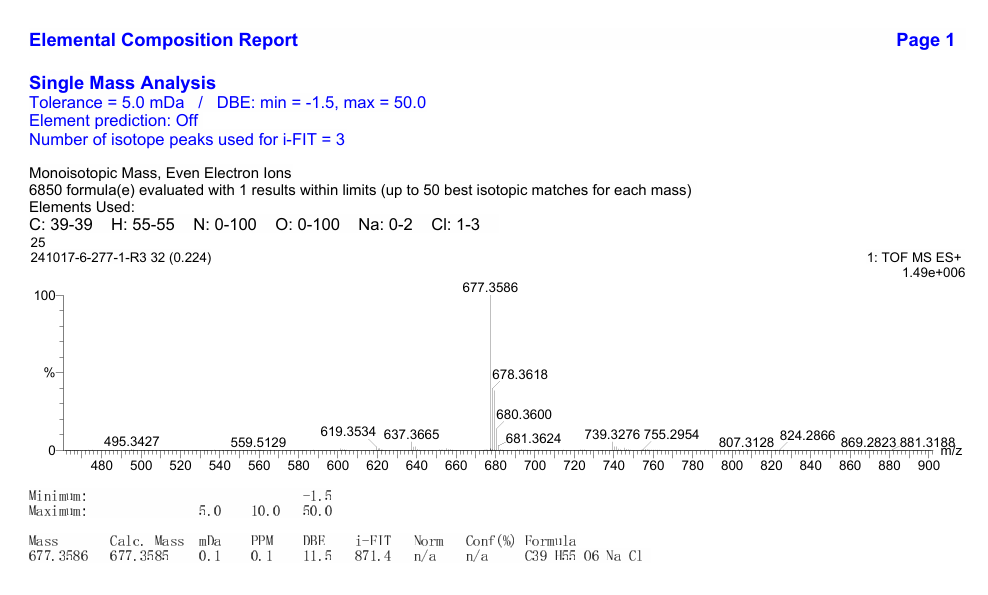


Figure.S78. HRMS of compound R3

### Compound R4

White powder, melting point: 238-240℃. ^1^H-NMR (CDCl_3_, 600 MHz, ppm): δ 0.42(d, *J* = 4.2 Hz, 1H, H-19b), 0.57(d, *J* = 3.6 Hz, 1H, H-19a), 0.97, 1.12, 1.14, 1.17, 1.23, 1.27, 1.30(s, 3H, -CH_3_×7), 2.35(d, *J* = 7.8 Hz, 1H), 2.61(q, *J* = 10.8 Hz, 1H), 3.56(t, *J_1_* = 3.0 Hz, *J_2_* = 10.2 Hz, 1H, H-6), 3.77(t, *J_1_* = 7.2 Hz, *J_2_* = 13.8 Hz, 1H, H-24), 4.67-4.72(m, 2H, H-3 and H-16), 6.45(d, *J* = 16.2 Hz, 1H, -CH=CH-), 7.40(d, *J* = 7.2 Hz, 2H, AR-H), 7.52(d, *J* = 7.8 Hz, 2H, AR-H), 7.61(d, *J* = 16.2 Hz, 1H, -CH=CH-); ^13^C-NMR (CDCl_3_, 150 MHz, ppm): δ 166.64(-C=O), 143.17, 119.59(-CH=CH-), 133.58, 132.24, 129.59, 124.55(AR-C), 87.19(C-20), 80.62(C-24), 81.51(C-3), 73.48(C-16), 71.96(C-25), 69.01(C-6), 57.61(C-17), 53.95(C-5), 47.32(C-8), 46.65(C-14), 46.12(C-15), 45.11(C-13), 40.76(C-4), 38.15(C-7), 34.55(C-22), 33.06(C-1), 31.91(C-12), 31.66(C-19), 29.84(C-2), 29.50(C-10), 28.36(C-28), 27.84(C-21), 27.01(C-11), 26.61(C-27), 26.03(C-26), 25.85 (C-23), 21.63 (C-9), 20.96 (C-18), 20.21 (C-30), 16.66(C-29); ESI-HRMS (m/z): calcd for C_39_H_55_O_6_BrNa^+^ [M+Na]^+^: 721.3080, found: 721.3082.


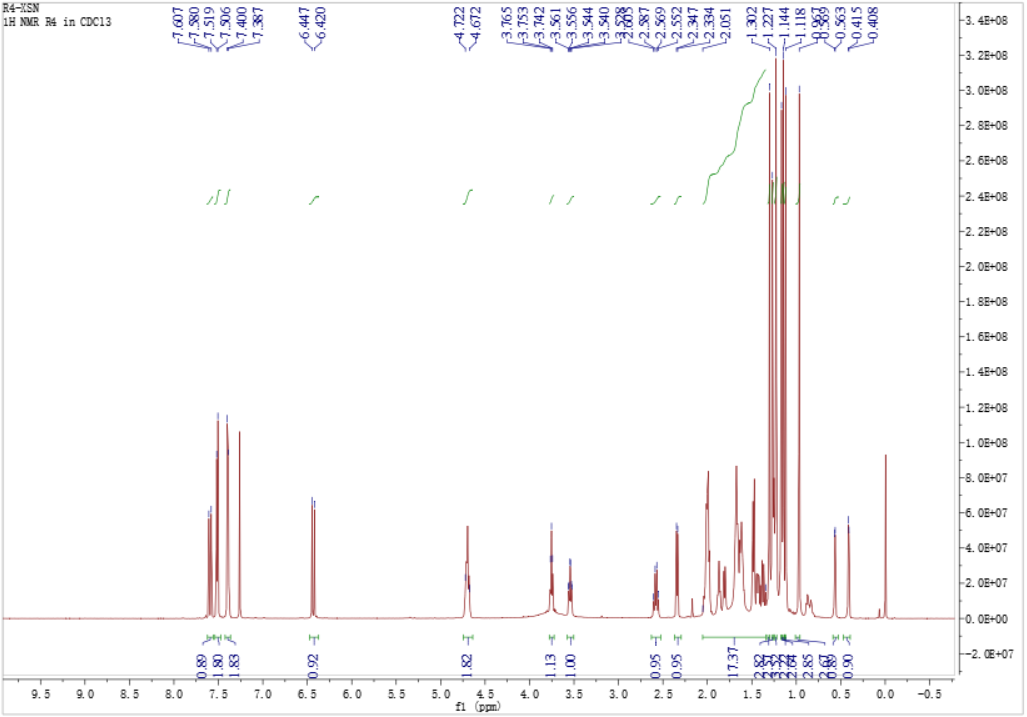


Figure.S79. ^1^H-NMR of compound R4


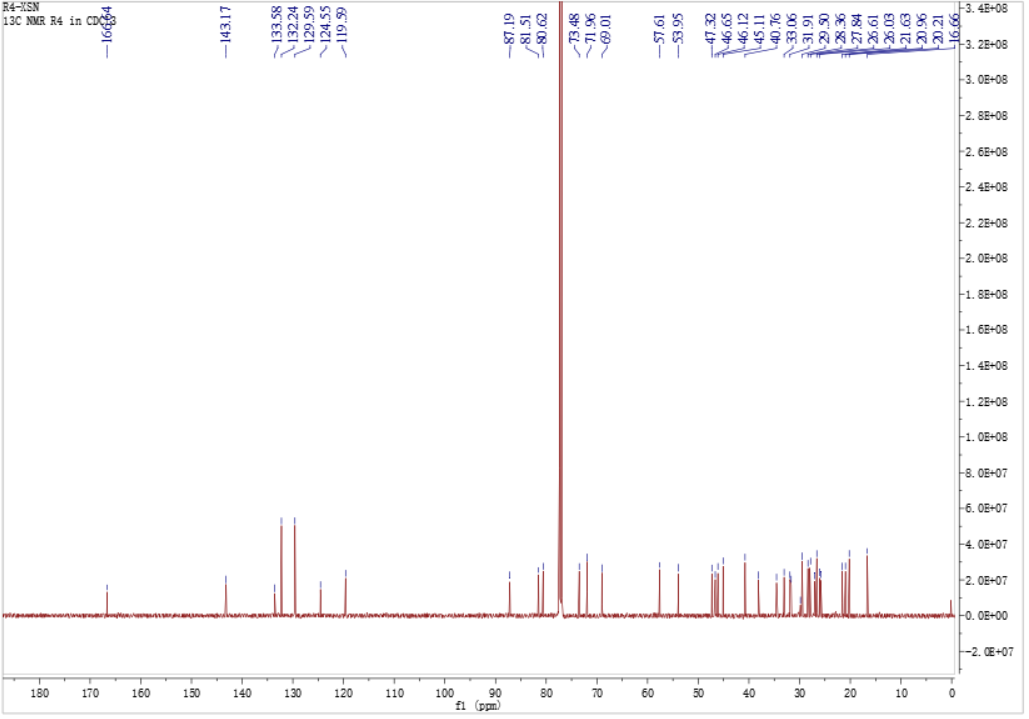


Figure.S80. ^13^C-NMR of compound R4


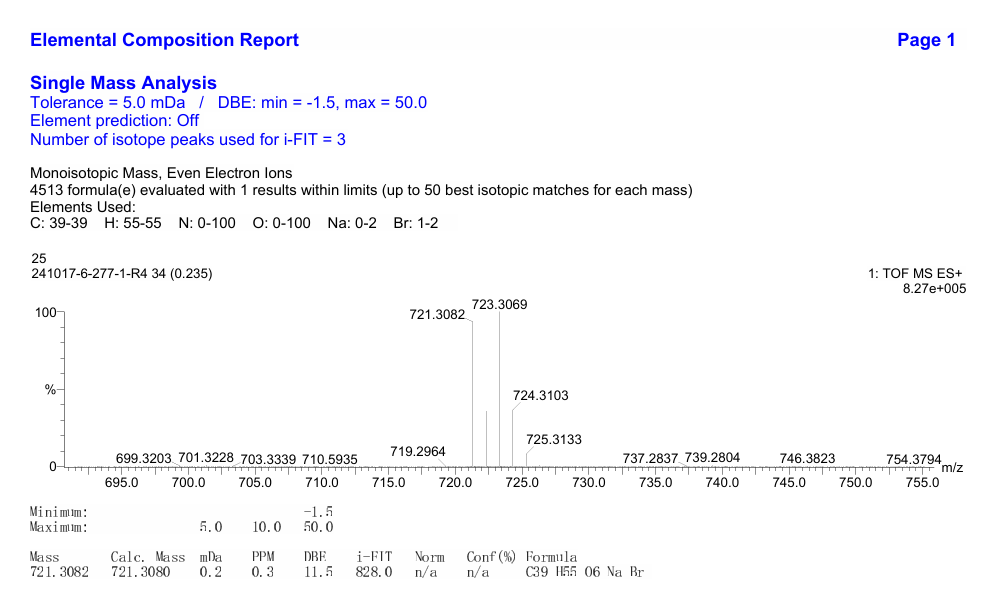


Figure.S81. HRMS of compound R4

### Compound S1

White powder, melting point: 244-246℃. ^1^H-NMR (CDCl_3_, 600 MHz, ppm): δ 0.42(d, *J* = 4.2 Hz, 1H, H-19b), 0.58(d, *J* = 4.2 Hz, 1H, H-19a), 0.96, 1.10, 1.16, 1.17, 1.24, 1.25, 1.31(s, 3H, -CH_3_×7), 2.34(d, *J* = 7.8 Hz, 1H), 2.59(q, *J* = 10.2 Hz, 1H), 3.57(t, *J_1_* = 3.0 Hz, *J_2_* = 10.2 Hz, 1H, H-6), 3.77(t, *J_1_* = 7.2 Hz, *J_2_* = 14.4 Hz, 1H, H-24), 4.71(q, *J* = 10.2 Hz, 1H, H-16), 4.78(dd, *J_1_* = 4.2 Hz, *J_2_* = 11.4 Hz, 1H, H-3); ^13^C-NMR (CDCl_3_, 150 MHz, ppm): δ 167.87(-C=O), 115.79(-CF_3_), 87.22(C-20), 81.52(C-24), 85.57(C-3), 73.46(C-16), 72.01(C-25), 68.85(C-6), 57.60(C-17), 53.60(C-5), 47.10(C-8), 46.50(C-14), 46.08(C-15), 45.16(C-13), 40.74(C-4), 38.24(C-7), 34.55(C-22), 33.05(C-1), 31.64(C-12), 31.54(C-19), 29.85(C-2), 29.10(C-10), 28.10(C-28), 27.90(C-21), 27.87(C-11), 26.66(C-27), 26.02(C-26), 25.68(C-23), 21.63(C-9), 21.11(C-18), 20.19(C-30), 16.28(C-29); ESI-HRMS (m/z): calcd for C_32_H_49_O_6_F_3_Na^+^ [M+Na]^+^: 609.3379, found: 609.3384.


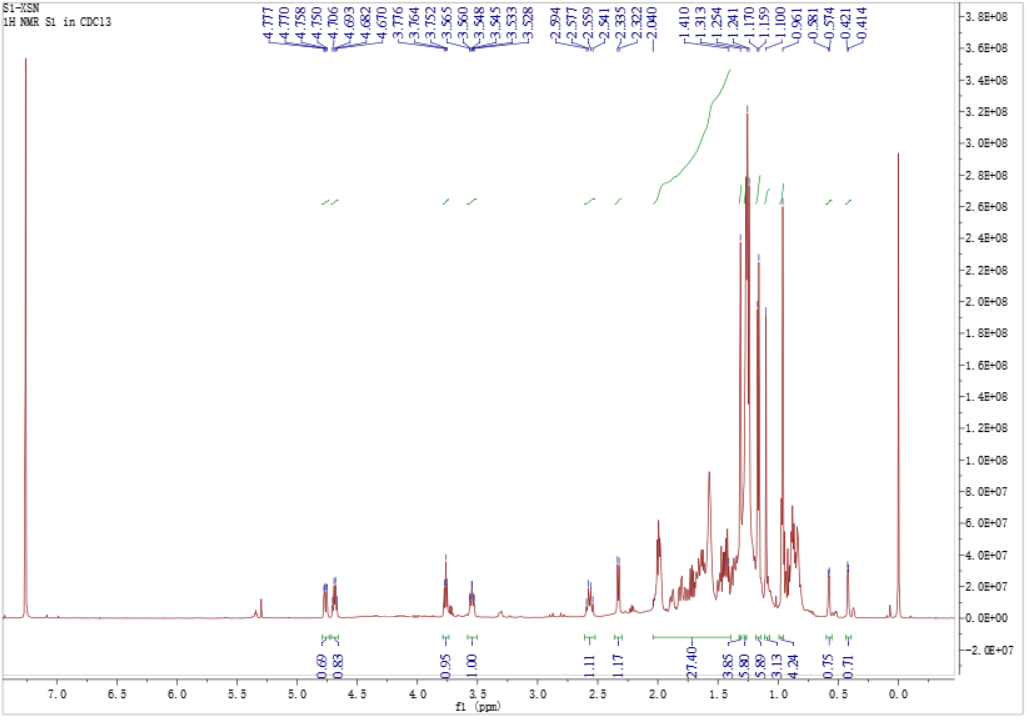


Figure.S82. ^1^H-NMR of compound S1


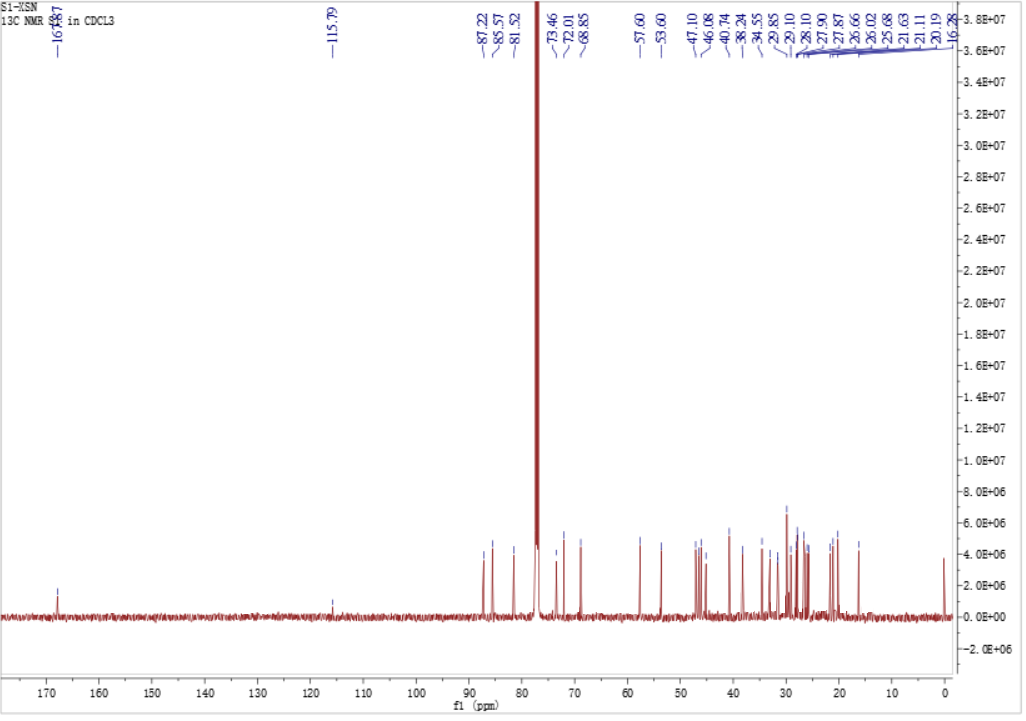


Figure.S83. ^13^C-NMR of compound S1


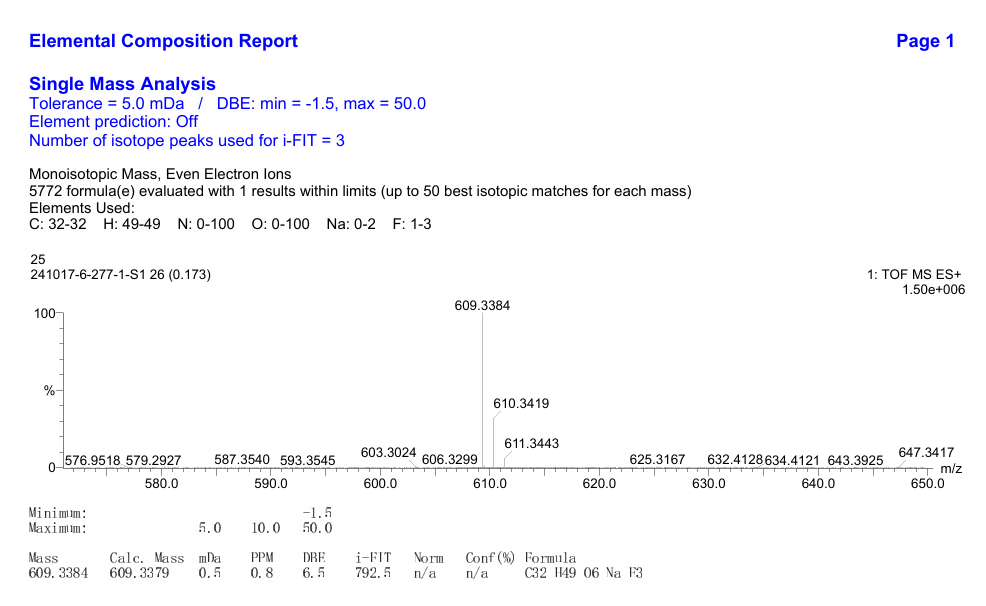


Figure.S84. HRMS of compound S1

### Compound S2

White powder, melting point: 248-250℃. ^1^H-NMR (CDCl_3_, 600 MHz, ppm): δ 0.43(d, *J* = 4.2 Hz, 1H, H-19b), 0.58(d, *J* = 4.2 Hz, 1H, H-19a), 0.96, 1.12, 1.14, 1.26, 1.30(s, 3H, -CH_3_×5), 1.22(s, 3H, -CH_3_×2), 2.34(d, *J* = 7.8 Hz, 1H), 2.60(q, *J* = 10.2 Hz, 1H), 3.56(t, *J_1_* = 2.4 Hz, *J_2_* = 9.6 Hz, 1H, H-6), 3.75(t, *J_1_* = 7.2 Hz, *J_2_* = 14.4 Hz, 1H, H-24), 4.68-4.71(m, 2H, H-3 and H-16); ^13^C-NMR (CDCl_3_, 150 MHz, ppm): δ 161.73(-C=O), 90.61(-CCl_3_), 87.16(C-20), 81.48(C-24), 86.68(C-3), 73.45(C-16), 71.99(C-25), 68.82(C-6), 57.60(C-17), 53.62(C-5), 47.29(C-8), 46.62(C-14), 46.08(C-15), 45.07(C-13), 41.11(C-4), 38.24(C-7), 34.55(C-22), 32.98(C-1), 31.70(C-12), 31.62(C-19), 29.25(C-2), 28.31(C-10), 28.00(C-28), 27.82(C-21), 26.62(C-11), 26.12(C-27), 26.05(C-26), 25.85(C-23), 21.63(C-9), 21.03(C-18), 20.20(C-30), 16.34(C-29); ESI-HRMS (m/z): calcd for C_32_H_49_O_6_Cl_3_Na^+^ [M+Na]^+^: 657.2492, found: 657.2495.


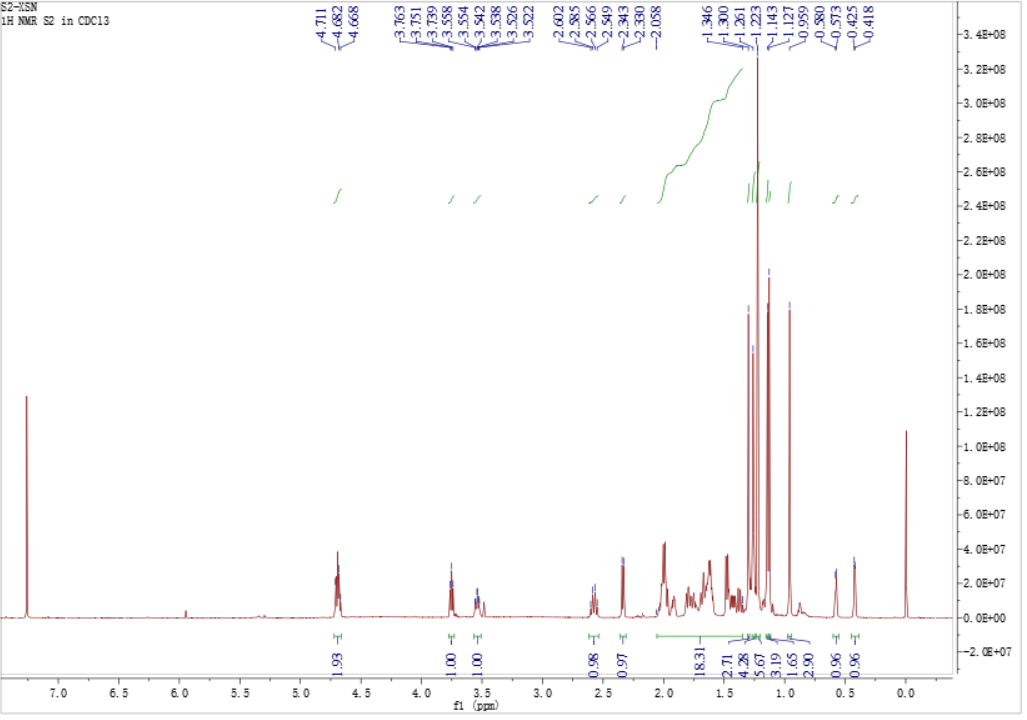


Figure.S85. ^1^H-NMR of compound S2


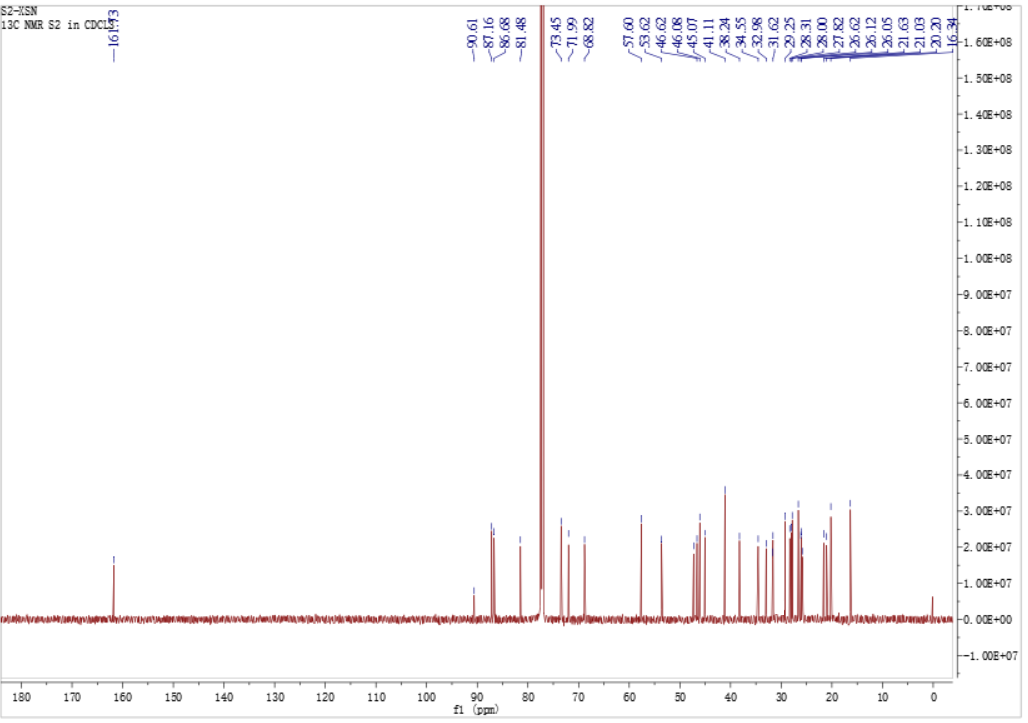


Figure.S86. ^13^C-NMR of compound S2


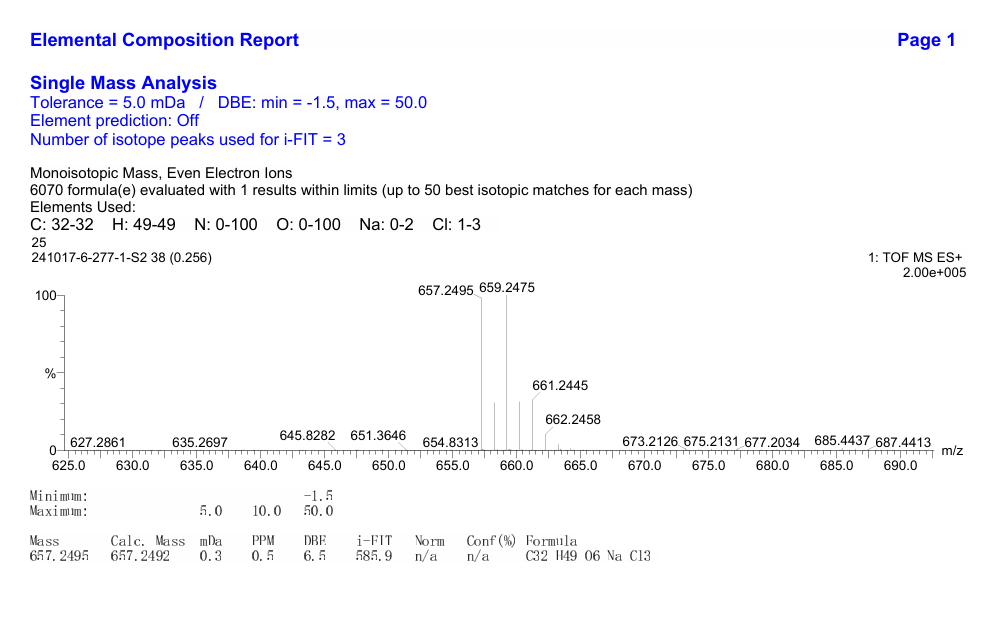


Figure.S87. HRMS of compound S2

Section 2


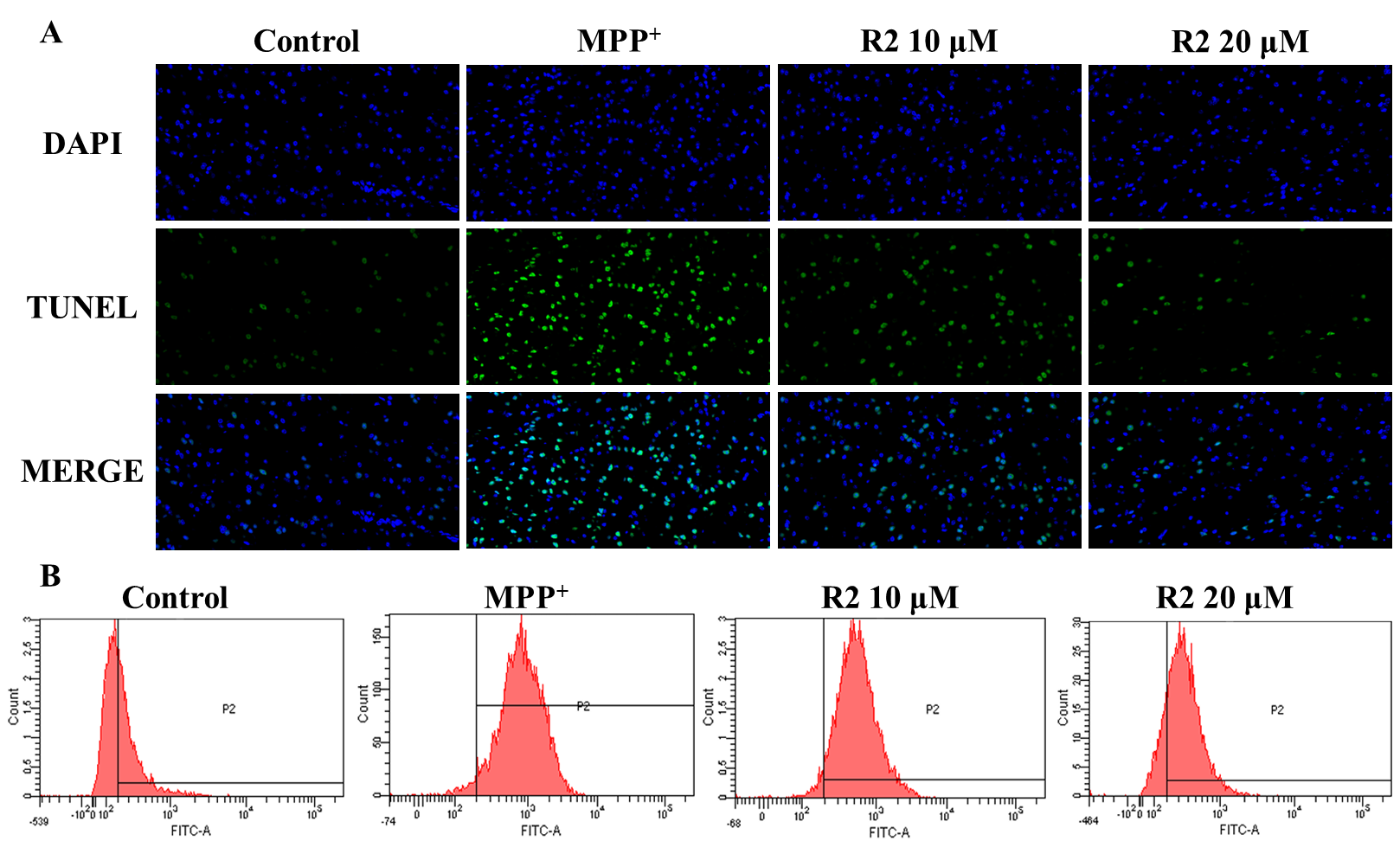


Figure S88. Compound R2 inhibits MPP^+^- induced reactive oxygen species and apoptosis in SH-SY5Y cells. A. TUNEL staining was used to detect the inhibition of MPP^+^- induced apoptosis in SH-SY5Y cells by compound **R2** (The scale is 100 μm); B. Flow cytometry detection of compound **R2** inhibiting MPP^+^- induced reactive oxygen species expression in SH-SY5Y cells


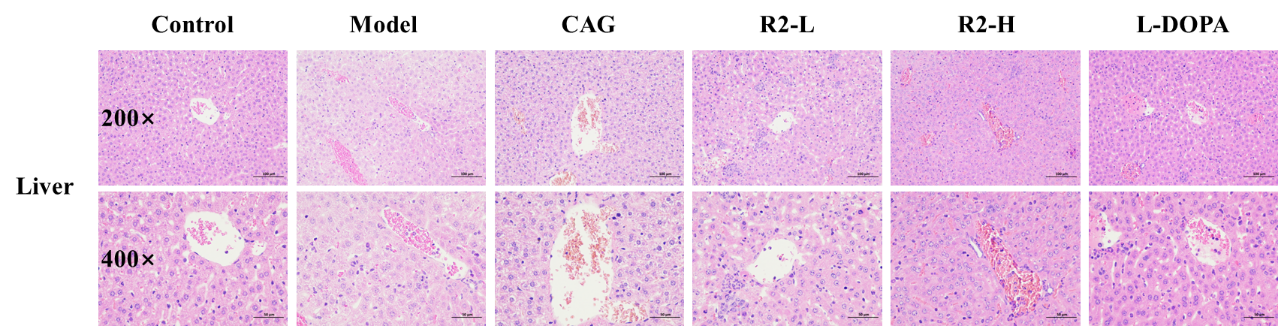


Figure S89. H&E staining analysis of liver tissue between different groups


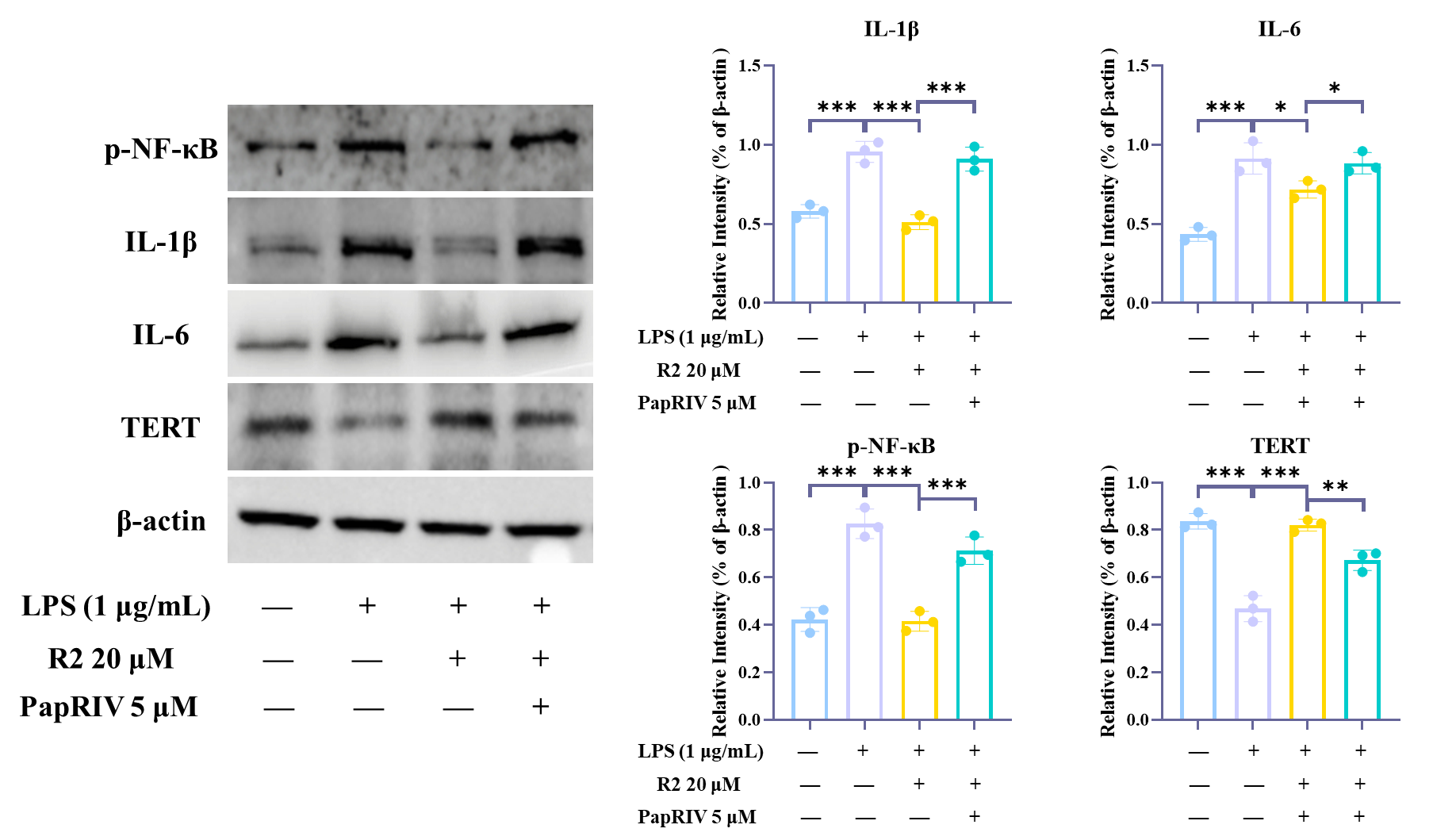


Figure S90. The anti-inflammatory effect and the promotion of increased TERT expression of compound **R2** can be reversed by NF-κB agonists (RapRIV). Data were expressed as means ± SDs of triplicate experiments performed independently. ^***^p<0.001, ^**^p<0.01, ^*^p<0.05.
